# Deciphering the influence of socioeconomic status on brain structure: insights from Mendelian randomization

**DOI:** 10.1038/s41380-025-03047-4

**Published:** 2025-05-13

**Authors:** Charley Xia, Yuechen Lu, Zhuzhuoyu Zhou, Mattia Marchi, Hyeokmoon Kweon, Yuchen Ning, David C. M. Liewald, Emma L. Anderson, Philipp D. Koellinger, Simon R. Cox, Marco P. Boks, W. David Hill

**Affiliations:** 1 Lothian Birth Cohort studies, University of Edinburgh, Edinburgh, UK; 2 Department of Psychology, School of Philosophy, Psychology and Language Sciences, University of Edinburgh, Edinburgh, UK; 3 Department of Biomedical, Metabolic and Neural Sciences, University of Modena and Reggio Emilia, Modena, Italy; 4 Department of Mental Health and Addiction Services, Azienda USL-IRCCS di Reggio Emilia, Reggio Emilia, Italy; 5 Department of Economics, School of Business and Economics, Vrije Universiteit Amsterdam, Amsterdam, The Netherlands; 6 MRC Integrative Epidemiology Unit, Bristol Medical School, University of Bristol, Oakfield House, Bristol, UK; 7 Division of Psychiatry, Faculty of Brain Sciences, University College London, London, UK; 8 Amsterdam UMC, Department of psychiatry, Amsterdam, The Netherlands

**Keywords:** Neuroscience, Predictive markers, Genetics, Psychology

## Abstract

Socioeconomic status (SES) influences physical and mental health, however its relation with brain structure is less well documented. Here, we examine the role of SES on brain structure using Mendelian randomisation. First, we conduct a multivariate genome-wide association study of SES using educational attainment, household income, occupational prestige, and area-based social deprivation, with an effective sample size of *N* = 947,466. We identify 554 loci associated with SES and distil these loci into those that are common across those four traits. Second, using an independent sample of ~35,000 we provide evidence to suggest that SES is protective against white matter hyperintensities as a proportion of intracranial volume (WMHicv). Third, we find that differences in SES still afford a protective effect against WMHicv, independent of that made by cognitive ability. Our results suggest that SES is a modifiable risk factor, causal in the maintenance of cognitive ability in older-age.

## Introduction

Socioeconomic status (SES) is a multi-dimensional construct influencing, and influenced by, multiple physical, socio-cultural, and environmental factors. Differences in SES are a determining factor of health where those from more advantaged backgrounds have a higher level of physical and mental health, where they live longer lives and are less likely to receive a dementia diagnosis [1–4]. These inequalities in physical health, and mental health are present across occupation, income, educational attainment, and measures of social deprivation [1, 5–7]. The communality of such findings highlights the need to assess the influence of SES using a multifactorial approach to examine the causes and consequences of differences in SES.

Genome-wide association studies (GWAS) examining traits such as income [8], educational attainment [9], and social deprivation [10] have identified hundreds of associated genetic loci with genetic correlations between SES related traits and physical health outcomes, indicating a common genetic aetiology between SES and physical health [8–10]. Furthermore, psychiatric traits including schizophrenia, major depressive disorder, and attention deficit hyperactivity disorder, as well as neurological disorders such as Alzheimer’s disease, early-onset stroke, and intra-cerebral haemorrhage also share genetic effects with measures of SES [11].

The value of these genetic data on SES related traits is underscored by its value in the examination of SES as a causal, and potentially modifiable, environmental risk factor through the use of Mendelian randomisation. For example, Davies et al. [12] found that an increase of 1 SD of education years led to a decrease in 1.00 kg/m^2^ decrease in BMI (95% CL: 0.06–1.93, *P* = 0.04), as well as an increase in the level of physical activity of participants of 0.31 days per week (95% CL: 0.09–0.54, *P* = 0.007). SES, as proxied using educational attainment, household income, and occupational attainment, have also been shown to be causally linked to longer parental lifespan [13]. Furthermore, Ye et al. [13] used Multivariable Mendelian randomisation to show that educational attainment still exerted its influence on parental lifespan following adjustment for household income and occupational attainment, highlighting the value of MR methods to differentiate between correlated environmental risk factors. More recently, genetic data has been used to show that poverty is a potentially modifiable environmental risk factor causal in mental health traits including schizophrenia and attention deficit hyperactivity disorder [14].

However, the following are some fundamental gaps in our understanding of the relationship between SES and brain structure. First, do different indicators of SES confer the different levels of risk or is SES best captured using a single factor? Second, is there evidence for causality in the relationship between SES and brain morphology, particularly in regard to brain health in older age? Third, to what extend do differences in cognitive ability explain the relationship between SES and brain morphology?

Importantly, the use of brain morphology as an outcome in MR can allow for the risk factors of late-life cognitive ability that act on cognitive decline in adulthood to be distinguished from those that differentiate the trajectory of cognitive growth through childhood. The importance of which is underscored in the context of dementia which, whilst typically diagnosed using cognitive tests such as the Mini-Mental State Examination [15], is distinguished from other neurodevelopmental disorders (such as intellectual disability) by a progressive later-life loss of cognitive ability that affects daily life [16]. As such, risk of dementia can be seen to be composed of two components: cognitive development influencing the level of cognitive ability prior to the onset of cognitive decline and, the rate at which decline occurs. Whilst large GWAS of cognitive decline are currently lacking, MR combined with GWAS conducted on frank indictors of brain ageing, such as white matter hyperintensities [17], can be used to identify potentially modifiable risk factors causal in brain ageing.

In the current study, we combine multivariate analysis with MR to examine the bidirectional effects between SES and brain morphology, and to identify potentially modifiable risk factors of age-related brain change associated with cognitive development and cognitive decline. First, we conducted a factor analysis and discovered that, while occupational prestige (OP, *N* = 279,644), household income (HI, *N* = 781,627), educational attainment (EA, *N* = 753,152), and social deprivation (SD, *N* = 440,350) show weak correlations at the phenotypic level, they share a similar genetic architecture. Recognising this shared genetic structure, we performed a multivariate GWAS incorporating all four traits to extract the common factor of SES, resulting in a better powered genome-wide association with a sample size of 947,466. We refer to this common factor as gSES. The use of these four measures in a multivariate framework allows for the assessment of heterogeneous effects across each trait in conjunction with an investigation of effects that act on the individual, as well as the household, and geographical area in which one resides. Thus, effects can be categorised as common across measures of SES or unique to specific trait used to construct gSES. Second, to examine the bidirectional effects of gSES on brain structure we use two-sample MR on 13 brain imaging phenotypes sourced from an independent sample of ~35,000 UKB participants and find gSES has a likely causal effect on white matter hyperintensities, a known risk factor of cognitive decline and dementia in older age. Finally, we show a direct effect of gSES on white matter hyperintensities, independent of the effects of cognitive ability.

A less technical overview of the paper, as well as how it should and should not be interpreted, can be found in our FAQ (Supplementary Note 1) and in Box 1.

Box 1 Genetics and Socioeconomic status: (mis)applications and implications Due to the potential for misunderstanding the role of genetics on human behavioural traits it is important to understand how to correctly interpret our findings and report them in an ethically responsible manner [62]. This section (in addition to the FAQ in Supplementary Note 1) serves as a guide for understanding how genetic differences can be linked to socioeconomic status differences and highlight the value of genetic data as a tool to examine environmental influences in human trait variation.

***What did we do?***

Our study used two genome-wide association studies (GWAS), and Mendelian randomisation (MR). GWASs are used to capture the relationship between millions of genetic variants and a trait of interest [63]. We were interested in examining genetic variants linked with four social science traits: occupational prestige, household income, educational attainment, and social deprivation. GWAS have been used to capture genetic contributors to these measures previously. These genetic variants also overlap with those associated with health outcomes such as longevity, and cardiovascular disease [8, 10, 20]. One explanation for these shared genetic effects across occupational prestige, household income, educational attainment, and social deprivation and health is that the same genetic variants independently contribute to both health and SES; a process referred to as horizontal pleiotropy. However, it is also possible that differences in SES have a causal effect on health outcomes; referred to as vertical pleiotropy. This means that a genetic variant could be associated with both SES and health outcomes *because* differences in SES cause differences in health. MR allows us to detect vertical pleiotropy within our data.

***Why use genetic data to study SES?***

The use of genetic data allows us to utilise the MR method to examine instances of vertical pleiotropy (i.e. where SES is a causal factor on brain structures related to ageing). To examine causality, it is typical to design a randomized control trial (RCT) where participants are randomly allocated to a control or treatment group. However, it is not possible or ethical to randomise SES within a population. MR has been deemed analogous to RCTs because offspring randomly inherit one genetic variant (allele) from each parent [64]. Thus, as with RCTs, neither confounding nor subsequent disease can affect this randomisation, minimising potential bias and improving our ability to make causal inferences.

***Genetic determinism, genetic distractionism, and policy fatalism***

The communication of the results of genetic studies on factors that influence human behaviour, particularly those linked to measures of socio-economic status, should be disseminated in a responsible manner, and call attention to hypotheses that it does not and cannot support. Consistent with this goal, it is important to emphasise that neither an individual’s genetic inheritance nor their societal environment dictates their intrinsic value. Furthermore, the genetic architecture of SES traits is neither fixed, nor universal across cultures or time periods [58, 59, 65]. Rather, GWASs on measures of SES capture traits that linked to SES, only in the environment in which they are measured, meaning that these genetic influences are as fluid as the traits linked to measures of SES (such as health, skills valued in a labour market, or risk-taking tendencies).

This study provides support for the hypothesis that a lower SES is one of the causal risk factors in the accumulation of whiter matter hyper intensities (WMH). This link is not genetic in origin and does not suggest that the link between SES and brain ageing differences is due to the same genes influencing both traits (horizontal pleiotropy). Rather, through the use of genetic data we find evidence that SES, as a potentially modifiable environmental risk factor, is one potential causal agent in observed WMH differences. Importantly, our results are suggestive that by environmentally modifying one’s SES, there may be a corresponding effect on their WMH.

## Materials and methods

### Samples

European samples from UK Biobank [18] were retained if they had genetic information available, sex that was consistent between self-reported and inferred using genotype, no sex chromosome aneuploidies, not having been detected as extreme outliers of heterozygosity and missingness as defined in sample QC file by UKB, having not withdrawn consent, and having a genotyping rate greater than 0.9. This resulted in 440,964 participants being available for analysis. European ancestry was identified from the UK Biobank participants that self-reported as white. Principal components (PC) were derived from the genotype data and participants were excluded if they were outside of a mean ± 3 standard deviations from the first six principal components. For our general factor phenotypic of SES, we used all participants who had provided phenotypic data on at least one of our measures of SES.

**Fig. 1:**
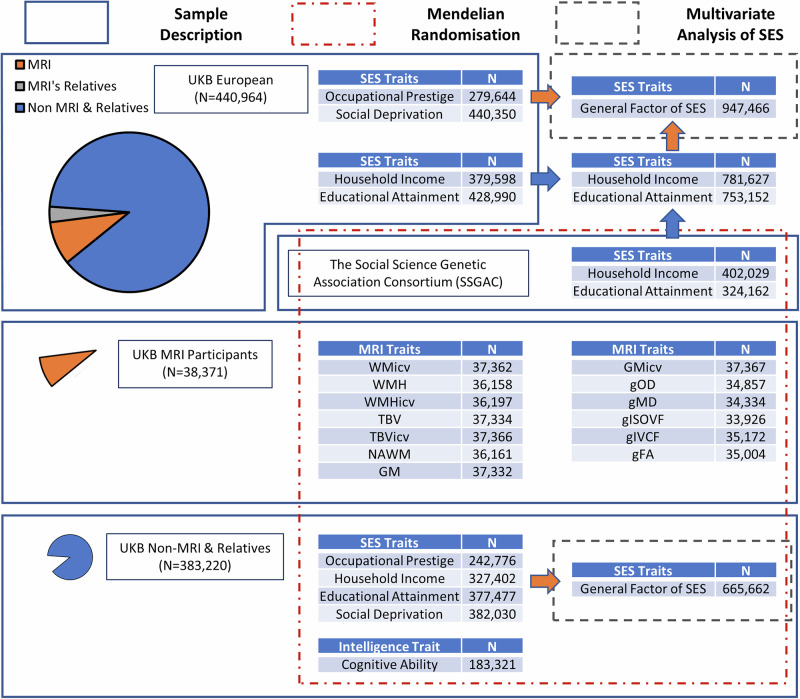
Shows GWAS sample size, relationship among samples, and analytic plan. Blue arrow refers to meta-analysis in METAL. Orange arrow refers to common factor GWAS in GenomicSEM. N refers to sample size. In common factor GWAS, N refers to effective sample size. TBV total brain volume, TBVicv total brain volume as a proportion of intracranial volume, GM total grey matter volume, GMicv total grey matter volume as a proportion of intracranial volume, WMH white matter hyperintensity volume WMHicv white matter hyperintensity volume as a proportion of intracranial volume NAWM normal-appearing white matter volume, WMicv white matter volume as a proportion of intracranial volume. gFA The first unrotated component of fractional anisotropy properties. gMD The first unrotated component of mean diffusivity properties. gICVF The first unrotated component of intra-cellular volume fraction properties. gISOVF The first unrotated component of isotropic volume fraction properties. gOD The first unrotated component of orientation dispersion properties.

For our Mendelian randomisation analysis, we derived two independent samples using the participants of UK Biobank. The brain imaging subset which consisted of 38,371 participants that had at least one MRI phenotype, and the general genetic factor of SES (gSES), and cognitive ability group that consisted of 383,220 participants who did not have any MRI phenotype and did not have any relatives in the outcome set based on as defined by UK Biobank. See Fig. 1 for more details.

### Ethics approval and consent to participate

Ethical approval was granted by UK Biobank and this project was conducted under UK Biobank application 10279. All methods were performed in accordance with the relevant guidelines and regulations. Each GWAS dataset included received approval from their respective ethics committees or institutional review boards, with informed consent obtained from participants.

### Measures

Income was measured at the level of the household (HI, *N* = 379,598, MR sample = 327,402 excluding MRI participants and their relatives), which was measured in UK Biobank using an ordinal scale of 1–5 corresponding to the participants self-reported level of household income before tax (1 = < £18,000, 2 = £18,000–£30,999, 3 = £31,000–£51,999, 4 = £52,000–£100,000, 5 = > £100,000).

Social deprivation was measured using the Townsend deprivation index (TS, *N* = 440,350, MR sample = 382,030 excluding MRI participants and their relatives). The Townsend deprivation index is an area-based measure of SES derived using the participant’s postcode. Townsend scores were calculated immediately prior to joining UK Biobank and are formed from four measures: the percentage of those aged 16 or over who are unemployed, the percentage of households who do not own a car, do not own their own home, and which are overcrowded. Scores were multiplied by −1 when used for deriving phenotypic and genetic correlations as well as for use in in Genomic SEM to ensure that the direction of effect was the same across each measure of SES (i.e., a greater score indicates a higher level of SES). However, for use in Mendelian randomisation the original direction of effect is retained where a greater score indicates higher level of deprivation (i.e. a lower level of SES).

Occupational prestige was measured using the Cambridge Social Interaction and Stratification Scale (CAMSIS, *N* = 279,644, MR sample = 242,776 excluding MRI participants and their relatives) and was derived using job code at visit (data field 20277) in UK Biobank transformed using the method described by Akimova et al. [19]. In brief, the CAMSIS uses the idea that social stratification acts to create differential association where partners and friends are typically selected from within the same social group. Thus, CAMSIS captures the distance between occupations by measuring the frequency of social interactions between them.

Educational attainment (EA, *N* = 428,990, MR sample = 377,477 excluding MRI participants and their relatives) was measured by transforming educational qualifications found in UK Biobank to a binary variable where ‘1’ indicated that the participant had attained a university level degree and ‘0’ indicated that they had not.

Due to the high genetic correlations between occupational prestige (*r*_*g*_ = 0.69, SE = 0.02), household income (*r*_*g*_ = 0.58, SE = 0.02), educational attainment (*r*_*g*_ = 0.67, SE = 0.02) and social deprivation (*r*_*g*_ = 0.27, SE = 0.03) with cognitive ability found here and in previous studies [20, 21] and the finding that cognitive ability is a likely causal variable in differences in income and educational attainment in the UK [8, 12, 22], cognitive ability was also included as an exposure variable. Cognitive ability was measured using the verbal-numerical reasoning test (VNR, *N* = 183,321 excluding MRI participants and their relatives) in UK Biobank. This test consists of 13 (14 for the online version of the test) multiple-choice questions (six verbal and seven numerical) which are to be completed within a two-minute time limit. A participant’s score on each of the questions is then summed to provide an overall measure of the participant’s level of cognitive ability. Participants either completed the VNR test at the assessment centre at one of four time points or completed an online version of the VNR test. If participants took the VNR at multiple time points, only the first instance of the test was used to avoid capturing practise effects in the assessment of the participant’s level of cognitive ability.

Brain structural and diffusion neuroimaging data were acquired, processed and QCd by the UK Biobank team as Imaging Derived Phenotypes (IDPs) according to open access publications [23, 24] and online documentation (https://biobank.ctsu.ox.ac.uk/crystal/crystal/docs/brain_mri.pdf). Global macrostructural outcomes of interest were selected as they have been shown to be associated with both ageing and differences in cognitive ability [25, 26]. These global macrostructural outcomes of interest were: total brain volume (TBV), total brain volume as a proportion of intracranial volume (TBVicv), total grey matter volume (GM), total grey matter volume as a proportion of intracranial volume, (GMicv), white matter hyperintensity (WMH) volume, white matter hyperintensity volume as a proportion of intracranial volume (WMHicv), normal-appearing white matter volume (NAWM, total white matter volume–WMH), white matter volume as a proportion of intracranial volume (WMicv). In addition, we include five global white matter microstructural measures derived from twenty-seven major white matter tracts, for which five tract-averaged white matter diffusion properties were available as IDPs (UK Biobank Category ID 135): fractional anisotropy (FA), mean diffusivity (MD), intra-cellular volume fraction (ICVF), isotropic volume fraction (ISOVF) and orientation dispersion (OD). We ran five PCAs of all 27 tracts, a separate model for each of the five properties. The first unrotated component of each PCA was extracted for further analysis, yielding five global white matter measures (gFA, gMD, gICVF, gISOVF and gOD) which explained 44, 50, 68, 37 and 26% of the variance, respectively. These derived variables capture the variance that is shared across each regional white matter property, providing a global measure of white matter integrity. As with the total brain, white matter hyperintensity, and grey matter volume traits described above, these global measures of white matter integrity capture age-related deterioration of white matter in healthy, non-clinical populations [27]. Prior to analysis, participants with the following conditions (UK Biobank field ID 20002.2) were excluded at the outset: dementia, Parkinson’s disease, Guillain-Barré, multiple sclerosis, stroke, brain haemorrhage, brain/intracranial abscess, cerebral aneurysm, cerebral palsy, encephalitis, epilepsy, head injury, infection of the nervous system, ischaemic stroke, meningioma, meningitis, motor neurone disease, spina bifida, subdural haematoma, subarachnoid haemorrhage, transient ischaemic attack, brain cancer, meningeal cancer, other demyelinating or other chronic/neurodegenerative illness, or other neurological injury/trauma. Outliers (>4SDs from the mean, which represented <0.1% of the data in all cases) were then removed from all IDPs prior to analyses. As detailed above, there was no sample overlap between the participants who provided brain imaging data and the participants who provided data pertaining to gSES.

Detailed information on sample size and which traits were involved in these analyses are provided in Fig. 1.

### Genome-wide association studies

Genome-wide association studies (GWASs) were conducted in REGENIE v3.1.3 [28]. REGENIE uses a two-step approach to account for sample relatedness and population structure. In the first step, a whole genome regression model was fit to each trait (Exposures and outcomes) using 564,253 genotyped variants. These variants have minor allele frequency (MAF) > 0.01, call rate > 0.9, and Hardy-Weinberg Equilibrium of HWE-*p* value > 10^−15^.

In the second step, an association test was performed for each of the 13,192,861 imputed variants using a LOCO (leave-one-chromosome out) scheme. These variants have MAF > 0.001 and INFO > 0.8. For binary phenotypes (i.e., Educational attainment), firth logistic regression test was performed in the second step.

The per-chromosome LOCO genomic predictions produced in the first step were fitted in the second step to account for sample relatedness and population structure. In addition, sex, age at assessment, assessment centres, genotyping array, genotyping batch, and the first 40 PCs derived from genotype data were fitted as covariates in both steps. For cognitive ability, participants’ who took the VNR at an assessment centre were analysed together including time point (1–4) as an additional covariate before being meta-analysed with the participants whose first instance of taking the VNR was online. Regarding brain imagining phenotypes, three-dimensional head position along the X, Y, and Z axis were fitted as extra covariates. For TBV height was fitted as an additional covariate and for GM and NAWM both height and TBV were fitted. For VNR, the GWASs were performed in participants who took the test in the assessment centre, and those took the online test separately, before combining the results with an inverse variance weighted model [29].

### Linkage disequilibrium score regression (LDSC)

Using the 1000 G European reference panel LDSC [30] was performed to estimate the heritability of the exposure and outcome traits. In addition, the intercept of each LDSC regression was used to examine the GWAS association test statistics for inflation due to factors other than polygenicity.

### Phenotypic and genomic structural equation modelling

Phenotypic common factor of SES was derived in R [31] using factor analysis in *psych* [32] package using standardised phenotypes. A total of 248,480 participants provided data pertaining to their occupational prestige, household income, educational attainment, and social deprivation phenotypes.

The genetic factor structure was assessed using Genomic SEM and GWAS data conducted on occupational prestige (*N* = 279,644), household income (*N* = 781,627), educational attainment (*N* = 753,152), and social deprivation (*N* = 440,350) phenotypes (Fig. 1). Note that as sample overlap is controlled for in Genomic SEM these samples sizes are larger than those used in our Two-sample Mendelian randomisation analysis described above. Regarding genetic common factor of SES, we used genomic structural equation modelling [33] to derive LDSC—based [34] genetic correlations and covariances between occupational prestige, household income, educational attainment, and social deprivation. Next, the covariance structure between each of the four traits used to derive a genomic structural equation model to examine their loading on a single factor of SES. This common factor model was ran using SNPs from occupational prestige, household income, educational attainment, and social deprivation where MAF > 0.01 and INFO > 0.9. Next, we performed a multivariate GWAS using genomic SEM where 7,462,121SNPs with MAF > 0.01 and INFO > 0.6 were included to derive genome-wide summary statistics describing each SNPs association with the common factor of SES, termed gSES. In addition, we derived genome-wide heterogeneity (Q) statistics describing the degree to which a given SNP is likely not acting on single latent factor of SES. To examine the goodness-of-fit of the phenotypic model and the model derived using Genomic SEM the standardised root mean square residual (SRMR), model χ2, and the comparative fit index (CFI) were used. We used the criteria proposed by Hu and Bentler [35] to determine a good fit: CFI > 0.95, SRMR < 0.08.

### Meta analysis of income and education

Data provided by the Social Science Genetic Association Consortium (SSGAC) was used to add power to the gSES as well acting as a replication sample for educational attainment and household income and for use in Multivariable Mendelian Randomisation (MVMR). For both meta-analyses, METAL [36] was used to conduct a sample size weighted meta-analysis from which Beta values and standard error obtained using the following equation as provided by Zhu et al. [37].

$$\beta =\frac{Z}{\sqrt{2\times {MAF}\times \left(1-{MAF}\right)\times (N+{Z}^{2})}}$$

$${SE}=\frac{1}{\sqrt{2\times {MAF}\times \left(1-{MAF}\right)\times (N+{Z}^{2})}}$$

(Where ${MAF}$ is the minor allele frequency, $N$ is the sample size, and $Z$ is the test-statistics.)

### Loci identification and overlap

For each trait, genomic risk loci were identified by FUMA [38] (version v1.3.6a) using 1000 G EUR reference panels. Briefly, FUMA performed two LD clumpings. The first clumping was designed to define independent signals (genome significant SNPs at *P* < 5 × 10^−8^) with *r*^*2*^ > 0.6. In the second clumping, independent signals were clumped into one genomic locus if the *r*^2^ between two signals is >0.1 or two signals are within 250 kb. The SNPs clumped into each genomic locus naturally formed its physical boundary.

We compared the positions of genomic loci between two traits locus-by-locus. We define that a locus of trait A overlaps with trait B, if the positions of any trait B loci overlap with the position of that trait A locus. For the general factor of SES, we define a locus as unique to general SES if that locus does not overlap with any of the four contributing traits. For the four contributing traits of gSES, we define a locus is unique to that trait if that locus does not overlap with gSES.

### Mendelian randomisation

For two-sample MR, UK Biobank data was divided into two non-overlapping subsets, one for the exposure and one for the outcome (Fig. 1). Genome-wide association study (GWAS) were performed to identify instrumental variables for six exposures. These were occupational prestige, household income, educational attainment, and social deprivation, and cognitive ability. A multi-variate GWAS was conducted on occupational prestige, household income, educational attainment, and social deprivation to extract a common SES factor (gSES), resulted into the sixth exposure. GWASs were also performed in an independent sample on thirteen MRI outcomes (total brain volume, TBV; grey matter volume, GM; normal appearing white matter volume, NAWM; white matter hyperintensity volume, WMH; TBV as a proportion of intracranial volume, TBVicv; GM as a proportion of intracranial volume, GMicv; white matter volume as a proportion of intracranial volume, WMicv; WMH as a proportion of intracranial volume, WMHicv; a general factor of brain white matter tract fractional anisotropy, gFA; a general factor of brain white matter tract mean diffusivity, gMD; a general factor of brain white matter tract intracellular volume fraction, gIVCF; a general factor of brain white matter tract isotropic volume fraction, gISOVF; a general factor of brain white matter tract orientation dispersion, gOD) capturing different aspects of brain morphology. Publicly available non-UK biobank GWAS data were downloaded to replicate MR findings. More details see Online Methodology.

A valid inference from MR is dependent on satisfying three assumptions: relevance, meaning that the genetic variants must be associated with the risk factor of interest; independence, that the there are no unmeasured confounds of the associations between genetic variants and the outcome; exclusion restriction, that the genetic variants affect the outcome only through the effect they have on the exposure [39].

Instruments for each exposure were identified using SNPs that attained genome-wide significance (*P* < 5 × 10^−8^). These SNPs were then clumped using the 1000 G European reference panel and an r^2^ = 0.001, with a 10 Mb boundary. The most significant SNP in each clump was used as an instrumental variable. As all GWAS conducted for this study were performed on the same strand, no palindromic SNPs were excluded from these analyses. The effect of each SNP on the exposure and on the outcome was harmonised to ensure that the effect allele is the same across the exposure and the outcome traits. Steiger filtering was used to ensure that the detected direction of effect (i.e., from exposure to outcome) was correct.

Inverse variance weighted (IVW) regression was used to identify putatively causal effects. If there is only one SNP to be used as an instrumental variable, Wald ratio was used. Sensitivity analyses were conducted using MR Egger regression and MR Pleiotropy Residual Sum and Outlier (MR-PRESSO).

As cognitive ability shows high genetic correlations with measures of educational attainment [21] and shows potential causal effects on income [8]. We applied Multivariable Mendelian Randomisation (MVMR) [40] to examine the direct effects of SES independent of cognitive ability on brain structure. For MVMR, SNPs that were genome-wide significant in both exposures were retained. Steiger filtering was applied for both exposures on the outcome.

To correct for multiple testing, we performed FDR correction for IVW method for each of the following families. These are gSES as exposure on 13 brain MRI phenotypes as outcome (1 × 13 = 13 tests), 13 brain MRI phenotypes as exposure on gSES as outcome (13 × 1 = 13 tests), occupational prestige, household income, and educational attainment as exposures on 13 brain MRI phenotypes as outcome (3 × 13 = 39 tests), 12 brain MRI phenotypes as exposure on occupational prestige, household income, educational attainment, social deprivation as outcomes (12 × 4 = 48 tests), cognitive ability as exposure on gSES, occupational prestige, household income, educational attainment, social deprivation as outcome (1 × 5 = 5 tests), and gSES and occupational prestige, household income, and educational attainment as exposure on cognitive ability as outcome (4 × 1 = 4 tests). Significant threshold was set to FDR < 5%. Note, as lack of IVs, social deprivation and NAWM were not used as exposure in our study.

### Replication data sets

Replication of significant MR associations was examined using independent GWAS data set of educational attainment (measured as the number of years of schooling an individual has completed) [9] (*N* = 324,162) and household income [41].

Household income was replicated using the data of Kweon, Burik [41] excluding UKB. Four income measurements (measured as the natural log of income before-tax) were used: household income *N* = 108,635, occupational income *N* = 149,997, individual income *N* = 72,235, and parental income *N* = 105,667. Household income was meta-analysed with the other three income measurements using MTAG, resulting in a final household income replication GWAS dataset with an effective sample size of 402,029.

The replication data set for education showed a large significant genetic correlation of *r*_*g*_ = 0.960, SE = 0.015, *P* < 1 × 10^−323^ with education in UK Biobank, as did the two household income data sets *r*_*g*_ = 0.955, SE = 0.028, *P* = 1.34 × 10^−251^.

### MiXeR

MiXeR v1.3 (https://github.com/precimed/mixer) was used to examine the genetic overlap between cognitive ability and gSES. First, a univariate model [42] was run to study the polygenicity (i.e. number of variants) of each trait using the Z-score from GWAS summary statistics and 1000 G European LD panel. Second, a bivariate model [43] was used to estimate the genetic overlap (i.e. number of variants shared between cognitive ability and gSES) using the parameters learned from the univariate model. The analysis was repeated twenty times using 2 million randomly selected SNPs at each time. The overlap between cognitive ability with occupational prestige, household income, educational attainment, and social deprivation was also performed. The results across twenty runs were then averaged and the genetic overlap of the best model with the lowest –log likelihood ratio was plotted.

## Results

### Phenotypic and genetic structure of SES

**Fig. 2:**
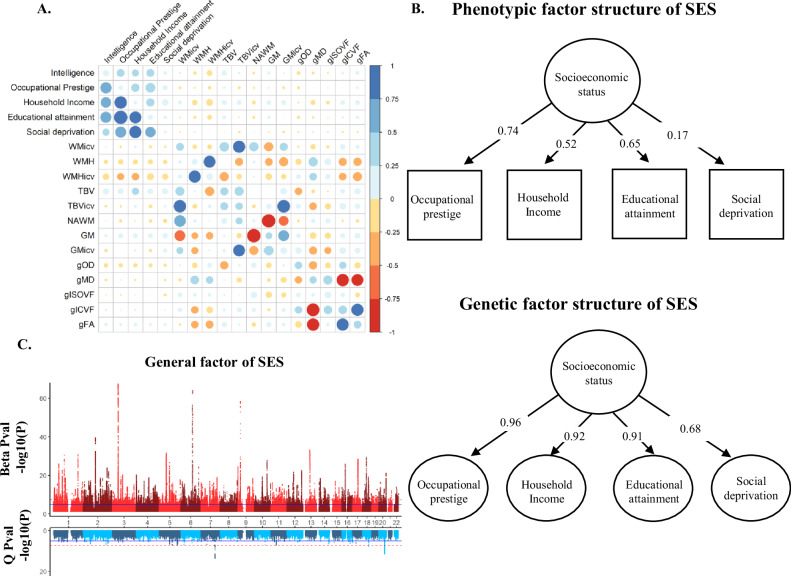
Genetic relationship between the indicators of SES and with MRI measures. **A** Showing the phenotypic and genetic correlations between the variables used in UK Biobank. The lower diagonal shows the genetic correlations whereas the upper diagonal shows the phenotypic correlations. The diagonal shows the heritability estimates. Colour and size are used to illustrate the magnitude and directions of the correlations. Both heritability and genetic correlations were derived using LDSC implemented in Genomic SEM. Tabulated values are shown in Supplementary Tables 1–3. Social deprivation scores were reversed to facilitate a comparison with the other measures of SES. **B** Showing the standardised phenotypic (upper UK Biobank) and genetic (lower total sample) factor solutions for the covariance structure across the four indices of SES examined. Social deprivation scores were again reversed. Squares represent observed variables (i.e. those that were directly measured) whereas circles represent latent variables (i.e. those that were statistically inferred). **C** A miami plot of the general factor of SES in the total sample (effective *N* = 947,466). The X axis indicates chromosome and the y axis shows the –log(10) *p* value of each SNP with the upper section describing its association with the general factor of SES where the lower shows the *p* value for the heterogeneity Q statistics. TBV total brain volume, GM grey matter volume, WMH white matter hyperintensity volume, TBVicv TBV as a proportion of intracranial volume, GMicv GM as a proportion of intracranial volume, WMicv white matter volume as a proportion of intracranial volume, WMHicv WMH as a proportion of intracranial volume, gFA a general factor of brain white matter tract fractional anisotropy, gMD a general factor of brain white matter tract mean diffusivity, gIVCF a general factor of brain white matter tract intracellular volume fraction, gISOVF a general factor of brain white matter tract isotropic volume fraction, NAWM normal appearing white matter, gOD a general factor of brain white matter tract orientation dispersion.

The phenotypic correlations between the occupational prestige, household income, educational attainment, and social deprivation (Supplementary Table 1) were all significant and ranged from *r* = 0.062–0.484 (mean = 0.268, SE range = 0.00143–0.00185, *P* < 10^−322^). A confirmatory factor model with a single common factor fit the phenotypic data poorly (χ^2^(2) = 8530.202, *P* < 0.001; SRMR = 0.047; CFI = 0.932; RMSEA = 0.131, TLI = 0.795, Fig. 2A & Table 1). The common factor explained 31.37% of the phenotypic variance across each of the four traits used to derive common phenotypic factor.

**Table 1:** Showing the standardised factor loadings for each of the four indicators of SES in the total sample (effective sample size *n* = 947,466).

Phenotypic SES	Factor loadings		Proportion of phenotypic variation			
Indicator of SES	Beta	SE	Common %	Specific %		
Occupational prestige	0.737	0.003	54.4	45.6		
Household Income	0.516	0.002	26.6	73.1		
Educational attainment	0.646	0.002	41.7	58.3		
Social deprivation	0.168	0.002	2.8	97.2		
Genetic SES	Factor loadings		Proportion of genetic variation			
Indicator of SES	Beta	SE	Common %	Specific %	h^2^%	SE
Occupational prestige	0.960	0.020	92.11	7.89	11.01	0.43
Household Income	0.920	0.020	84.69	15.31	4.94	0.22
Educational attainment	0.908	0.017	82.38	12.37	12.25	0.36
Social deprivation	0.681	0.022	46.37	53.63	3.43	0.17

The direction of social deprivation was reversed so that all scores indicate a greater level of SES across the four indicators used. The upper portion shows the phenotypic structure of SES where the bottom portion shows the genetic structure of SES. Common and specific, by definition sum to 100%, but for the genetic structure this indicates the proportion from common and specific sources that contribute to the total heritability. The total heritability was derived using LDSC implemented in genomic SEM.

Using LDSC [30] on each of the GWASs conducted on occupational prestige, household income, educational attainment, and social deprivation, a significant heritable component was captured explaining between 3.5–13% of trait variation (Supplementary Table 2). LDSC intercepts were consistently close to 1 for each trait indicating that polygenicity, rather than population stratification or other factors, explained the inflation in GWAS association test statistics (Supplementary Table 2).

Strong genetic correlations among these four traits (mean *r*_*g*_ = 0.761, range *r*_*g*_ = 0.563–0.963, SE range = 0.011–0.026) were observed (Supplementary Table 3). The moderate phenotypic correlations but large genetic correlations indicate that whilst each measure of SES captures a different environmental component, they each draw upon similar genetic components. This was confirmed by extracting a general genetic factor of SES (gSES) using genomic structural equation modelling (Genomic SEM [33], Fig. 2B & Table 1) where, in contrast to the phenotypic data, a single factor explained the covariance across the genetic data sets well (χ2(2) = 141.445, *P* = 1.93 × 10^−31^; SRMR = 0.038; CFI = 0.992). The general genetic factor of SES captured on average 76.39% of the genetic variance in each of the trait used to construct gSES with the proportion being consistent across occupational prestige, household income, and educational attainment (>80%), with the lowest being social deprivation where the general factor captured 46.37% (Supplementary Table 4). gSES was then regressed onto 7,462,121 SNPs to derive genome wide associations of gSES. gSES had a h^2^ = 9.40% (SE = 0.25%), and showed little evidence of inflation in test statistics due to population stratification (LDSC intercept = 1.07, SE = 0.01).

### Communality of effects on gSES

We use the heterogeneity (Q) statistics derived using our common factor model of socioeconomic status and the GWAS results of each used in its construction to examine if SNP effects act on a latent factor common to the traits used in its construction (Table 2). Such evidence would be consistent with the idea that a GWAS conducted on each trait used to construct gSES will capture similar underlying genetic architecture. FUMA [38] was used to derive independent genomic loci in gSES and the GWAS used in its construction. A total of 554 independent genomic loci were identified for the gSES (Fig. 2C), and of these 132 loci showed no overlap with any other traits indicating these loci act on the genetic architecture that is shared between each GWAS (Fig. 2C). Only 2 out of 554 loci showed evidence of heterogeneity. Occupational prestige, household income, educational attainment, and social deprivation were found to have 68, 112, 491, and 10 independent loci, respectively. However, only six loci for occupational prestige, 11 for household income and 100 associated with educational attainment, and four for social deprivation were independent from gSES further confirming that these SES traits share similar underlying genetic architecture.

**Table 2:** Showing a summary of the general factor of SES multivariate GWAS and the univariate GWAS used to derive gSES.

Trait	N	N significant loci (*P* < 5 × 10^−8^)	Independent of indicators of SES loci	Independent of Q loci	Mean χ^2^
Multivariate GWAS					
General factor of SES	947,466	554	132	552	2.90
	N	N significant loci (*P* < 5 × 10^−8^)	Independent of indicators of SES loci	Independent of general SES loci	Mean χ^2^
Heterogeneity (Q)	947,466	9	3	7	2.90
	N	N significant loci (*P* < 5 × 10^−8^)	Independent of general SES loci	Independent of Q loci	Mean χ^2^
Individual GWASs					
Occupational prestige	279,644	68	6	67	1.67
Household income	781,627	112	11	110	1.94
Educational attainment	753,152	491	100	486	2.77
Social deprivation	440,350	10	4	9	1.34

### Estimating causal effects of gSES on brain structure

In order to attain independent groups to perform Two-sample MR, all GWAS were re-run by omitting participants and their relatives who contributed MRI data. GWAS summary data sets provided by the Social Science Genetic Association Consortium (SSGAC) on educational attainment, and household income were also withheld to serve as independent samples for the replication of significant findings. This results in a sample size of 242,776 for occupational prestige, 327,402 for household income, 377,477 for educational attainment, and 382,030 for social deprivation, and an effective sample size of 665,662 for gSES. All data prepared for Two-sample MR had a highly similar factor structure and heritability as the full data set (Supplementary Table 4). Multiple testing correction was performed in the discovery via FDR.

Using Steiger filtering [44] followed by two-sample Mendelian randomisation (MR) [45] a higher gSES was found to be a protective factor against WMHicv at FDR < 5% (β = −0.218, SE = 0.056, *P* = 8.63 × 10^−5^, Table 3, Supplementary Table 5, Supplementary Figs 1–32). In sensitivity analysis, the use of both MR-Egger and MR-PRESSO did not identify any horizontal pleiotropy and no significant heterogeneity was found (Supplementary Table 5 & Supplementary Table 6). There was very little evidence of any effects of gSES on other traits.

**Table 3:** Showing the IVW bi-directional effect of gSES on brain structure and the IVW estimates of each trait used in the construction of gSES.

			IVW Causal effect estimate		
Exposure	Outcome	N SNPs	Beta	SE	P
	gFA	196	0.008	0.040	0.844
	gICVF	187	−0.012	0.039	0.763
	gISOVF	212	−0.018	0.037	0.615
	GM	184	391.502	752.483	0.603
	gMD	197	−0.020	0.037	0.583
	GMicv	190	344.811	1491.826	0.817
gSES	gOD	194	0.013	0.039	0.732
	TBV	177	3000.435	3624.226	0.408
	TBVicv	176	−1527.839	2553.570	0.550
	WMH	203	−0.073	0.039	0.060
	NAWM	234	−1377.250	1385.082	0.320
	WMicv	184	−1099.961	1618.823	0.497
	WMHicv	204	−0.218	0.056	8.63 × 10^−5^
Occupation	WMHicv	40	−0.012	0.006	0.041
Income	WMHicv	29	−0.376	0.111	0.001
Education	WMHicv	127	−0.593	0.128	3.77 × 10^−6^
Education replication	WMHicv	50	−0.186	0.083	0.026
gFA		34	0.012	0.014	0.407
gICVF		51	0.001	0.008	0.925
gISOVF		22	−0.012	0.022	0.575
GM		22	7.71 × 10^−7^	1.11 × 10^−6^	0.486
gMD		27	−0.004	0.014	0.767
GMicv		20	−5.22 × 10^−7^	4.57 × 10^−7^	0.253
gOD	gSES	23	−0.003	0.027	0.921
gICVF		51	0.001	0.008	0.925
TBVicv		28	−4.37 × 10^−7^	2.78 × 10^−7^	0.116
WMH		15	0.008	0.016	0.596
WMHicv		17	−0.033	0.021	0.117
WMicv		28	6.39 × 10^−7^	6.16 × 10^−7^	0.299
gFA		34	0.012	0.014	0.407
TBV		34	1.56 × 10^−6^	1.85 × 10^−7^	3.19 × 10^−^17
TBV	Occupation	34	2.15 × 10^−5^	2.97 × 10^−6^	4.57 × 10^−13^
TBV	Income	34	1.61 × 10^−6^	1.64 × 10^−7^	1.02 × 10^−22^
TBV	Income replication	14	1.22 × 10^−6^	2.33 × 10^−7^	1.65 × 10^−7^
TBV	Education	34	6.50 × 10^−7^	8.82 × 10^−8^	1.62 × 10^−13^
TBV	Education replication	25	1.31 × 10^−6^	2.68 × 10^−7^	9.86 × 10^−7^
TBV	Social deprivation	34	−1.84 × 10^−6^	3.34 × 10^−7^	3.41 × 10^−8^

*TBV* total brain volume, *GM* grey matter volume, *WMH* white matter hyperintensity volume, *TBVicv* TBV as a proportion of intracranial volume, *GMicv* GM as a proportion of intracranial volume, *WMicv* white matter volume as a proportion of intracranial volume, *WMHicv* WMH as a proportion of intracranial volume, *gFA* a general factor of white matter tract fractional anisotropy, *gMD* a general factor of white matter tract mean diffusivity, *gIVCF* a general factor of white matter tract intracellular volume fraction, *gISOVF* a general factor of white matter tract isotropic volume fraction, *gOD* a general factor of white matter tract orientation dispersion.

As expected from our finding that differences in WMHicv were a likely consequence of differences in gSES we found consistent evidence that differences in occupational prestige (β = −0.012, SE = 0.006, *P* = 0.041), household income (β = −0.376, SE = 0.111, *P* = 0.001), and educational attainment (β = −0.593, SE = 0.128, *P* = 3.77 × 10^−6^) were also a potential causal factor in WMHicv (Table 3, Supplementary Table 7, 8 & Supplementary Figs 4–12) where only occupational prestige did not withstand FDR correction for 36 tests. Only a few instrumental SNPs (<=3) were available for the MR analysis when using social deprivation as the exposure. Due to this lack of power these results are not presented here. These full results examining occupational prestige, household income, social deprivations, and educational attainment on brain structure are presented in Supplementary Table 7 & 8.

The effect of educational attainment on WMHicv was replicated using an independent sample of *N* = 324,162 (β = −0.186, SE = 0.083, *P* = 0.026, Supplementary Table 9 & 10).

### Estimating causal effects of brain structure on gSES

n the reverse direction a greater total brain volume (TBV) was associated with higher gSES at FDR < 5% (β = 1.56 × 10^−6^, SE = 1.85 × 10^−7^, *P* = 3.19 × 10^−17^, Table 3, Supplementary Table 5 & Supplementary Figs 13–15. No horizontal pleiotropy was detected using MR-Egger or MR-PRESSO but significant heterogeneity was found (Supplementary Table 5 & Supplementary Table 6). None of the other structural brain measures showed evidence of being a causal factor in differences in gSES.

The same effects were consistently observed for occupational prestige, household income, educational attainment, and social deprivation and were replicated in independent cohorts. In UKB, there was evidence of TBV likely contributing to: occupational prestige (β = 2.15 × 10^−5^, *P* = 4.57 × 10^−13^), household income (β = 1.67 × 10^−6^, *P* = 1.02 × 10^−22^), educational attainment (β = 6.50 × 10^−7^, *P* = 1.62 × 10^−13^), and social deprivation (β = −1.84 × 10^−7^, *P* = 3.41 × 10^−8^, Table 3 & Supplementary Table 7). All effects remained significant after multiple testing correction at FDR < 5%. MR-Egger regression indicated little evidence of horizontal pleiotropy as the MR-Egger intercept was indistinguishable from zero in each comparison (Supplementary Table 7) and MR-PRESSO did not detect any outliers influencing the estimate through horizontal pleiotropy (Supplementary Table 8 & Supplementary Figs. 16–22).

The effects of TBV on both educational attainment (β = 1.01 × 10^−6^, *P* = 6.14 × 10^−6^) and household income (β = 1.22 × 10^−6^, *P* = 1.65 × 10^−7^) were replicated in two independent samples (Supplementary Table 9 & 10).

No other significant effects of brain structure on gSES, occupational prestige, household income, educational attainment, or social deprivation were discovered at FDR < 5%.

### The role of cognitive ability in the link between SES and brain structure

**Fig. 3:**
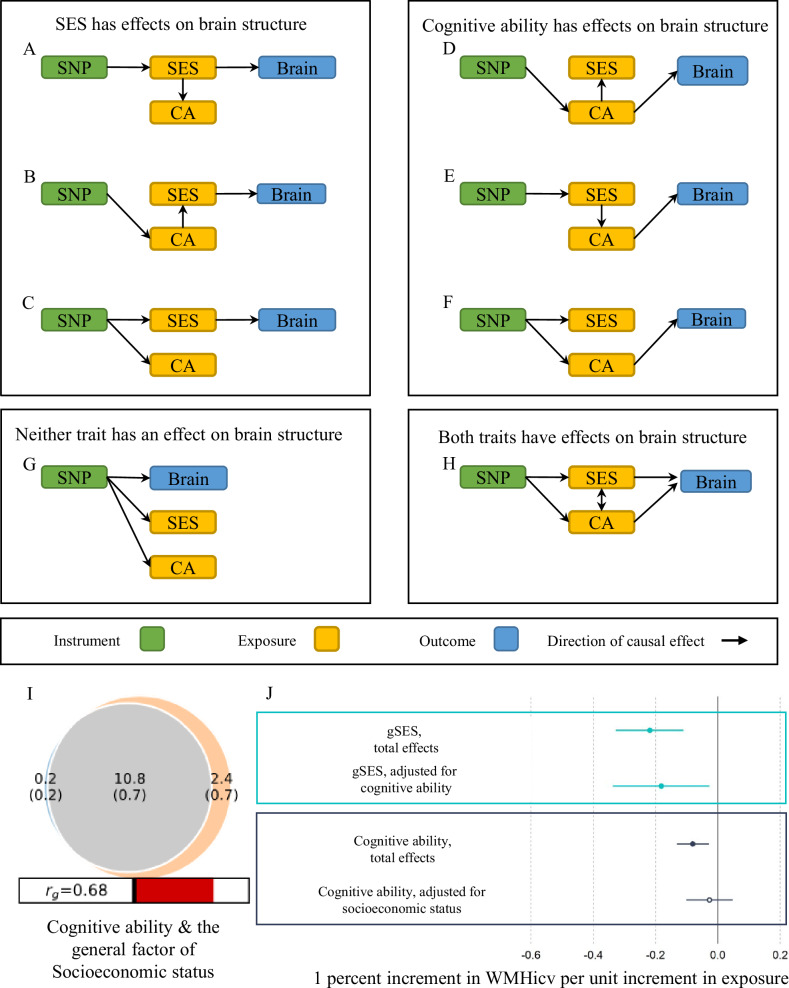
Genetic relationship between SES, cognitive ability and white matter hyperintensities. A–G shows a selection of models that may underlie the univariable MR effects of gSES and cognitive ability on brain structure. SES indicates gSES, CA indicates cognitive ability, and brain indicates brain structure (WMHicv). SNP indicates a set of SNPs used to derive instrumental variables illustrated with a single box for ease of plotting. A–C indicates models where SES has an effect on brain structure. A shows that the univariate effects of CA are due to confounding. B shows a model whereby the effects of SES on brain structure are mediated through CA, and C shows a model where the effect of CA on brain structure is best explained by confounding due to horizontal pleiotropy between SES and CA. Models D–F show the same pattern of effects but with CA, not SES, as the likely causal variable. Model G shows a scenario where neither CA nor SES has an effect on brain structure but SNP influences brain structure through a separate path. H shows a model where both CA and SES have independent effects on brain structure. I shows a Venn diagram of cognitive ability and gSES showing the unique and shared genetic components at the causal level. Grey illustrates the polygenic overlap between trait pairs, orange shows the SES specific components, and blue the unique contributors to cognitive ability. Numbers indicate the estimated quantity of causal variants in thousands with the standard error in brackets. The size of the circle indicates the degree of polygenicity for each trait pair. J Illustrating the total and direct effects of gSES, and cognitive ability. Colour represents trait and solid shapes indicate a statistically significant causal estimate. Error bars show ± one standard error.

Cognitive ability has strong genetic [21] and phenotypic correlations with the traits used in the construction of gSES (Fig. 2A). Furthermore, cognitive ability, has previously been shown to have a likely causal effect on income and educational attainment [8, 12, 22]. Figure 3A–H illustrates several possible underlying models that may explain the observed associations between cognitive ability, SES, and brain structure. Whilst these are by no means exhaustive, they are facilitative in the identification of potential intervention targets. In order to help differentiate between our models of the role cognitive ability plays in the relationship between gSES and WMHicv we first detail the genetic overlap between the gSES and cognitive ability using MiXeR. Then we use MVMR to estimate the direct effects (i.e. those independent of cognitive ability) of gSES on WMHicv.

### Overlap of causal loci for cognitive ability and SES

MiXeR is a tool for estimating the total number of causal loci for a quantitative trait and how many of these loci are overlap with another quantitative trait. In contrast to LDSC, MiXeR can identify instances where two traits show a genetic overlap, but there are discordant SNP effects between the two traits. Using MiXeR [43], we examined the degree of polygenic overlap between cognitive ability with gSES. We estimate that 10.8 K (SE = 0.7 K) causal loci are shared between cognitive ability and gSES with 2.4 K (SE = 0.7 K) being unique to gSES. Of note is the absence of loci that were implicated as being causal of cognitive ability (0.2 K, SE = 0.2 K) and not shared with gSES (Fig. 3A). Similar patterns were observed for the traits used in the construction of gSES (Supplementary Fig. 23).

### Estimating the bidirectional effects between cognitive ability and gSES

Following Steiger filtering, we find evidence that higher cognitive ability was linked to having a higher level of gSES at FDR < 5% (β = 0.197, SE = 0.013, *P* = 4.79 × 10^−54^). The same effects were observed in all four traits used in the construction of gSES in UKB and were also replicated in independent samples provided by the SSGAC. In the UKB, cognitive ability was found to be linked with a higher level of occupational prestige (β = 2.67, SE = 0.15, *P* = 2.21 × 10^−69^), a greater level of household income (β = 0.131, SE = 0.011, *P* = 2.66 × 10^−31^), a greater chance of attaining a university level education (β = 0.077, SE = 0.004, *P* = 5.75 × 10^−67^), and decrease in level of deprivation in which one lives (β = −0.84, SE = 0.025, *P* = 0.001, Table 4 & Supplementary Table 11). All effects remained significant after multiple testing correction at FDR < 5%. In the replication samples provided by the SSGAC, cognitive ability had an effect of (β = 0.123, SE = 0.012, *P* = 8.53 × 10^−25^) on educational attainment and of (β = 0.078, SE = 0.017, *P* = 3.32 × 10^−6^) on household income. There was evidence of heterogeneity in each estimate of the effects of cognitive ability on SES as indicated by significant Cochran’s Q statistics [46] (Supplementary Table 11). This heterogeneity statistic provides an indication of the variability of the estimated effect between SNPs and can arise if the SNPs have horizontal pleiotropic effects. However, there was little evidence that horizontal pleiotropy biased the estimated effect of cognitive ability on gSES or on the four traits used in the construction of gSES; the MR Egger regression intercepts were close to zero and MR PRESSO indicated no significant distortion in the estimate due to SNPs with a horizontal pleiotropic effect (Supplementary Table 12, & Supplementary Figs. 24–31).

**Table 4:** Showing the bi-directional total causal effects of cognitive ability on the general factor of SES and each of the four indicators of SES.

			IVW Causal effect estimate		
Exposure	Outcome	nSNPs	Beta	SE	P
	gSES	78	0.197	0.01	4.79 × 10^−53^
	Occupation	72	2.665	0.151	2.21 × 10^−69^
Cognitive ability	Income	75	0.131	0.011	2.66 × 10^−31^
	Income (replication)	46	0.089	0.015	1.15 × 10^−15^
	Education	65	0.077	0.004	5.75 × 10^−67^
	Education (replication)	58	0.123	0.012	8.53 × 10^−25^
	Social deprivation	79	−0.084	0.025	0.001
gSES		170	1.206	0.044	2.39 × 10^−164^
Occupation		37	0.073	0.007	2.97 × 10^−27^
Income	Cognitive ability	27	1.175	0.120	9.51 × 10^−23^
Education		127	2.678	0.133	5.46 × 10^−90^
Education (replication)		44	0.870	0.097	2.87 × 10^−19^

Beta weights are unstandardized and reflect the original unit of measure.

In the backward analysis, we find evidence at FDR < 5% that increase in gSES and the traits used in its construction leads to increase in cognitive ability. These effects are gSES (β = 1.159, SE = 0.045, *P* = 1.19 × 10^−143^), education (β = 2.433, SE = 0.129, *P* = 7.54 × 10^−90^), income (β = 1.103, SE = 0. 116, *P* = 2.17 × 10^−21^), occupational prestige (β = 0.071, SE = 0.007, *P* = 7.93 × 10^−27^) and social deprivation (β = −0.415, SE = 0.121, *P* = 0.01, Supplementary Table 11). This effect was also replicated for educational attainment in the replication sample (β = 0.856, SE = 0.093, *P* = 4.43 × 10^−20^). As with the effects of cognitive ability on gSES there was significant heterogeneity in the estimates (Supplementary Table 11) but little evidence of bias arising due to horizontal pleiotropy indicated by the MR Egger intercepts not being significantly different from zero and no distortion detected using MR-PRESSO (Supplementary Table 12 & Supplementary Figs 32–41).

### The bidirectional causal effect of cognitive ability on brain structure

We find evidence that cognitive ability has a protective effect on WHMicv (β = −0.080, SE = 0.026, *P* = 0.002), and there was evidence to suggest a greater total brain volume resulted in a higher level of cognitive ability (β = 3.97 × 10^−6^, SE = 4.96 × 10^−7^, *P* = 1.28 × 10^−15^). No evidence of horizontal pleiotropy was identified using MR Egger (Egger_intercept_ *P* = 0.492) and MR-PRESSO found no evidence of distortion in the estimate following the removal of five SNPs with evidence of horizontal pleiotropy (MR-PRESSO distortion *P* value = 0.516). There was however, significant heterogeneity in the estimate of TBV on cognitive ability (Q *P*-value = 1.85 × 10^−10^, Supplementary Tables 13, 14 & Supplementary Figs 42–47).

### Direct effects of gSES on WMHicv conditioned on cognitive ability

Our previous findings indicate that the genetic variants associated with cognitive ability are nearly embedded within those of gSES and accounted for a substantial proportion of gSES (Fig. 3I). Additionally, cognitive ability appears to exert potential causal effects on both WMHicv and gSES (Table 4 & Supplementary Table 13). This raised the possibility that the observed effects of gSES on WMHicv may be the result of confounding by cognitive ability or/and pleiotropy (Fig. 3A–H). To clarify the role of cognitive ability on the link between gSES on WMHicv, we performed multivariable MR (MVMR) [40] to control for the effects of cognitive ability on WMHicv and assess the direct effects of gSES on WMHicv that were independent of cognitive ability. When both cognitive ability and gSES were included in a single multivariate model, there was evidence that the gSES effects on WMHicv were independent of cognitive ability (direct effect β = −0.182, SE = 0.079, *P* = 0.022) and the direct effect is similar to the total effect (β = −0.218, SE = 0.056, *P* = 8.63 × 10^−5^) estimated in the univariate MR analysis. However, there was no evidence of a direct effect of cognitive ability (direct effect β = −0.027, SE = 0.038, *P* = 0.480, Fig. 3J, Supplementary Table 15).

## Discussion

Those individuals from more advantaged socioeconomic backgrounds will typically have fewer instances of poor physical and mental health compared to those from more deprived backgrounds [1, 5–7]. Understanding the causes of such differences has the potential to decrease health disparities and improve our understanding of the intricate working of societal risk factors of illnesses. In the current study we examine the role that SES plays on brain structure by performing a multivariate GWAS to capture sources of SES differences that effect the individual, the household, and the area in which one lives. Our GWAS on gSES was then used to derive instrumental variables to examine the potential causal effect differences in SES has on brain morphology and health. The current study contributes to our understanding of the genetic architecture of SES in at least four ways.

First, we show that whilst a common phenotypic factor explains only 31.2% of phenotypic variation across occupational prestige, household income, educational attainment, and social deprivation, our multivariate general genetic factor of SES (gSES) accounted for on average 76% (range = 46–92%) of the genetic variation found across these traits. Furthermore, our common factor model showed little evidence of heterogenous effects for gSES where, of the 554 independent genomic loci identified, only two showed evidences of a heterogenous effect indicated by a significant Q value. This asymmetry in the variance captured by a common phenotypic factor of SES compared with the variance captured by a common genetic factor of SES, and the finding that the majority of loci associated with the general factor acted on each trait used to construct gSES, implies that although each trait captures a different environmental component of SES, the underlying genetic architecture of these SES related traits that give rise to these phenotypic differences are largely the same.

The identification of homogenous genetic architecture among all SES measures allows for the recontextualisation of the results of previous GWAS that have been conducted on individual indicators of SES. Specifically, many of the loci identified in univariate GWAS of a single indicator of SES are generalisable to SES more broadly, as they are associated with all indicators that load on the general genetic factor of SES. For example, previous GWAS examining educational attainment [20] and income [8] have reported 3952 and 149 loci respectively as showing an association with a specific indicator of SES. Here, we find that 82.38% of the genetic variance of educational attainment and 84.69% of the genetic variance of income is through this general factor of SES indicating that only a minority of the loci captured by those GWAS on specific indicators of SES will be trait specific.

Second, we find evidence that cognitive ability is highly relevant to and may have a causal effect on SES. By using MiXeR [43] we show that of the estimated 11,000 genetic variants for cognitive ability and 13,200 genetic variants for gSES, 10,800 are shared between cognitive ability and gSES with only 2400 causal variants for gSES not shared with cognitive ability. Whilst MiXeR cannot differentiate between vertical and horizontal pleiotropy [43], there was little evidence of loci associated with cognitive ability that were not also associated with differences in gSES consistent with the hypothesis that differences in cognitive ability may influence differences in SES.

By using two-sample MR we were able to confirm that vertical pleiotropy, and not horizontal pleiotropy, best explained the overlapping genetic architecture between cognitive ability and gSES identified using MiXeR. Higher cognitive ability was one of the causal elements of having a greater level of the general factor of SES, a higher occupational prestige and educational attainment, a higher household income, and living in a less deprived environment. This effect was replicated using educational attainment and household income data sets that included participants from outside the UK indicating these effects were not specific to the UK or to the participants of UK Biobank. These effects were bidirectional and differences in gSES were also shown to influence cognitive ability.

Third, using two-sample MR we show that higher levels of gSES is likely a consequence of a greater total brain volume and a likely causal factor in lower levels of white matter hyperintensities (WMHicv). White matter hyperintensities are white matter lesions that, on fluid attenuated inversion recovery (FLAIR) MRI scans, show a signal intensity that is brighter than surrounding white matter [47]. WMHs are associated with vascular risk and small vessel disease [48] and may indicate permeability in the blood brain barrier as well as axonal and myelin degeneration [49] Furthermore, increases in WMH volume are associated with cognitive decline and higher risk of Alzheimer’s disease, as well as with lower levels of cognitive ability [50].

In the context of non-clinical community-dwelling adults, WMH volume is also a frank marker of neurodegeneration, being of extremely low prevalence in young adulthood [51]. However, lower levels of cognitive ability at age 11 are associated with greater WMH volume at age 73 [52] indicating that they may influence the trajectory of cognitive decline in adulthood and older age. Our finding that gSES was a likely causal factor for WMHicv indicates that lower levels of gSES may act as a risk factor for the development of WMH across the adult lifespan and may, through the accumulation of damage caused by WMH, increase the rate of cognitive decline and the likelihood of a dementia diagnosis in older age. In contrast, our finding that TBV was likely a causal factor for gSES and cognitive ability may indicate that TBV (which reaches its peak in early adulthood [53]) is a risk factor that influences the rate of cognitive development in childhood.

Fourth, we show using MVMR, that there was evidence of direct effects of gSES on WMHicv independent of cognitive ability. Using MVMR we were able to remove the effect of one of cognitive ability, in order to gauge the effect of gSES on brain morphology free from the effects of cognitive ability. In doing so we show that the direct effects of gSES are protective against WMHicv. The results of MVR provide support for the models A, B, and C from Fig. 3 showing the independent effect of SES on brain structure. However, previous studies examining the role of cognitive ability across the generations indicates that childhood cognitive ability is associated with upward and downward change from the SES of the parent [54, 55] making it unlikely that our results are best explained by model A. We can also discount model C as a potential explanation of our findings as it posits that the univariable MR finding of cognitive ability on WMHicv is the result of horizontal pleiotropy, however we find no evidence of horizontal pleiotropy in our estimates of cognitive ability on WMHicv. Rather, in our data, both our MVMR and MiXeR results are consistent with model B of Fig. 3 where the effects of cognitive ability on brain structure are mediated by SES, but as some loci are not shared between gSES and cognitive ability other traits also likely contribute to differences in SES [8, 56].

Given the large degree of communality between occupation, household income, educational attainment and social deprivation indicated by the general factor of SES, policymakers may consider the implementation of programmes aimed at acting on any combination of these indicators of SES. However, one should also consider the effect of cognitive ability in the causal pathway linking differences in SES to white matter hyperintensities. Cognitive ability is closely associated with educational attainment, household income, and occupational status and has been shown to be a likely causal factor in both education and income [8, 12]. Importantly, education has also been shown to be a likely causal factor in differences in cognitive ability [22, 57] and is a more seemingly more tractable target for environmental intervention than cognitive ability. Future research should thus explore strategies aimed at facilitating individuals’ participation in education, which may lead to better brain health in older age.

Our study has limitations that should be considered when interpreting the results. First, all samples used were from western European societies and cultures of the 21^st^ century. The importance of this caveat is underscored by the genetic architecture of SES are unlikely to be universal and will be specific to the populations and generations [58, 59]. Without studies aiming to examine to the difference in genetic architecture of SES and the role these play in brain structure in other samples, meaningful comparisons between the present study and other population are unwarranted.

Second, genetic variants captured by our GWAS on gSES (and on occupational prestige, household income, educational attainment and social deprivation) are likely to have pleiotropic effects [34] with lifestyle differences, disease traits, as well as exposure to certain environmental stressors. To satisfy the assumptions that the genetic association with the outcome is entirely mediated via the exposure, we performed Steiger filtering to remove variants that are more strongly associated with outcome than the exposure (i.e. reverse causation, Non-Steiger filtered results are provided in Supplementary Tables 16–20). Although removing invalid instrumental variables and only keeping likely vertical pleotropic instrumental variables can improve the validity of causal effects, such data-driven selection of instrumental variables may yield over precise causal effects, especially when the majority of instrumental variables are affected by heterogeneity. Furthermore, in order to break the assumptions of MR it is not sufficient for the genetic variants in the instrumental variable to have pleiotropic effects [60]; rather the genetic variants must have horizontally pleiotropic effects that are mediated through mechanisms other than those captured by SES. For example, should genetic variants have vertically pleiotropic effects, e.g. SNP->neuron-> cognitive ability ->gSES-> lifestyle, comorbidities, or environmental influences ->brain structure, then our MR derived causal estimates will not be biased. Furthermore, should the SNPs affect other phenotypes, but these phenotypes do not affect the outcomes, then our MR estimates will not be biased. Whilst it is possible that the genetic variants identified in our GWAS conducted on gSES do have horizontally pleiotropic effects, it is unclear what mechanisms would mediate such effects. In the current study we investigate potentially pleiotropic effects using multivariable Mendelian randomization to examine the role of cognitive ability. Future research should use multivariable Mendelian randomization to investigate the role of other traits that link SES to brain structure.

Third, there is the potential that indirect genetic effects will contribute to the MR estimates [61]. Indirect genetic effects refer to one individual’s genotype influencing the outcome of another individual’s phenotype, for example, a parent providing material resources for their offspring which may affect SES or cognitive ability. Detecting the magnitude of potential bias resulting from dynastic effects is challenging outside of using family-based data, and at present no such data exist.

Finally, molecular genetic studies examining traits such as cognitive ability and socioeconomic status are prone to misunderstanding and mischaracterisation. These mischaracterisations can include arguments based around genetic determinism where the role of the environment is disregarded in favour of creating myths about immutable, biological differences underlying trait variation, something incompatible with current knowledge of complex traits. In order to communicate our research findings to a general reader in an ethical and socially responsible way, we have provided an FAQ document in Supplementary Note 1 and an overview of the study in Box 1 detailing how, and how not, these findings should be interpreted.

Overall, this study offers new insights into the complex interactions between socioeconomic status (SES), brain development and the risk factors underlying cognitive decline. Employing modern analytical methods on extensive datasets, the findings significantly contribute to our comprehension of factors that influence physical and mental health. Ultimately, these results highlight SES as a potentially modifiable risk factor, causal in the maintenance of cognitive ability in older-age.

## Supplementary information

Supplementary tables

Supplementary Figures

Frequently Asked Questions (FAQs) regarding: “Deciphering the influence of socioeconomic status on brain structure: insights from Mendelian randomization”

## Data Availability

Summary statistics GWASs for the structural MRI measures, the general factor of socioeconomic status (*n* = 947,466), social deprivation (*n* = 440,350), occupational prestige (*n* = 279,644), and the discovery GWAS data set for household income (*n* = 781,627), and educational attainment (*n* = 753,152) will be available on GWAS catalog upon publication (https://www.ebi.ac.uk/gwas/) under accession codes GCST90566682, GCST90566683, GCST90566684, GCST90566685, GCST90566686, GCST90566687, GCST90566688, GCST90566689, GCST90566690, GCST90566691, GCST90566692, GCST90566693, GCST90566694, GCST90566695, GCST90566696, GCST90566697, GCST90566698, GCST90566699, GCST90566700, GCST90566701, GCST90566702, GCST90566703, GCST90566704, GCST90566705, GCST90566706, GCST90566707, and GCST9056670. Also note that the summary data for the general factor of SES and each indicator of SES, excluding those who provided structural brain imaging measures and their relatives, are also available through GWAS catalog. We also make available the summary data of each individual indicator of SES meta-analysed using MTAG (occupational prestige, *n* = 683,663, household income, *n* = 1,485,217, educational attainment *n* = 848,919, and social deprivation, *n* = 862,391). The replication samples are available on request from the Social Science Genetic Association Consortium (https://www.thessgac.org/).

## Code availability

Regeine v3.1.3 (https://rgcgithub.github.io/regenie/) was used to perform GWAS with METAL (https://genome.sph.umich.edu/wiki/METAL_Documentation) being used to perform meta-analysis of educational attainment and household income traits. A general factor of SES was derived using Genomic SEM 0.0.5 C (https://github.com/GenomicSEM/GenomicSEM) and FUMA (version v1.3.6a) (https://fuma.ctglab.nl/) was used to identify trait associated loci. MiXeR v1.3 (https://github.com/precimed/mixer) was used to examine the loci shared between pairs of traits. Mendelian Randomisation was performed using TwoSampleMR 0.6.7 (https://mrcieu.github.io/TwoSampleMR/) implemented in R and the package “psych” version 2.4.1 (https://cran.r project.org/web/packages/psych/index.html). Brain structural and diffusion neuroimaging data were acquired, processed and QCd by the UK Biobank team as Imaging Derived Phenotypes (IDPs) according to open access publications references (Alfaro-Almagro, Jenkinson [23] & Miller, Alfaro-Almagro [24] of the main text) and online documentation (https://biobank.ctsu.ox.ac.uk/crystal/crystal/docs/brain_mri.pdf).
