# Supplementary material for: Deciphering the influence of socioeconomic status on brain structure: insights from Mendelian randomization
Source: Mol Psychiatry. 2025 May 13;30(10):4613–26. doi: 10.1038/s41380-025-03047-4 (PMC12436186; doi:10.1038/s41380-025-03047-4)
Supplement: Supplementary file 2 — Supplementary Figures [file 41380_2025_3047_MOESM2_ESM.docx]

## Socioeconomic status on brain structure


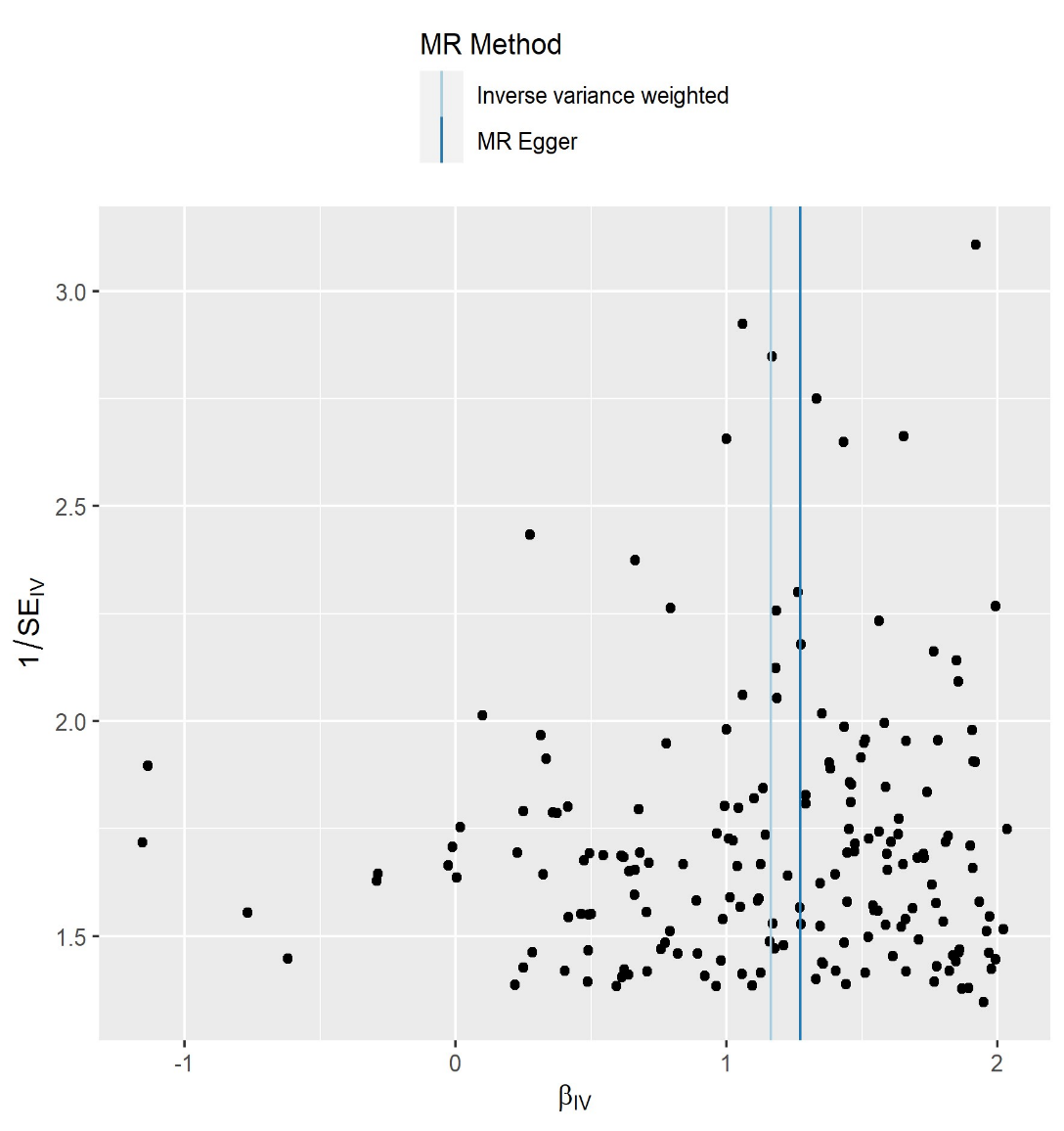

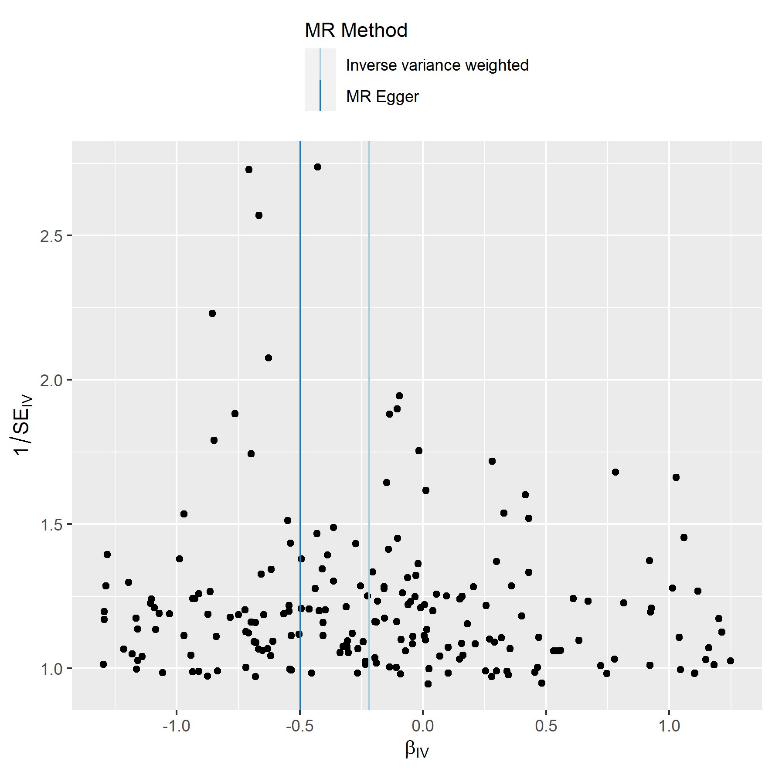


WMHicv

**Supplementary Figure 1.** Funnel plots used to examine the extent to which pleiotropy is balanced across the instruments used in the univariate Mendelian randomisation of general factor of socioeconomic status on white matter hyperintensities as a proportion of intracranial volume.


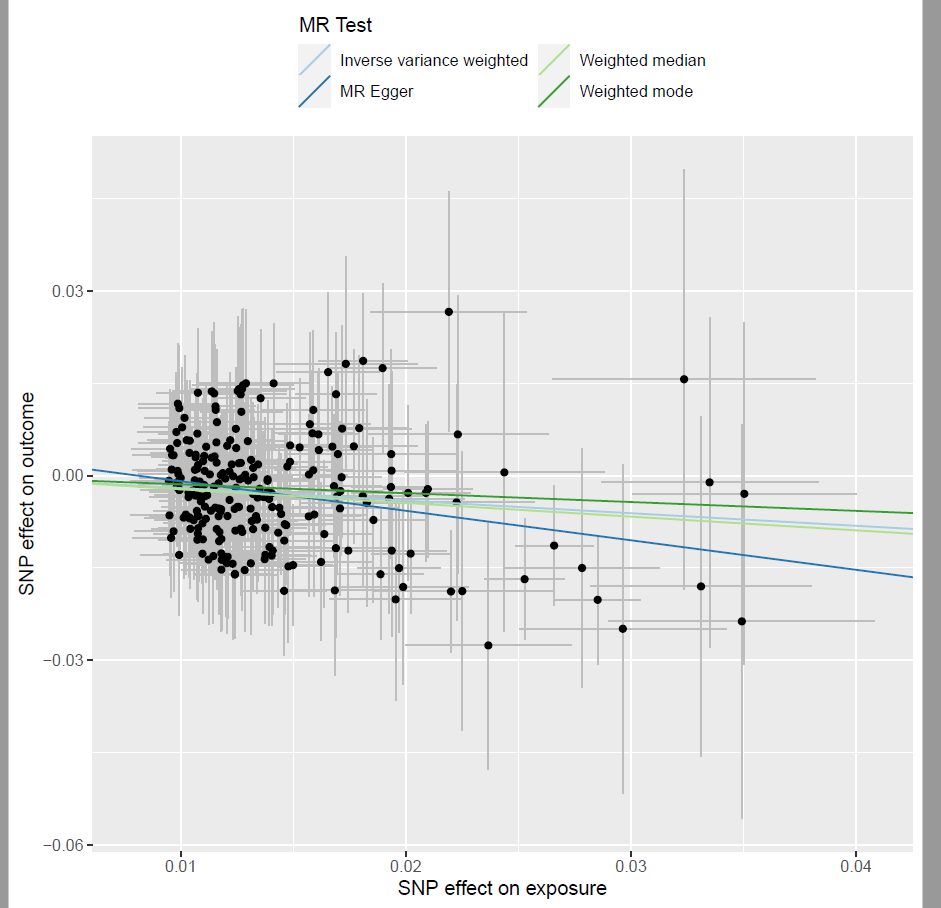

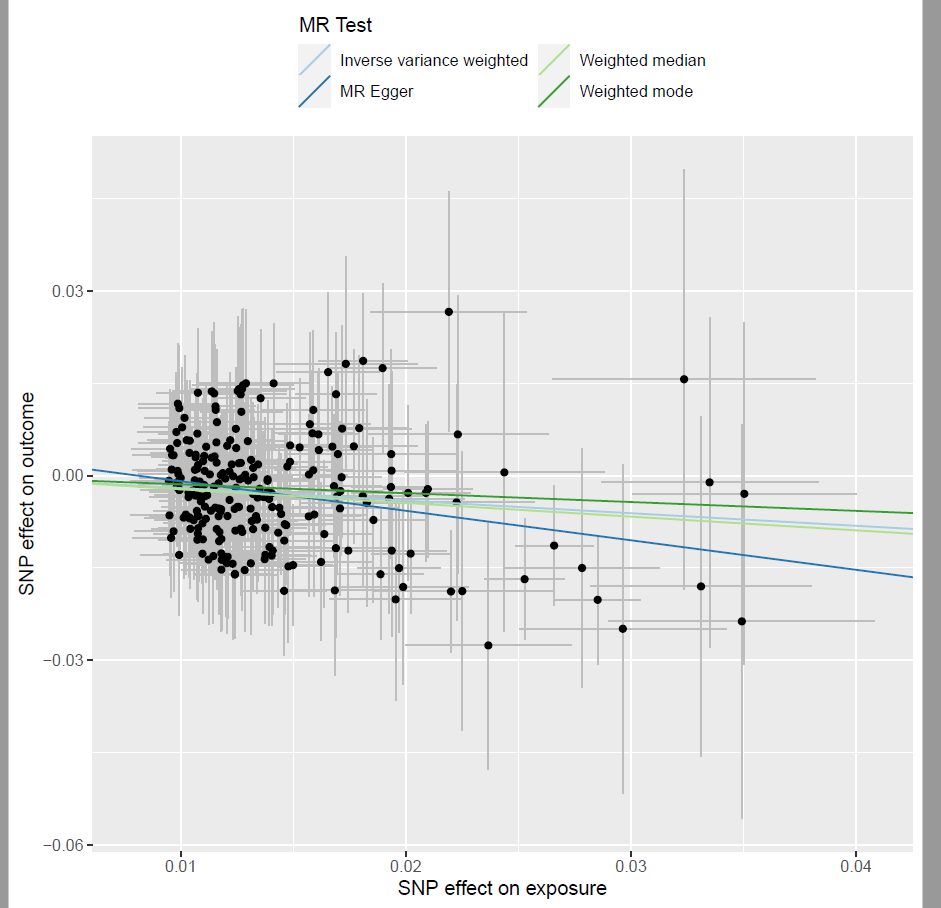


**SNP effect on outcome (WMHicv)**

**SNP effect on exposure (SES)**

**Supplementary Figure 2.** The effects of SES on WMHicv. The error bars indicate ± standard errors around the estimated phenotype-SNP associations.


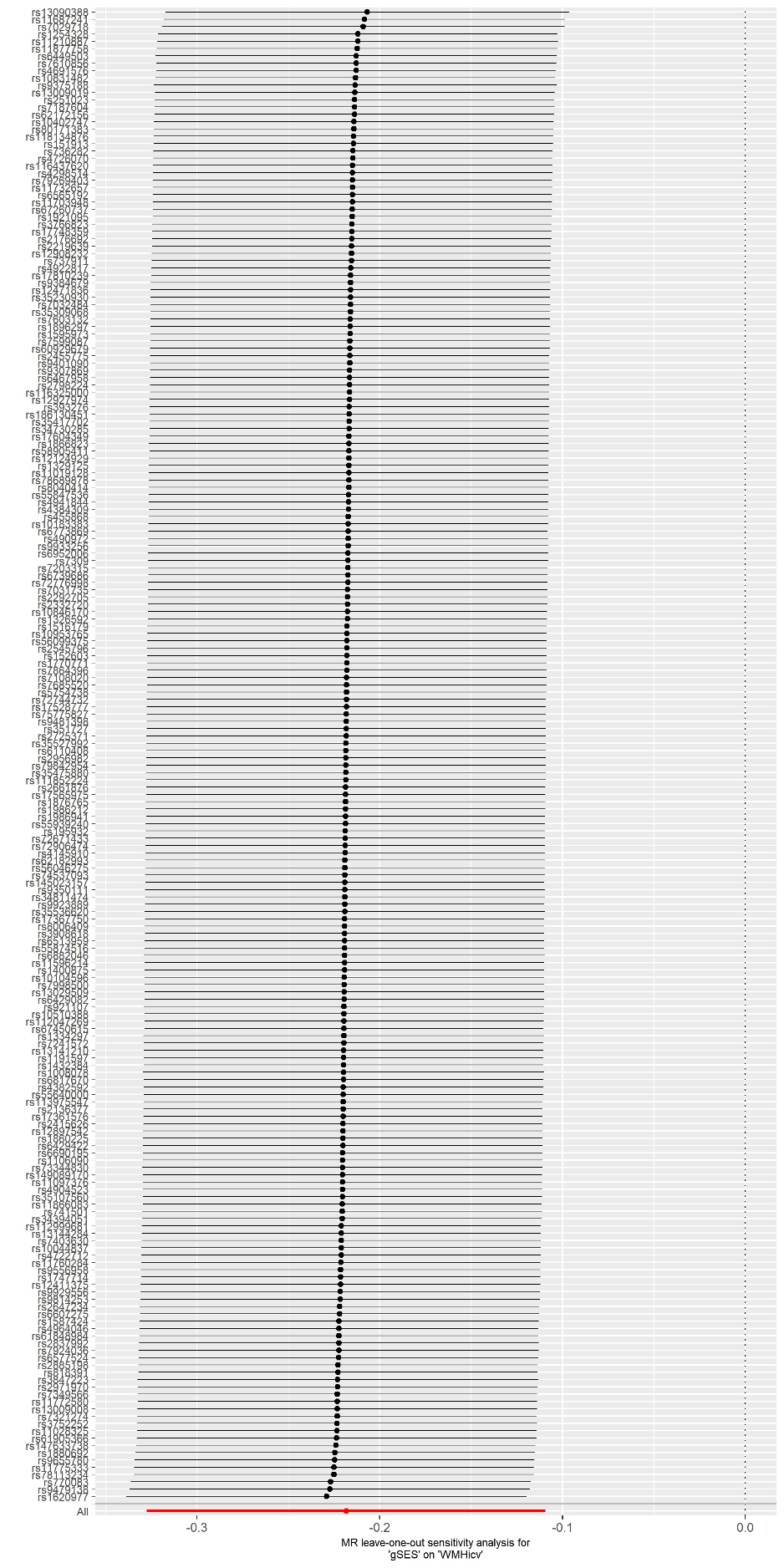


**Leave-one-out sensitivity analysis for SES on WMHicv**

**Supplementary Figure 3.** Leave-one-out plot of the univariate Mendelian randomisation of general factor of socioeconomic status on white matter hyperintensities as a proportion of intracranial volume.

## Household income on brain structure


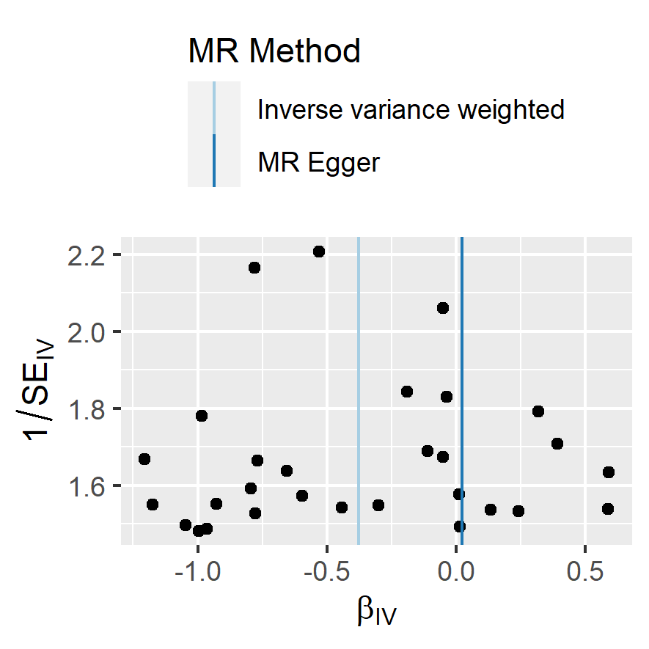


WMHicv

**Supplementary Figure 4.** Funnel plots used to examine the extent to which pleiotropy is balanced across the instruments used in the univariate Mendelian randomisation of Household income on brain structure.


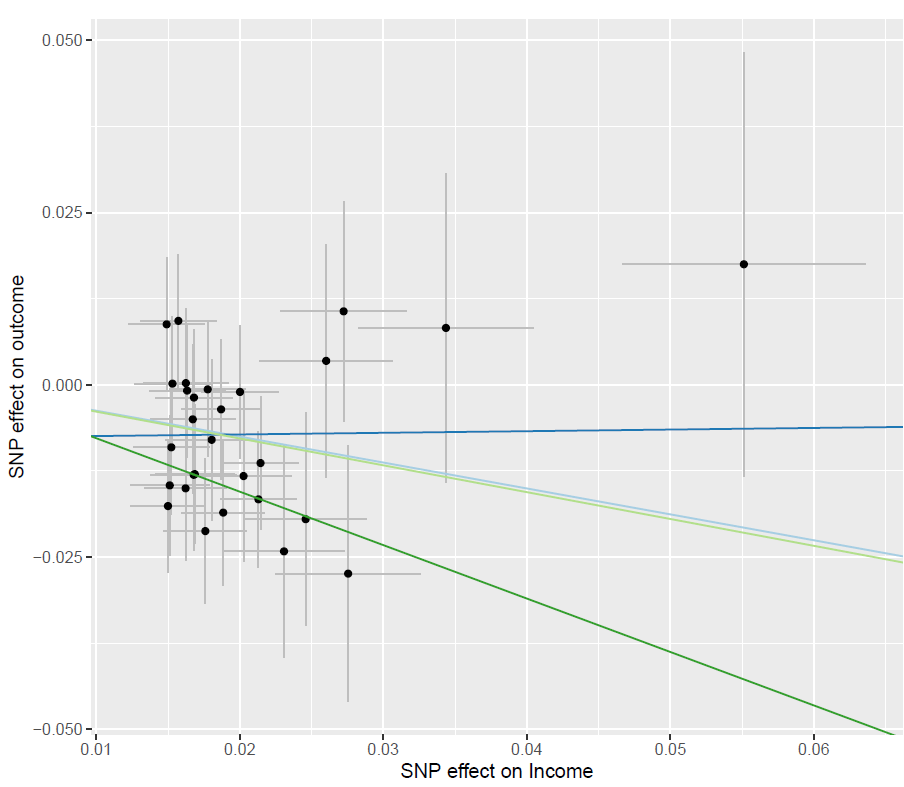

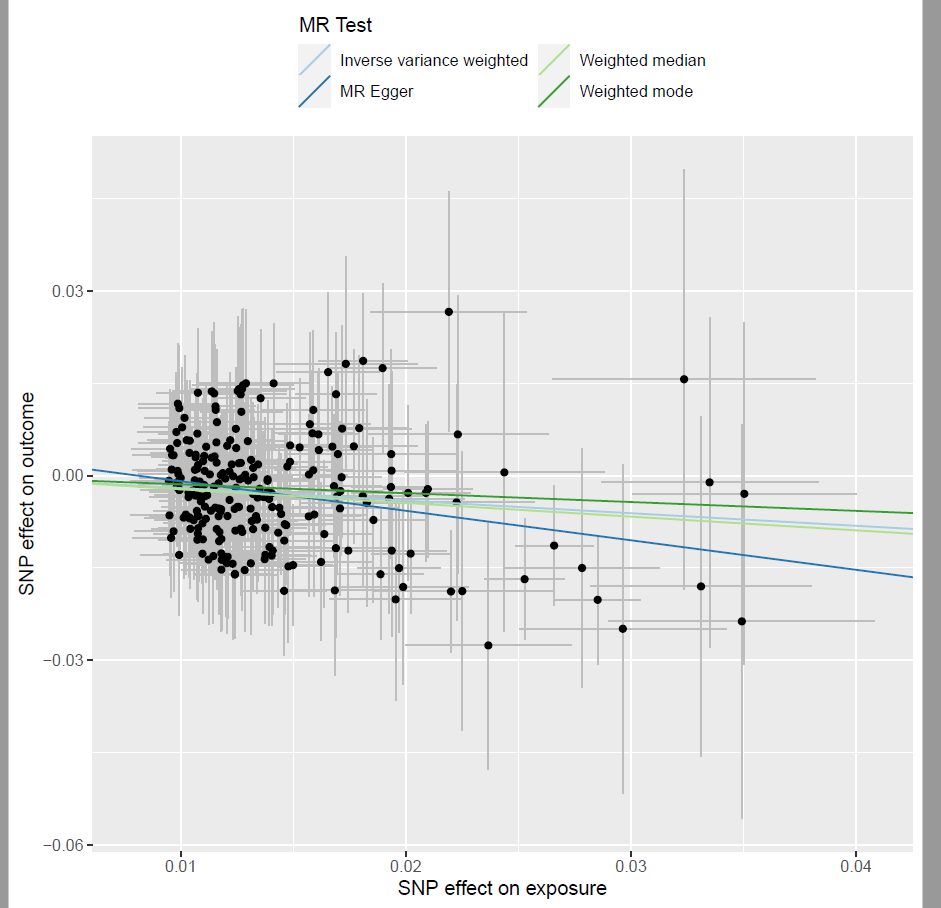


**SNP effect on exposure (Income)**

**SNP effect on outcome (WMHicv)**

**Supplementary Figure 5.** The effects of income on WMHicv. The error bars indicate ± standard errors around the estimated phenotype-SNP associations.


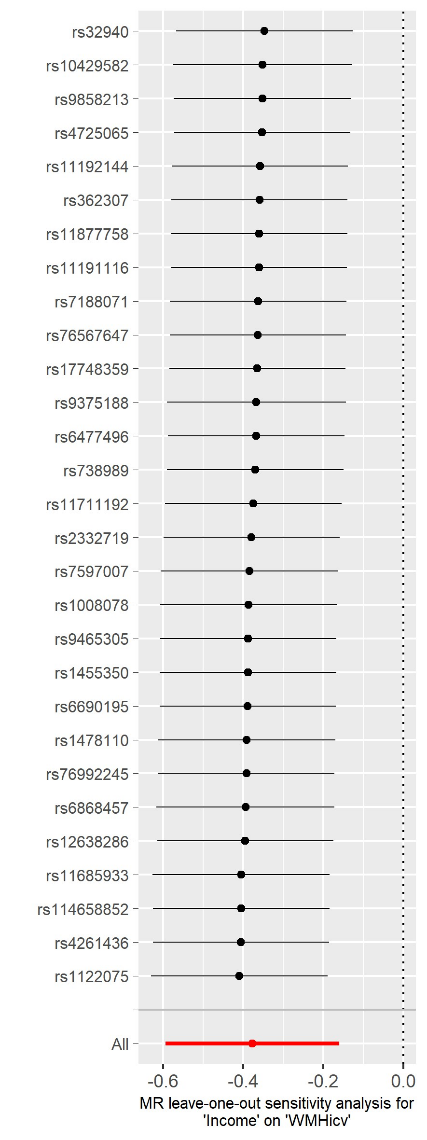


**Leave-one-out sensitivity analysis for Income on WMHicv**

**Supplementary Figure 6.** Leave-one-out plot of the univariate Mendelian randomisation of income, on white matter hyperintensities as a proportion of intracranial volume.

## Educational attainment on brain structure


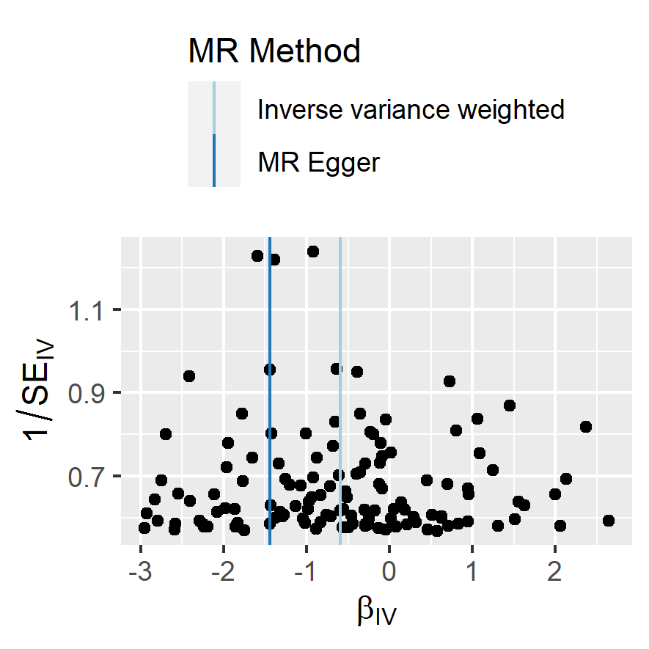


WMHicv

**Supplementary Figure 7.** Funnel plots used to examine the extent to which pleiotropy is balanced across the instruments used in the univariate Mendelian randomisation of Educational attainment on brain structure.


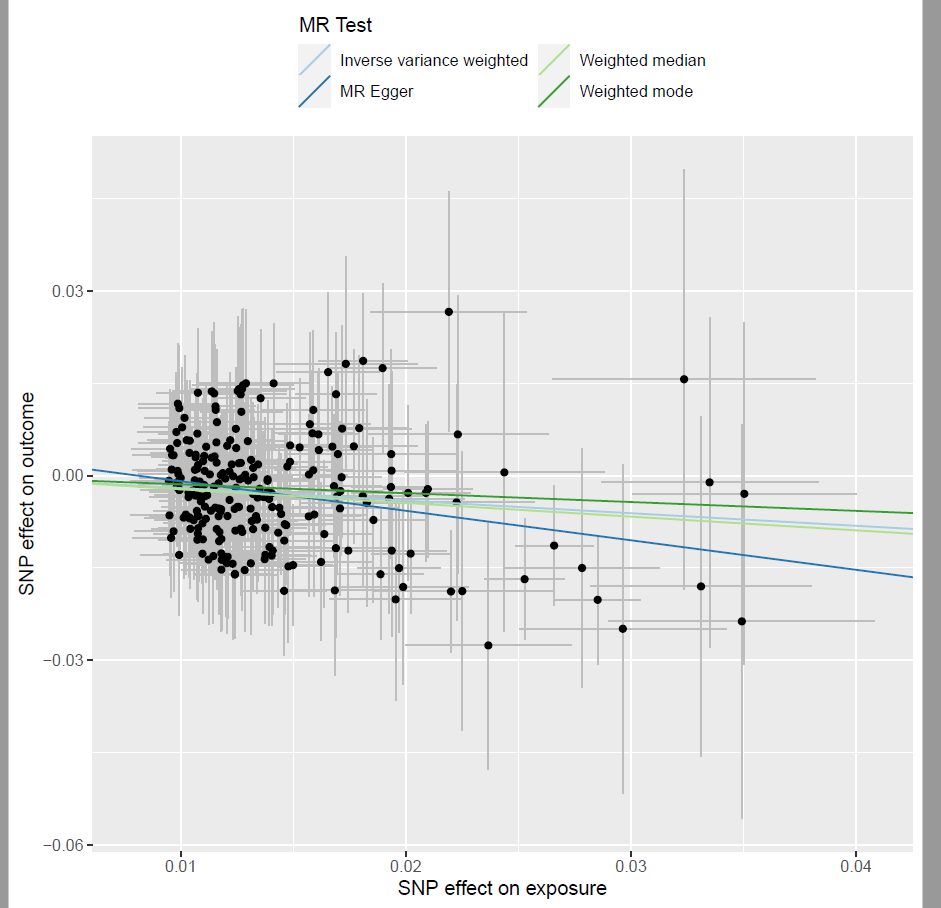

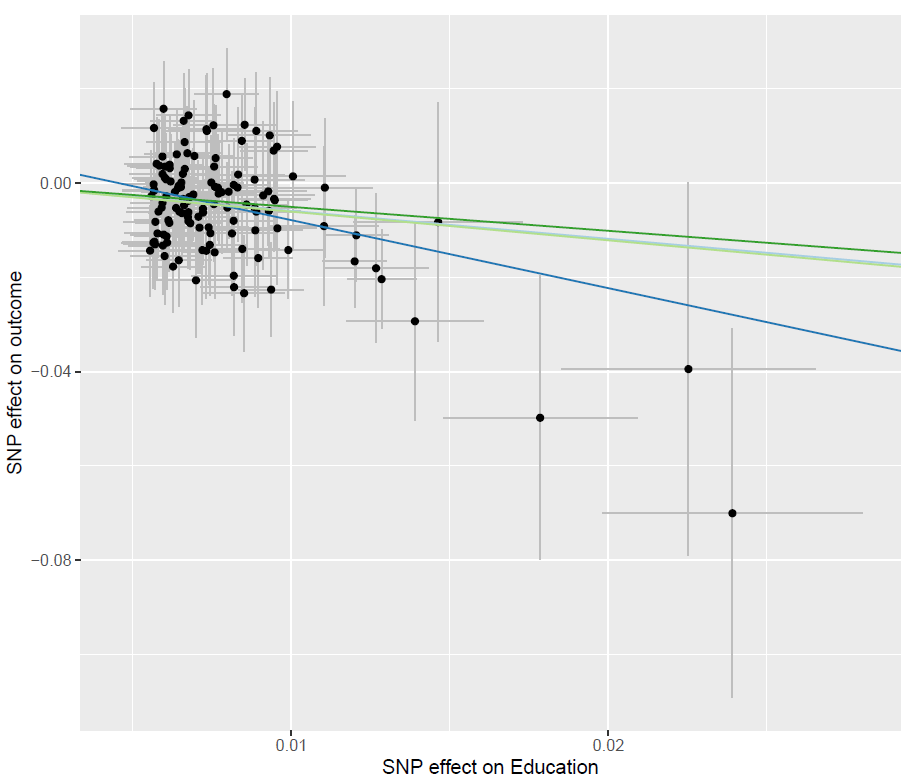


**SNP effect on exposure (Education)**

**SNP effect on outcome (WMHicv)**

**Supplementary Figure 8.** The effects of education on WMHicv. The error bars indicate ± standard errors around the estimated phenotype-SNP associations.


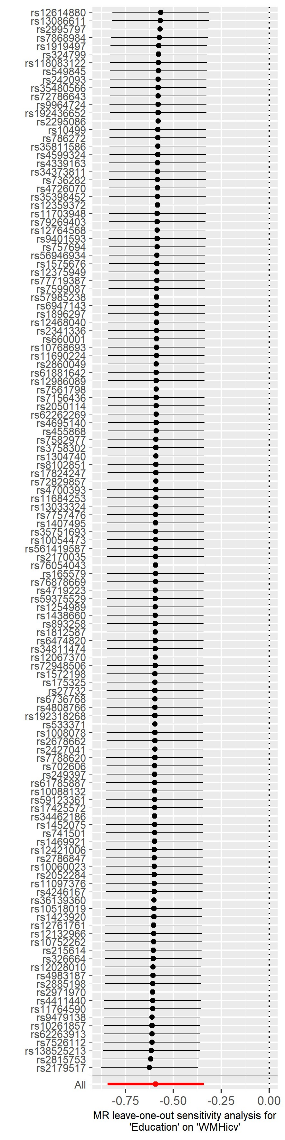


**Leave-one-out sensitivity analysis for Education on WMHicv**

**Supplementary Figure 9.** Leave-one-out plot of the univariate Mendelian randomisation of educational attainment on white matter hyperintensities as a proportion of intracranial volume.

## Occupational prestige on brain structure


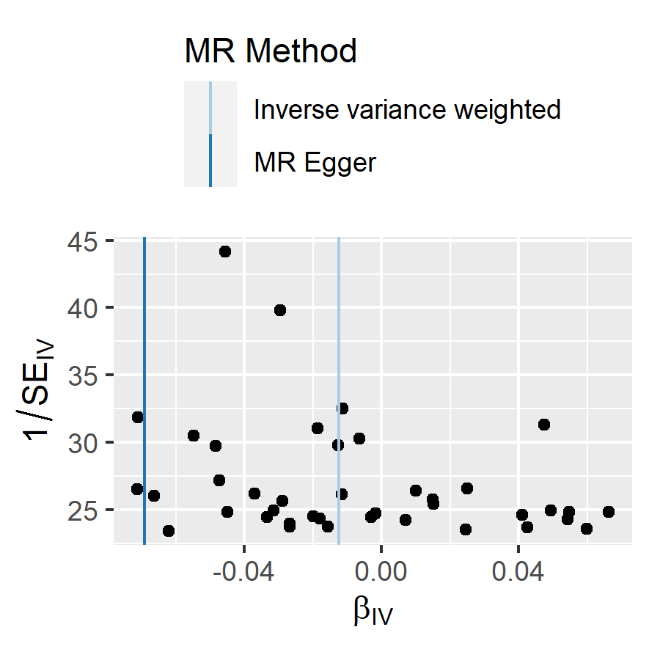


WMHicv

**Supplementary Figure 10.** Funnel plots used to examine the extent to which pleiotropy is balanced across the instruments used in the univariate Mendelian randomisation of Occupational prestige on brain structure.


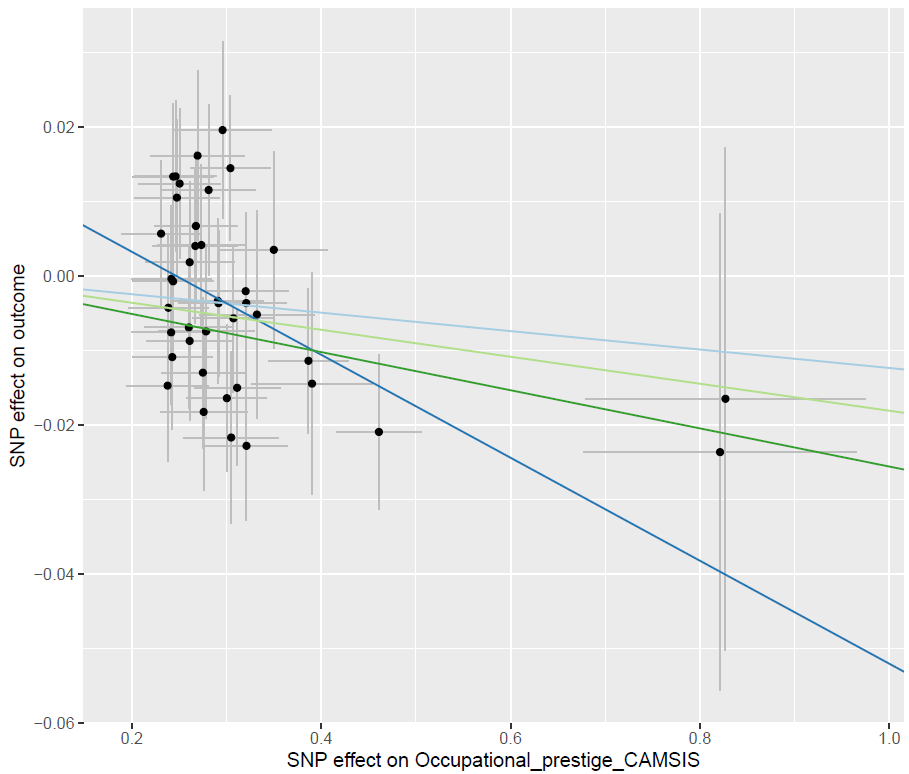

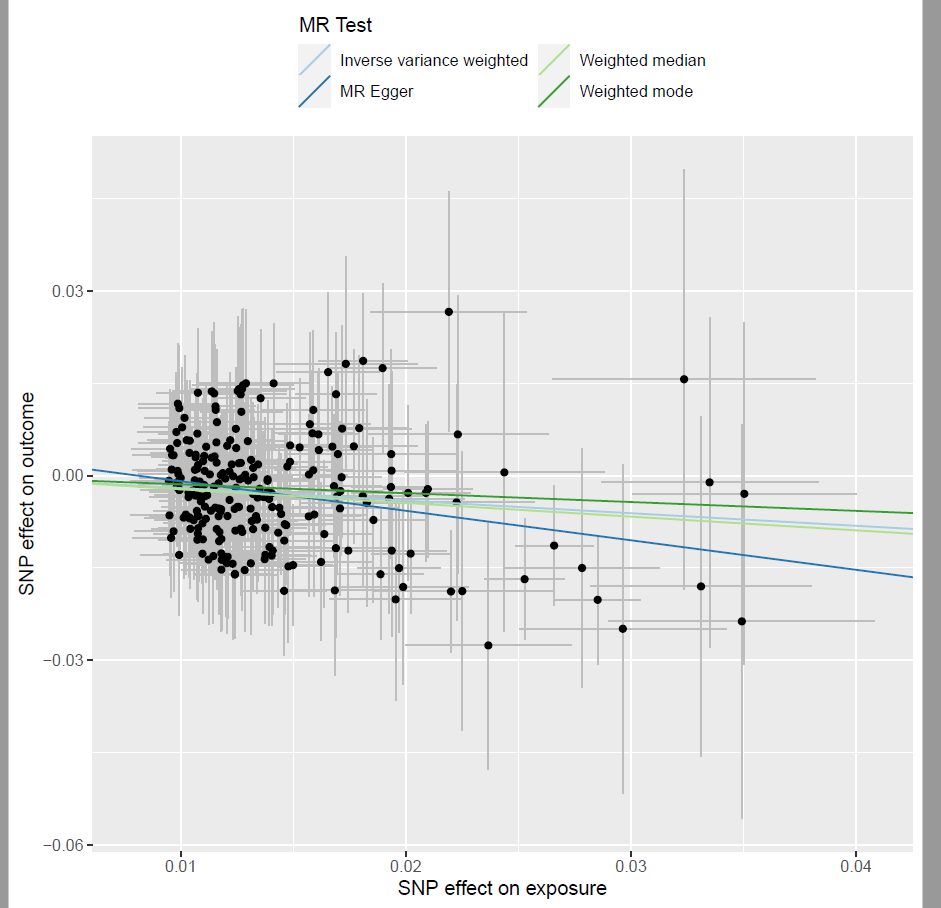


**SNP effect on exposure (Occupation)**

**SNP effect on outcome (WMHicv)**

**Supplementary Figure 2.** The effects of EXPOSURE on OUTCOME. The error bars indicate ± standard errors around the estimated phenotype-SNP associations.

**Supplementary Figure 11.** The effects of occupation on WMHicv. The error bars indicate ± standard errors around the estimated phenotype-SNP associations.


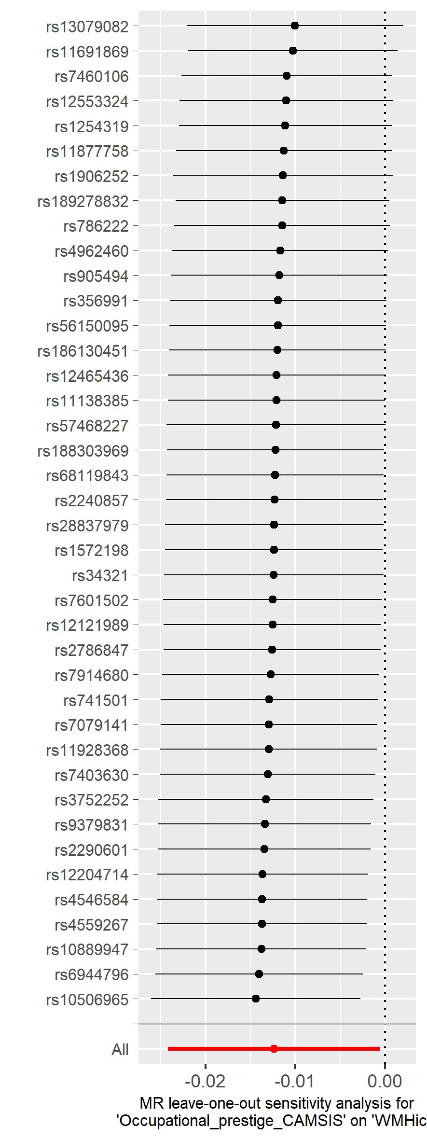


**Leave-one-out sensitivity analysis for Occupation on WMHicv**

**Supplementary Figure 12.** Leave-one-out plot of the univariate Mendelian randomisation of Occupation on white matter hyperintensities as a proportion of intracranial volume.

## Brain structure on socioeconomic status


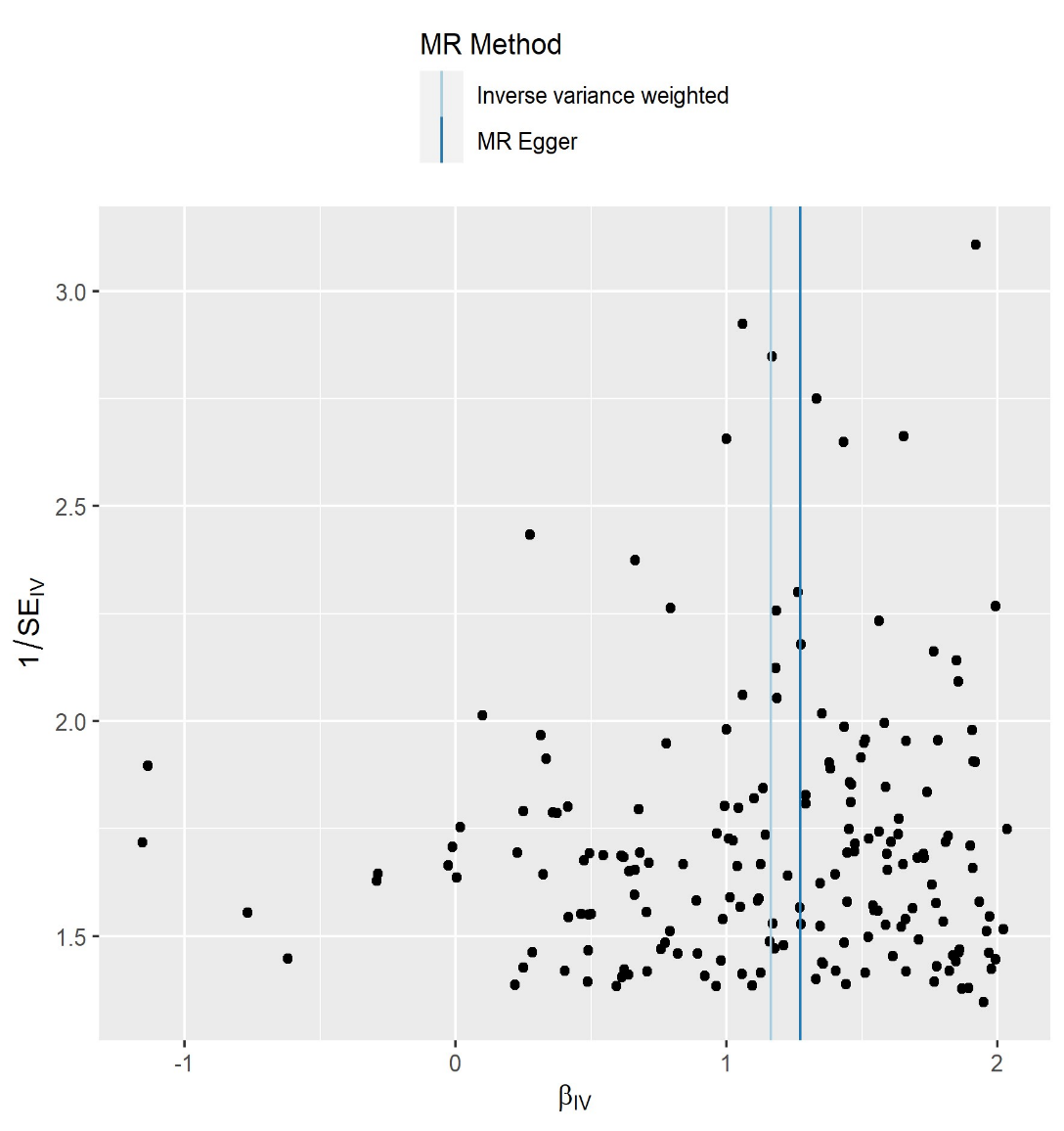

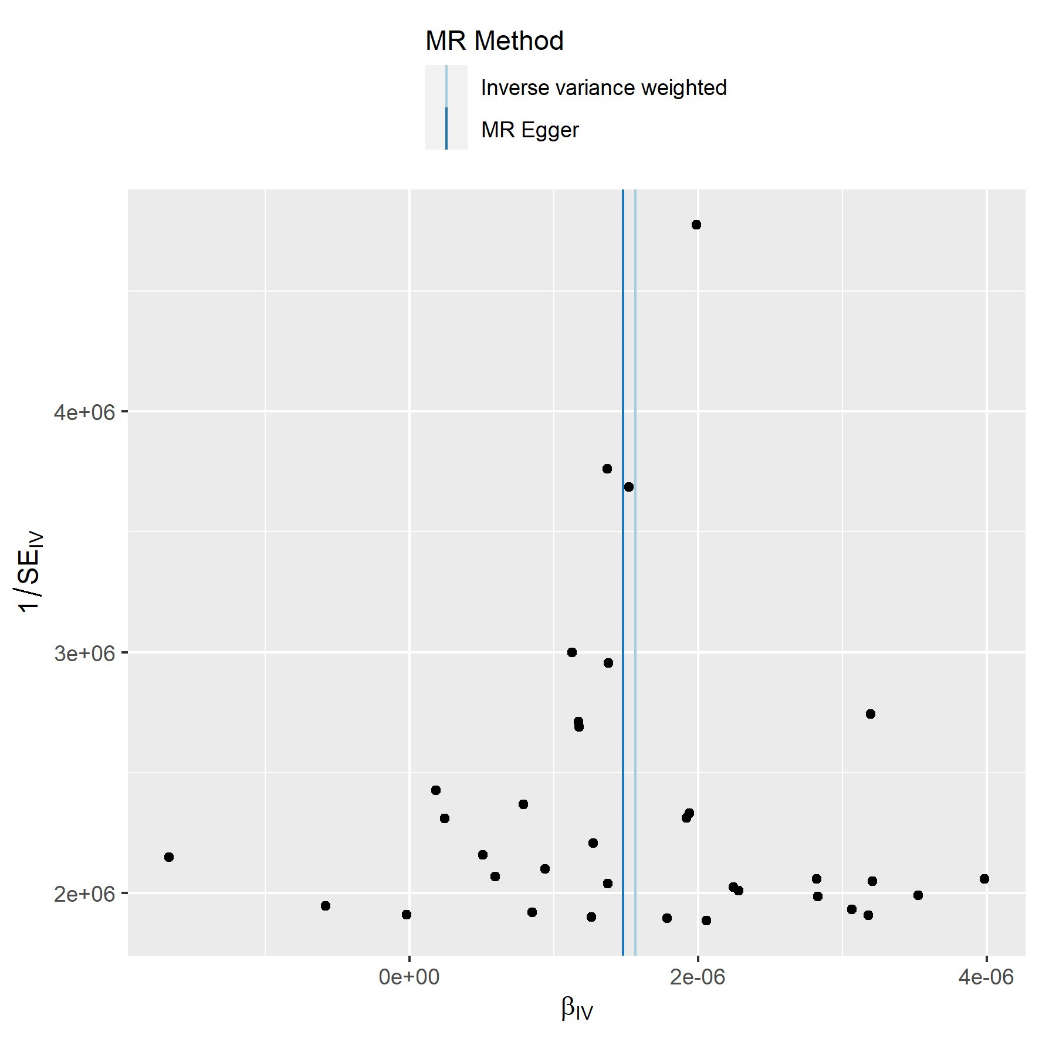


**Supplementary Figure 13.** Funnel plot used to examine the extent to which pleiotropy is balanced across the instruments used in the univariate Mendelian randomisation of total brain volume (TBV) on the general factor of socioeconomic status.


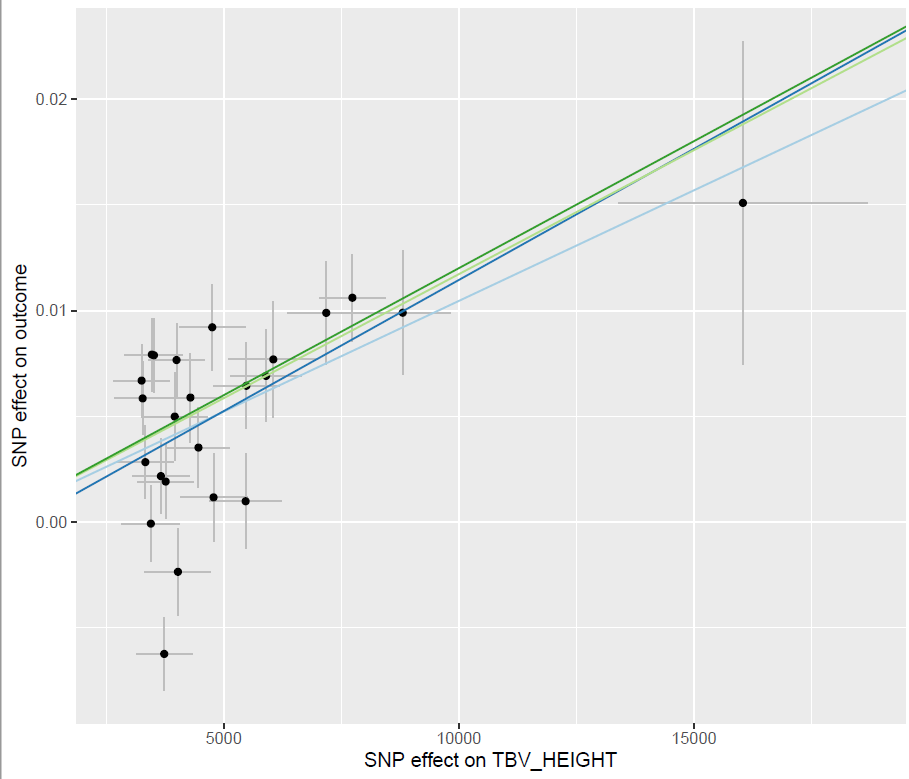

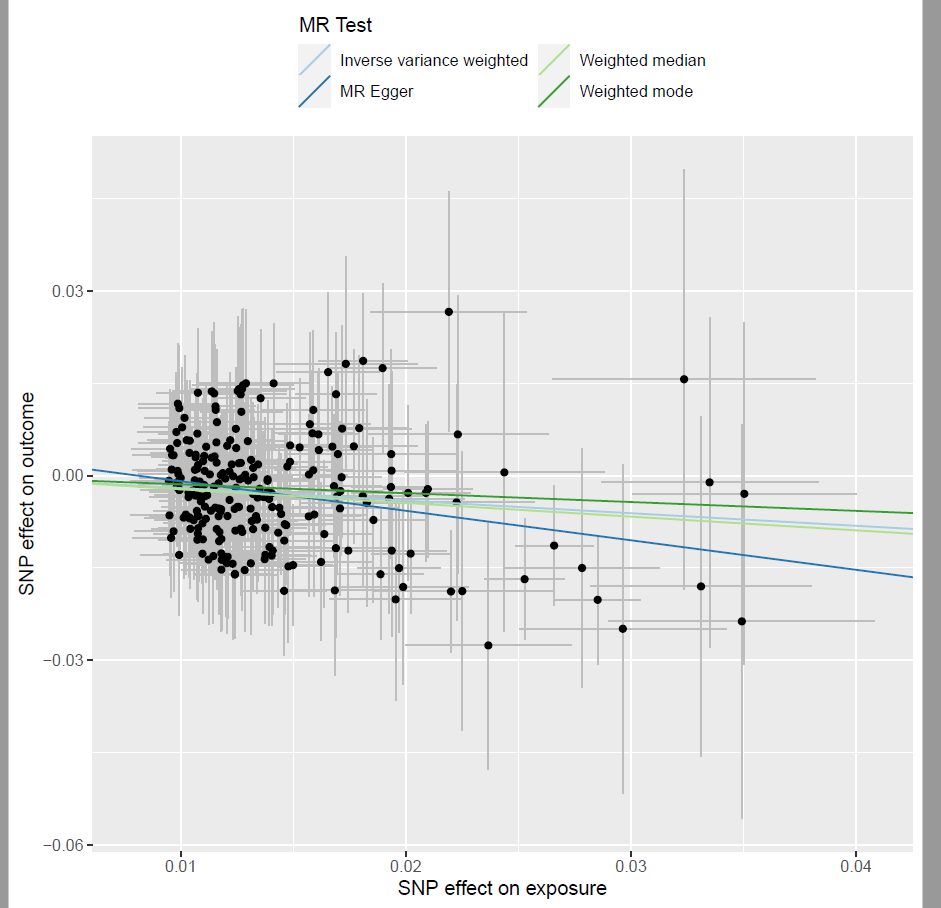


**SNP effect on exposure (TBV)**

**SNP effect on outcome (SES)**

**Supplementary Figure 14.** The effects of TBV on SES. The error bars indicate ± standard errors around the estimated phenotype-SNP associations.


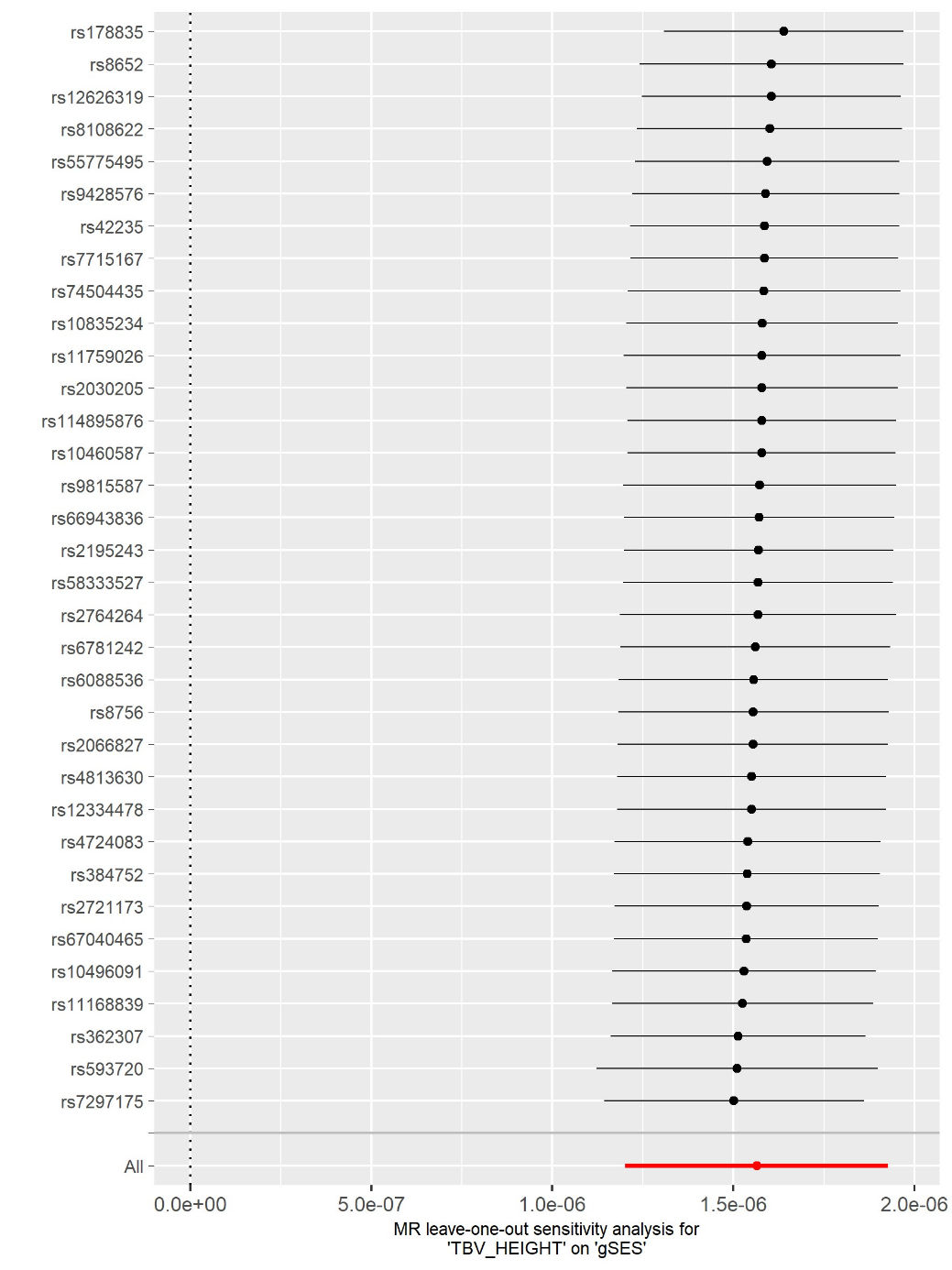


**Leave-one-out sensitivity analysis for TBV on SES**

**Supplementary Figure 15.** Leave-one-out plot of the univariate Mendelian randomisation of total brain volume on general factor of socioeconomic status.

## Total brain volume on Occupation, Income, Education, and Social deprivation


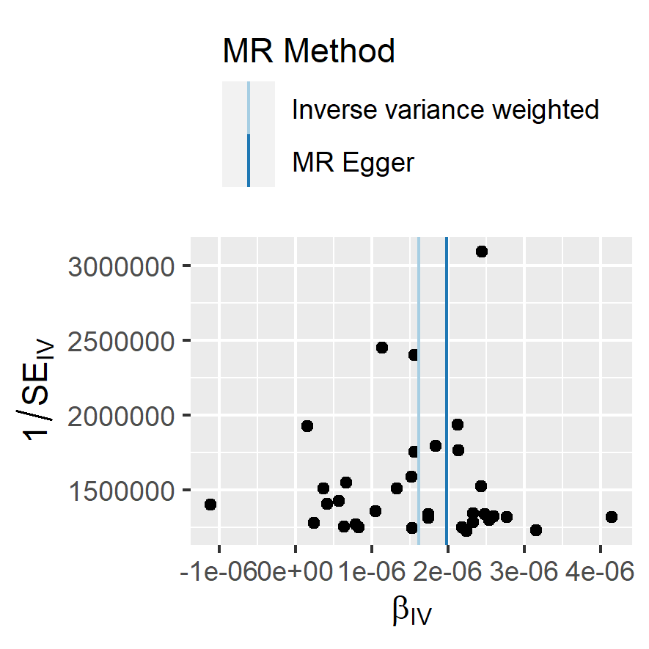


Income


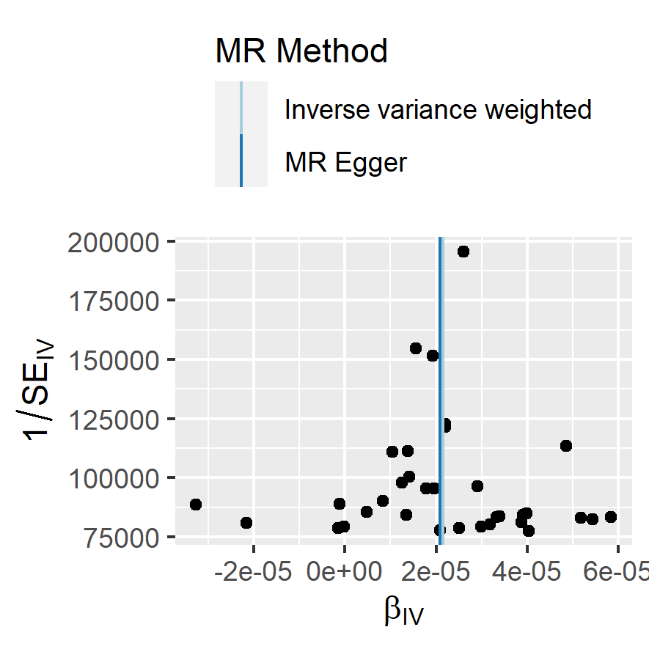


Occupation


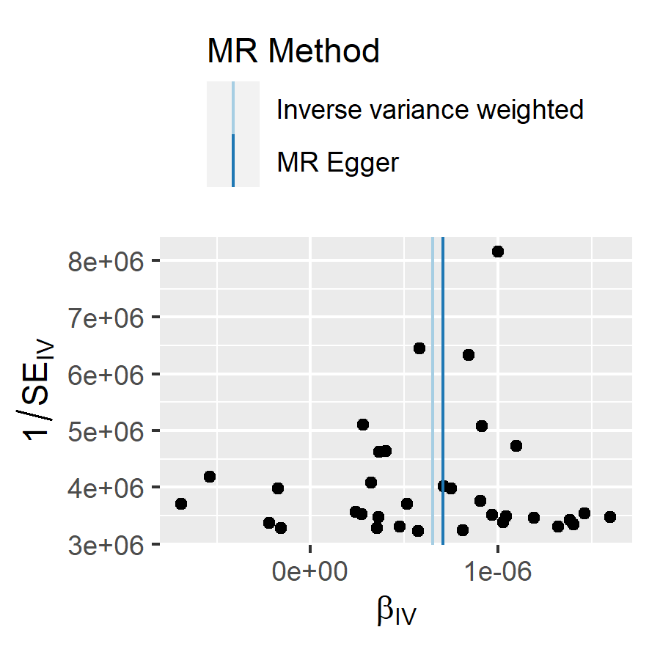


Education


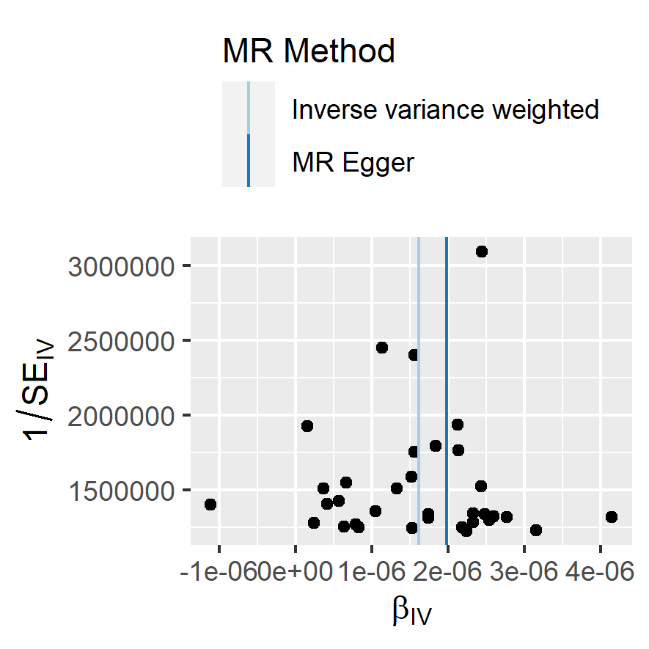


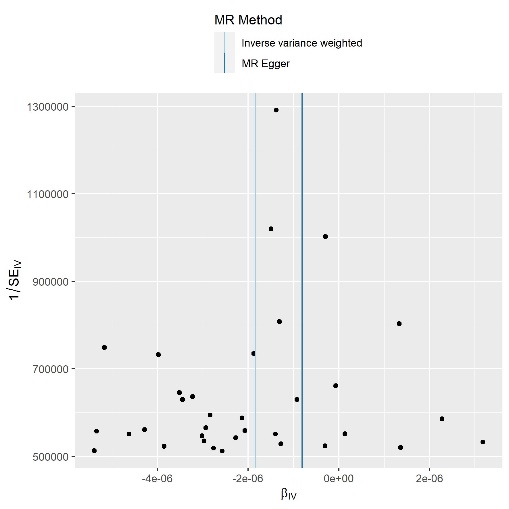


Social deprivation

**Supplementary Figure 16.** Funnel plots used to examine the extent to which pleiotropy is balanced across the instruments used in the univariate Mendelian randomisation of total brain volume on indicators of SES.


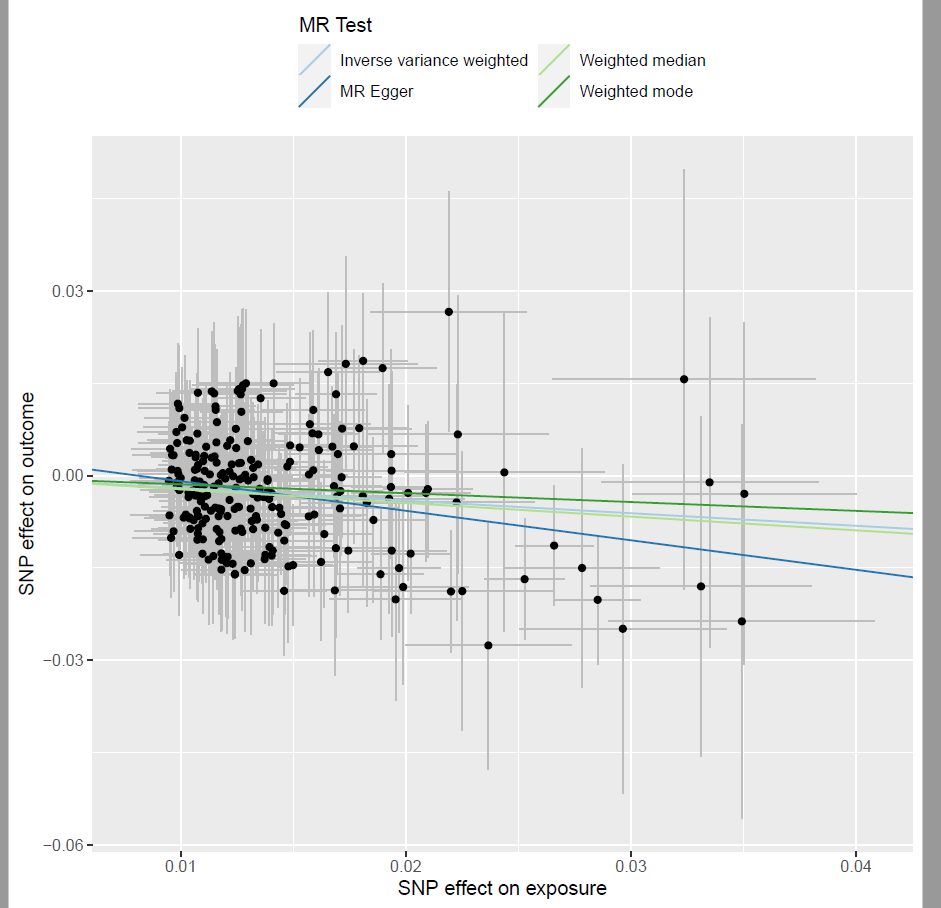

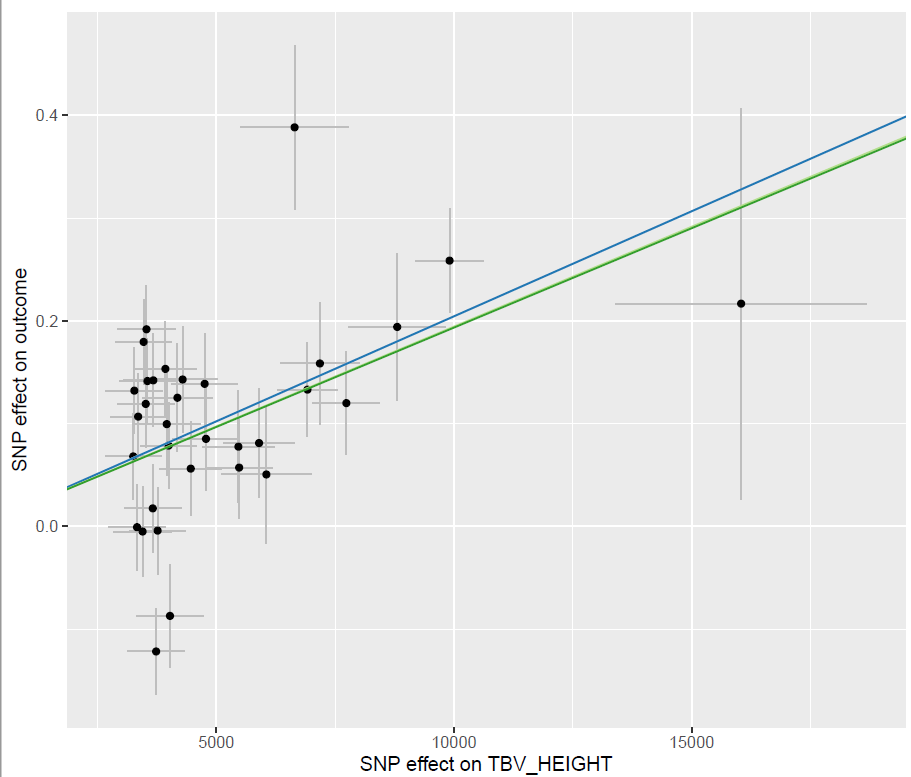


**SNP effect on outcome (Occupation)**

**SNP effect on exposure (TBV)**

**Supplementary Figure 17.** The effects of total brain volume on Occupation. The error bars indicate ± standard errors around the estimated phenotype-SNP associations.


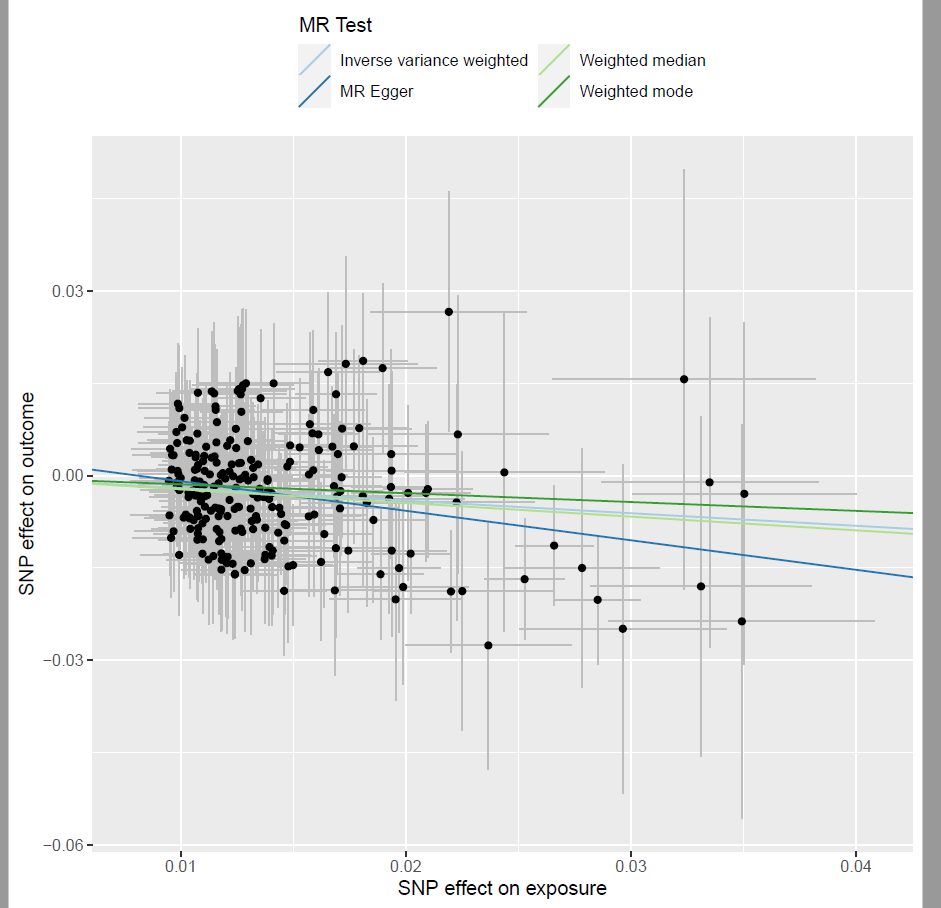

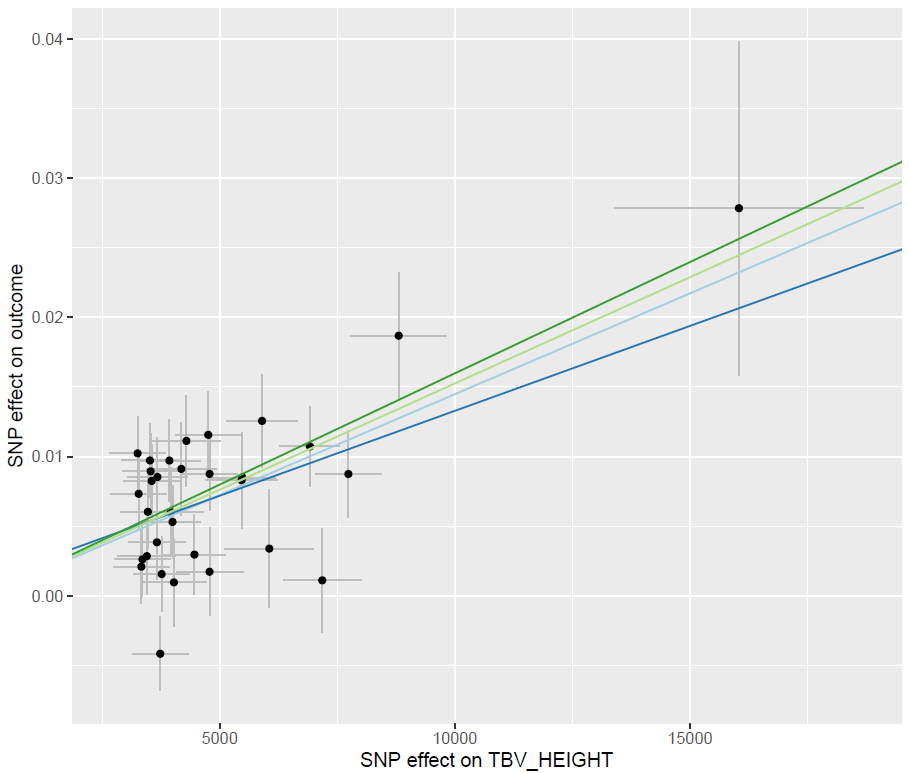


**SNP effect on exposure (TBV)**

**SNP effect on outcome (Income)**

**Supplementary Figure 18.** The effects of total brain volume on Income. The error bars indicate ± standard errors around the estimated phenotype-SNP associations.


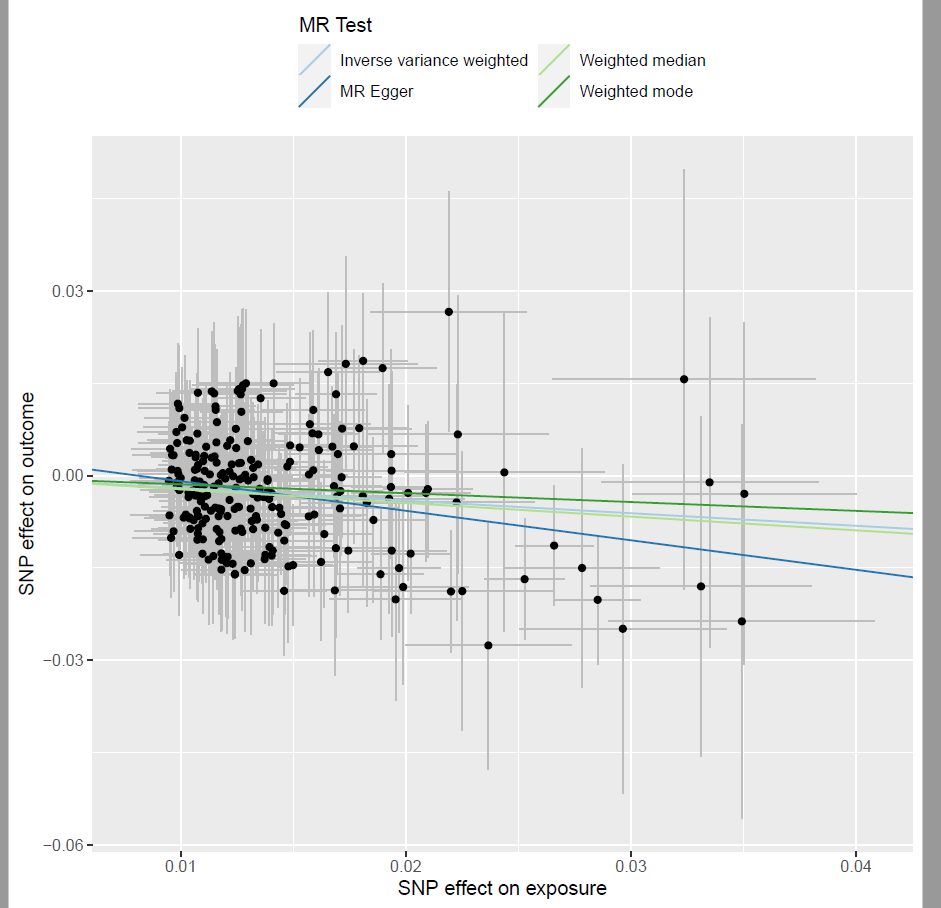

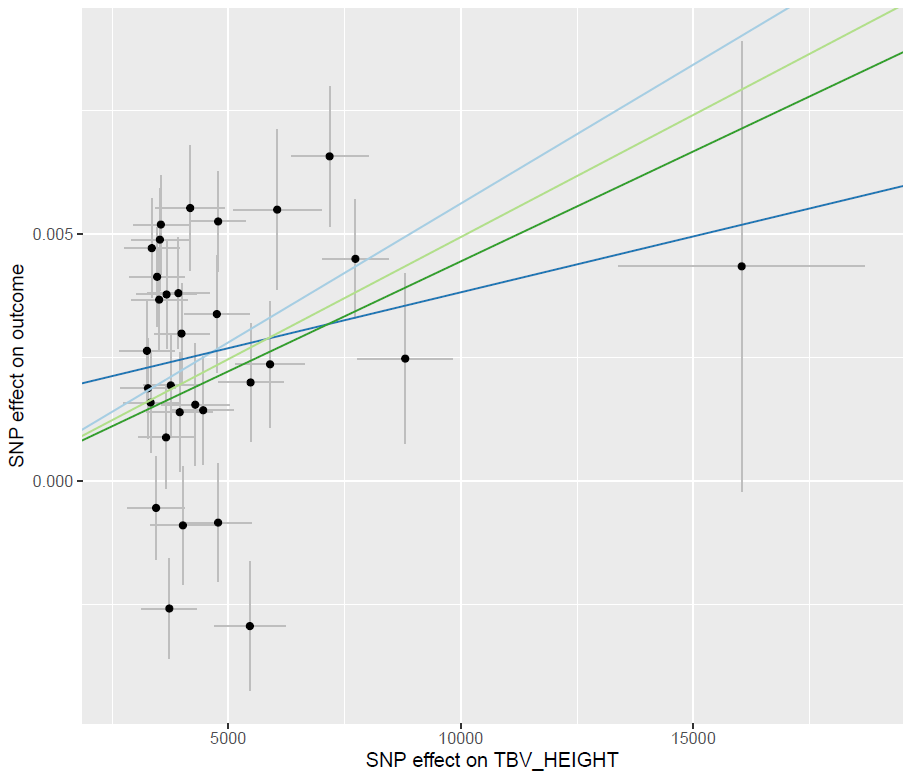


**SNP effect on exposure (TBV)**

**SNP effect on outcome (Education)**

**Supplementary Figure 19.** The effects of total brain volume on Education. The error bars indicate ± standard errors around the estimated phenotype-SNP associations.


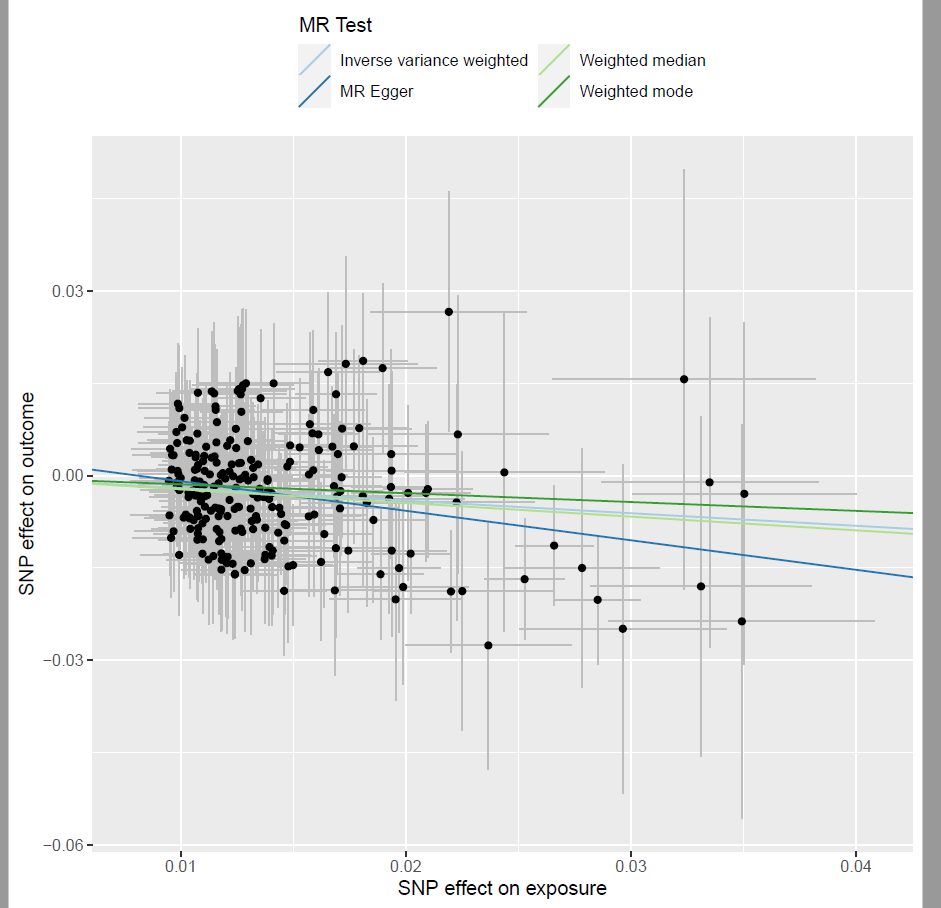

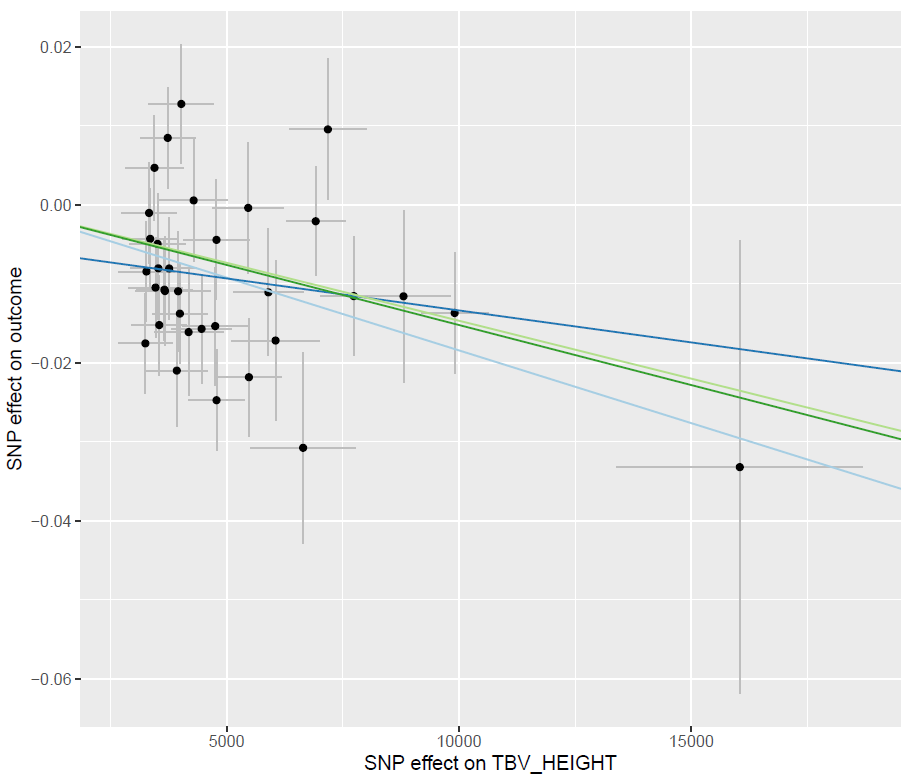


**SNP effect on exposure (TBV)**

**SNP effect on outcome (Social deprivation)**

**Supplementary Figure 20.** The effects of total brain volume on Social deprivation. The error bars indicate ± standard errors around the estimated phenotype-SNP associations.


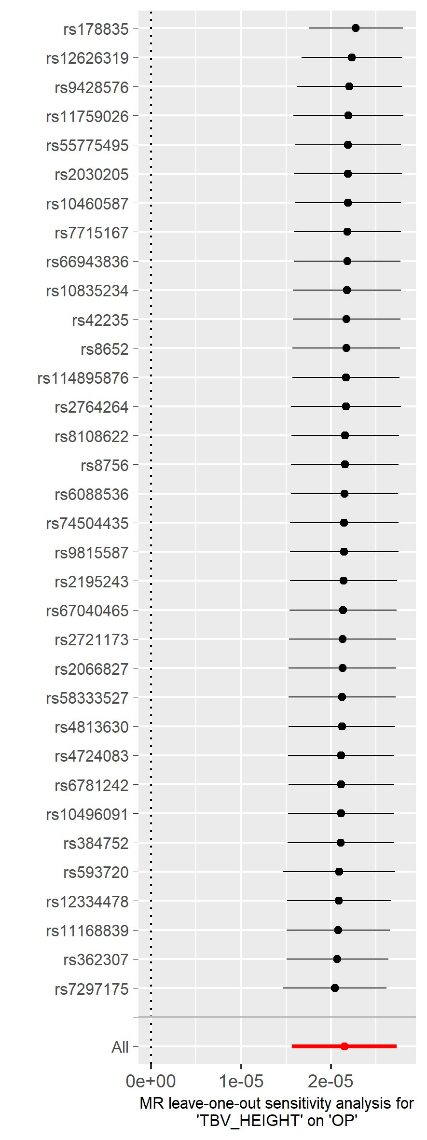

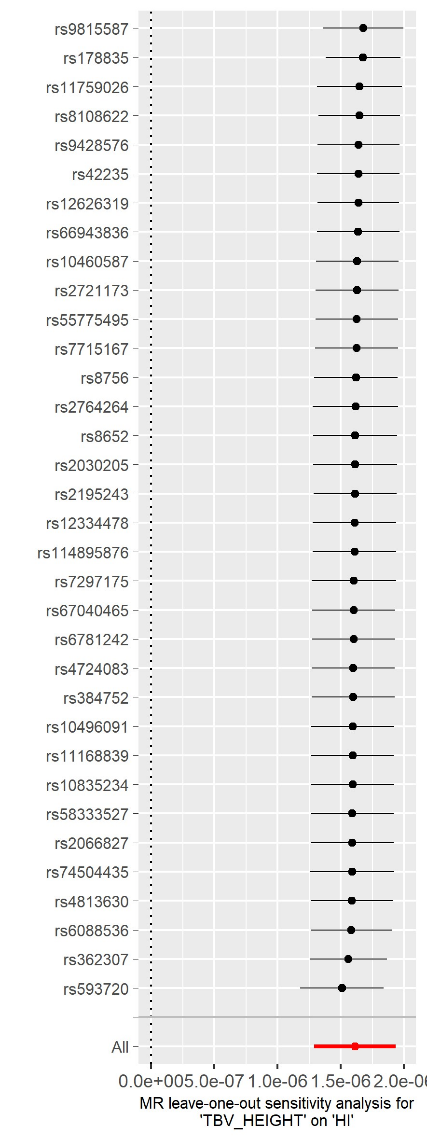

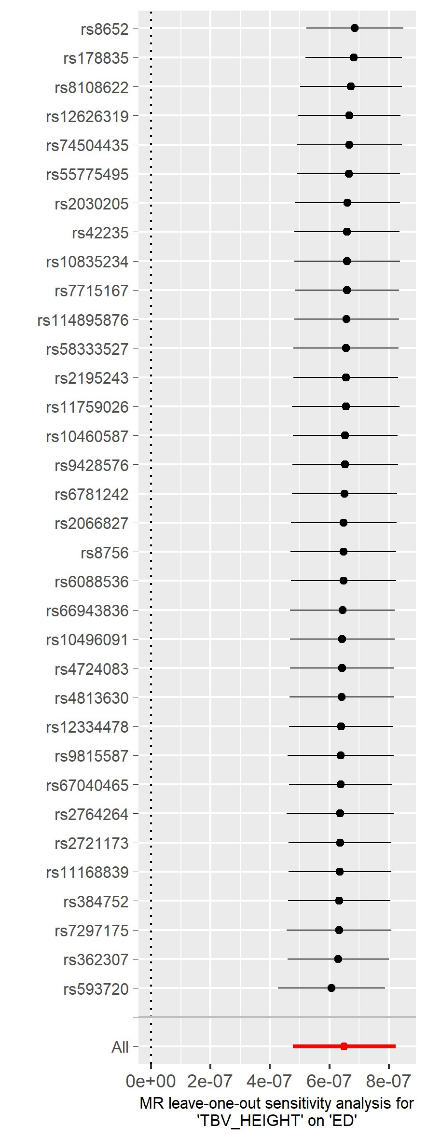


**Leave-one-out sensitivity analysis for TBV on Occupation**

**Leave-one-out sensitivity analysis for TBV on Income**

**Leave-one-out sensitivity analysis for TBV on Education**

**Supplementary Figure 21.** Leave-one-out plot of the univariate Mendelian randomisation of total brain volume on occupational prestige, household income, and educational attainment.


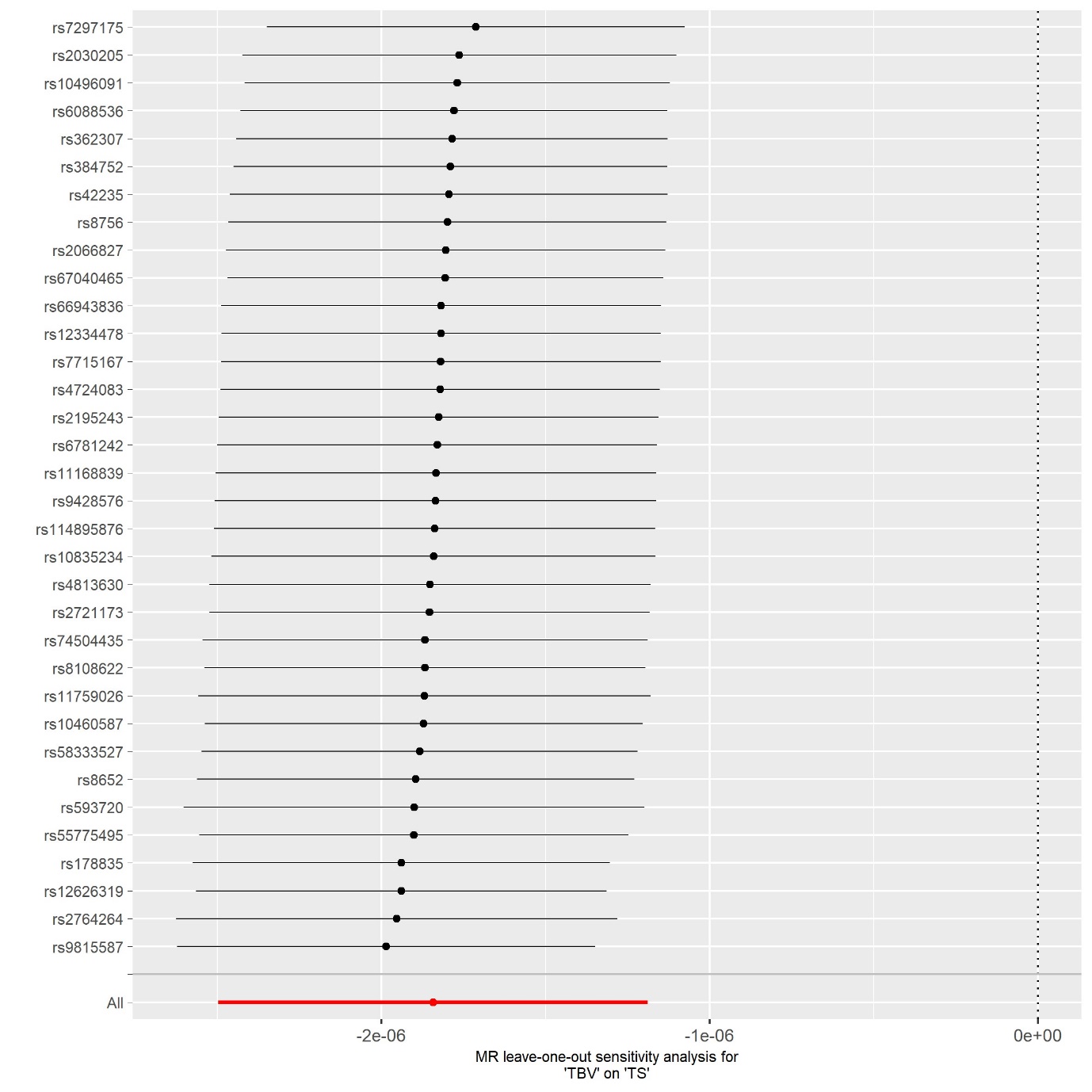


**Leave-one-out sensitivity analysis for TBV on Social deprivation**

**Supplementary Figure 22.** Leave-one-out plot of the univariate Mendelian randomisation of total brain volume on social deprivation.

## Genetic overlap between intelligence and SES traits


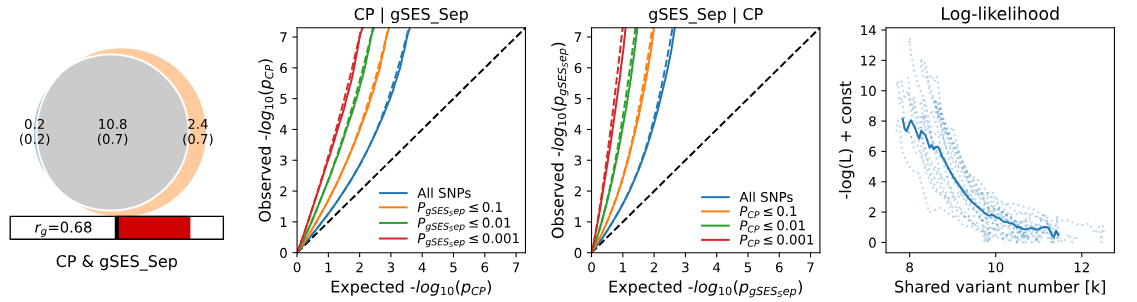

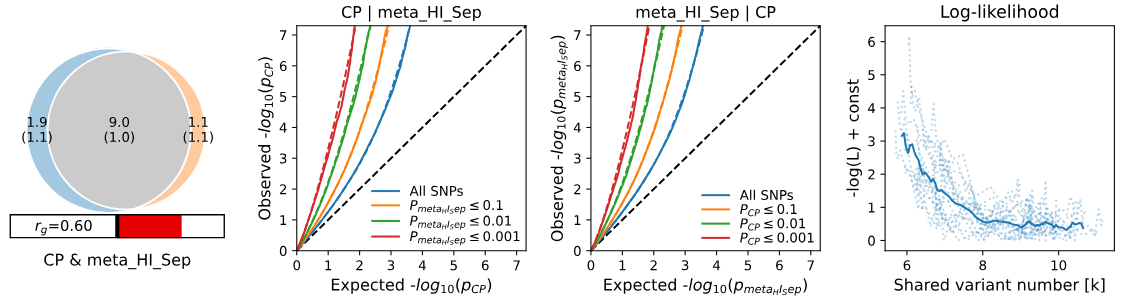


Cognitive ability & Household income


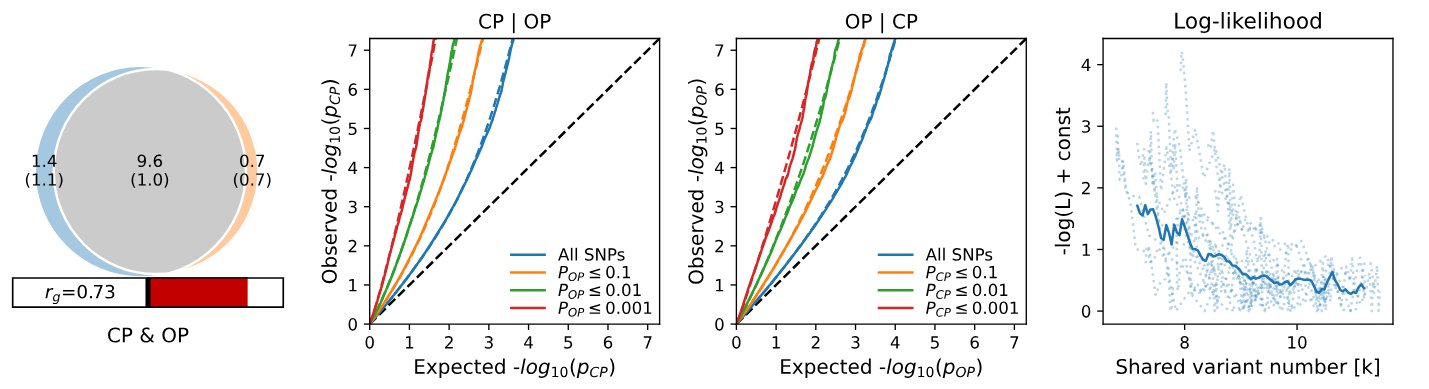

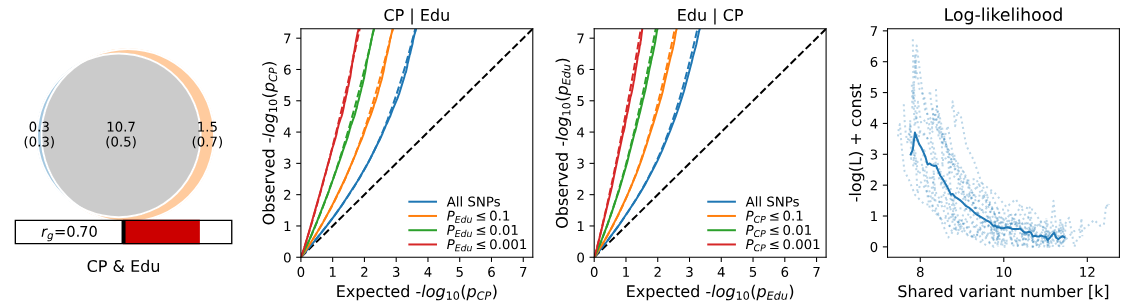

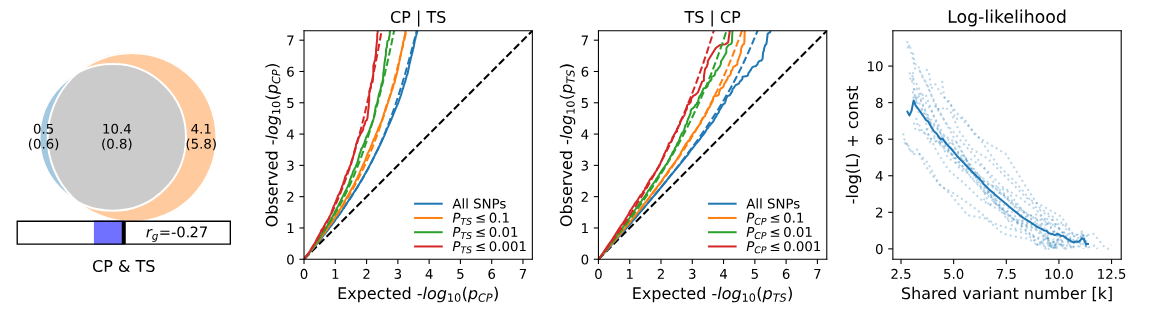


Cognitive ability & Occupational prestige

Cognitive ability & Educational attainment

Cognitive ability & Socioeconomic status

Cognitive ability & Social deprivation

A.

B.

C.

D.

E.

**Supplementary Figure 23.** Mixer results show the genetic overlap of cognitive ability (CP) with Socioeconomic status (gSES) in panel a, occupational prestige (OP) in panel b, household income (HI) in panel c, educational attainment (Edu) in panel d, and social deprivation (TS) in panel e. In each panel, the first plot shows the genetic overlap. Number of unique CP variants is shown in blue, number of unique SES variants is shown in orange, and number of shared variants is shown in grey. The unit is in thousand and the standard error of the estimate is in brackets. The bar beneath the Venn diagram shows the genetic correlation between CP and SES. The second plot is the Q-Q plot of CP GWAS condition on SES variants. The third plot is the Q-Q plot of SES GWAS condition on CP variants. The fourth plot shows the negative log likelihood ratio of the genetic overlap model. Model with the lowest negative log likelihood ratio is defined as the best model.

## Cognitive ability on measures on gSES, Occupation, Income, Education, and Social deprivation


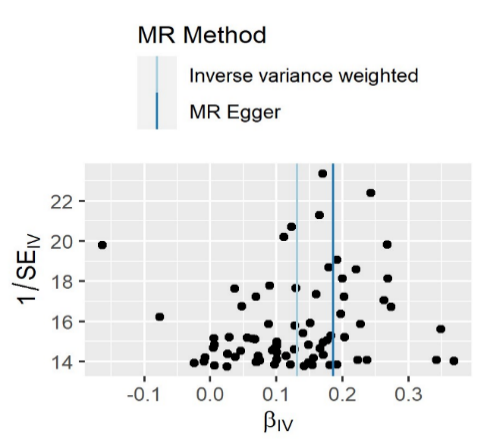


Socioeconomic status


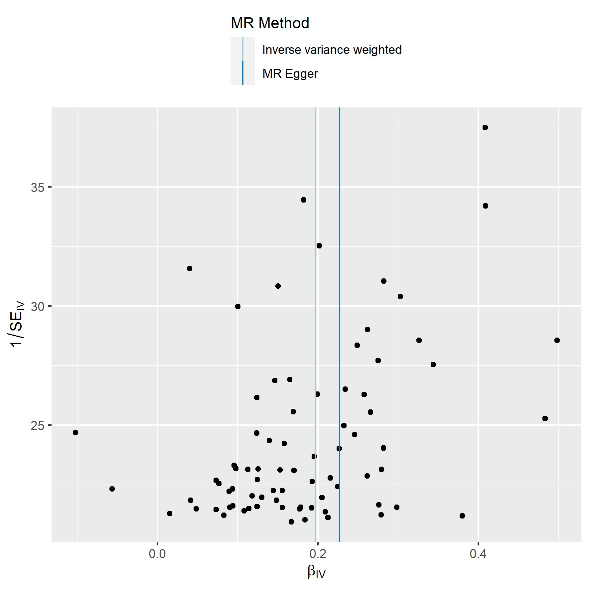

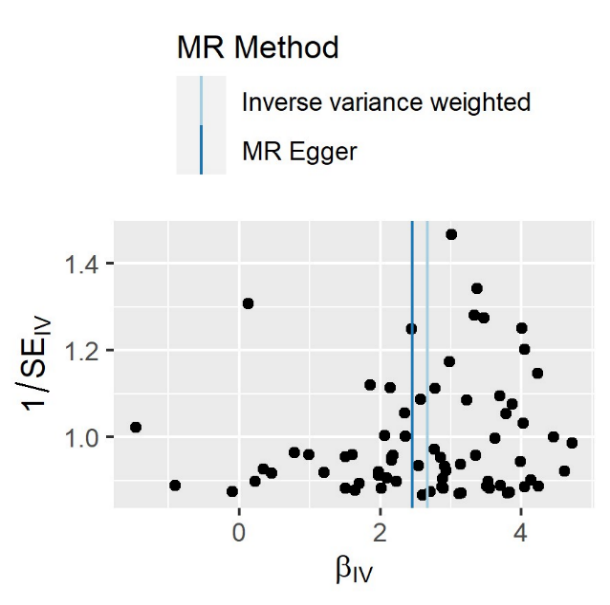


Occupational prestige


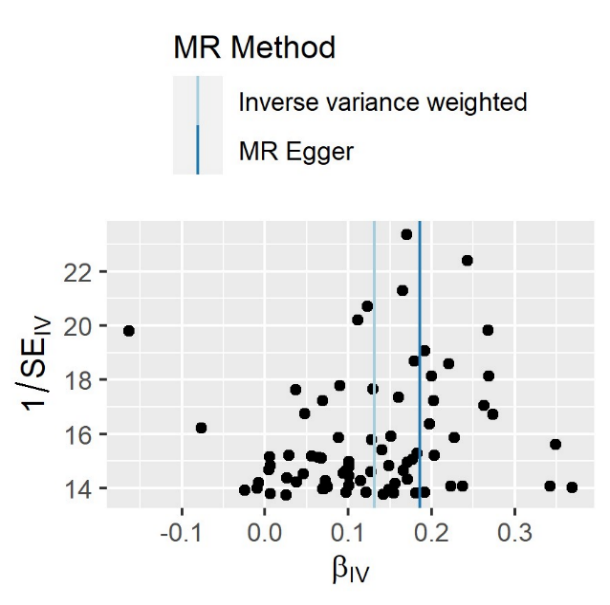


Household income


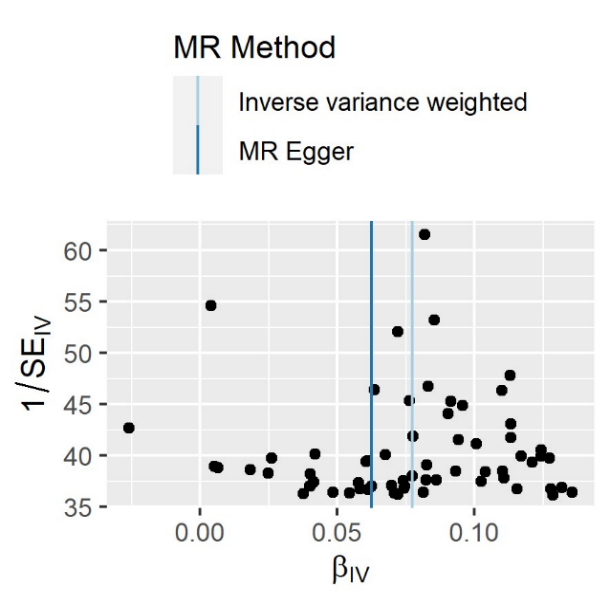


Educational attainment


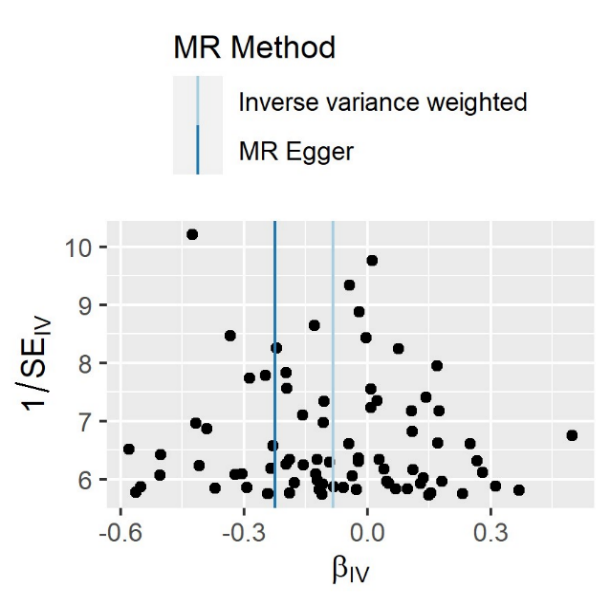


Social deprivation

**Supplementary Figure 24.** Funnel plots used to examine the extent to which pleiotropy is balanced across the instruments used in the univariate Mendelian randomisation of cognitive ability on the general factor of SES and on indicators of SES.


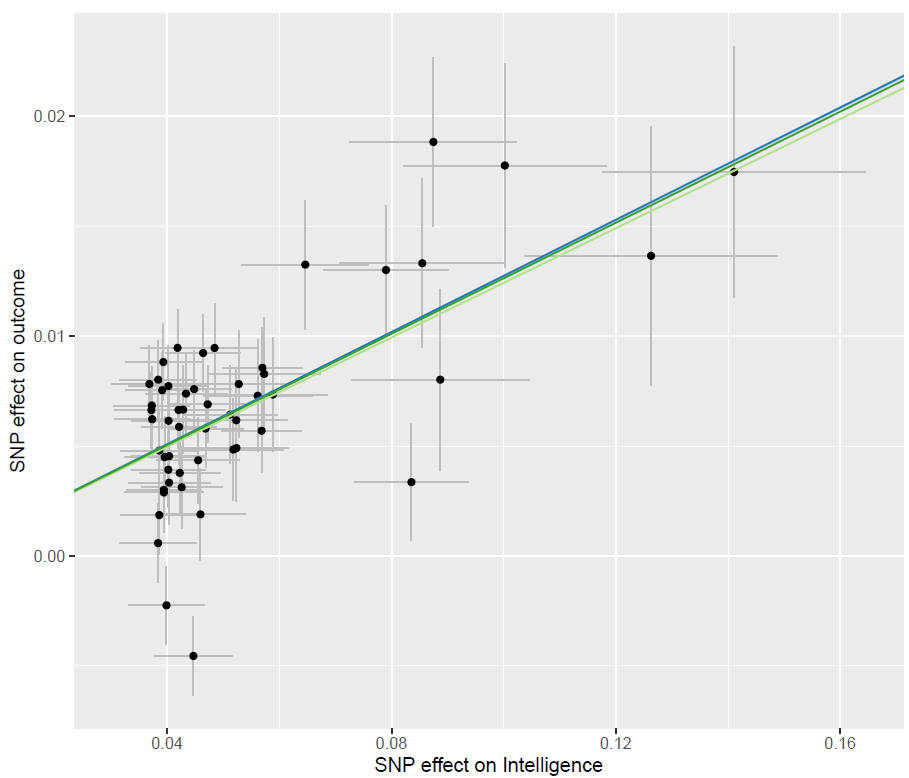

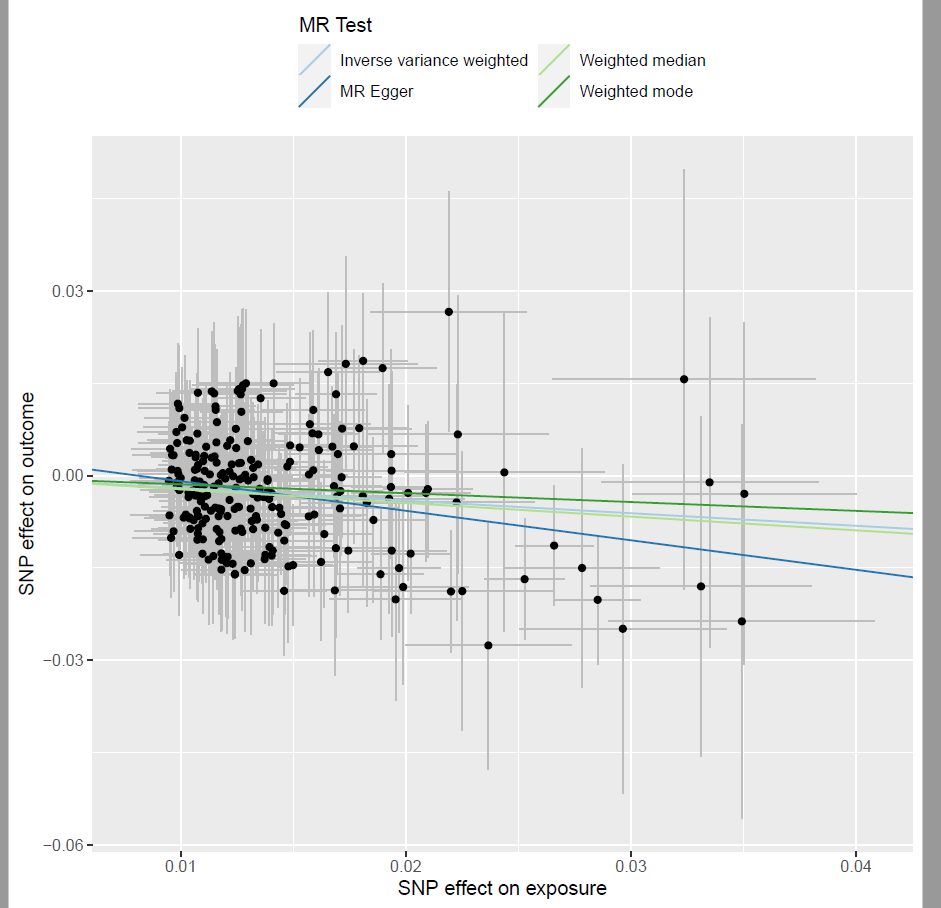


**SNP effect on exposure (Cognitive ability)**

**SNP effect on outcome (SES)**

**Supplementary Figure 25.** The effects of Cognitive ability on SES. The error bars indicate ± standard errors around the estimated phenotype-SNP associations.


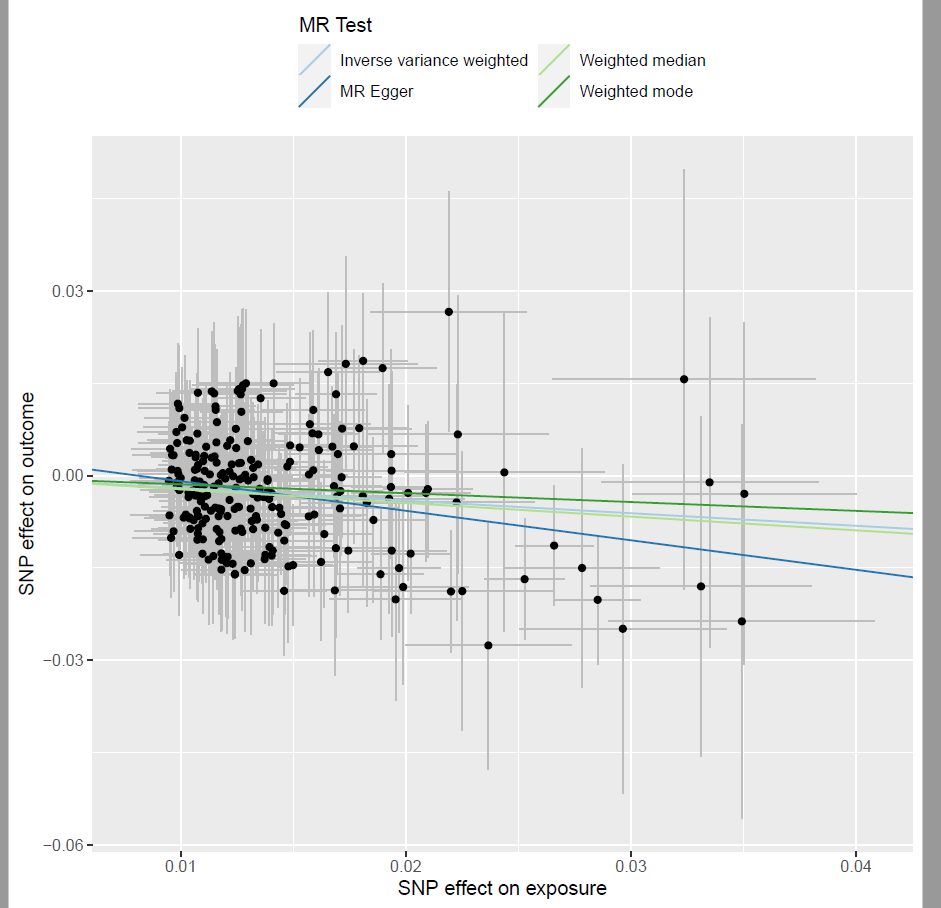

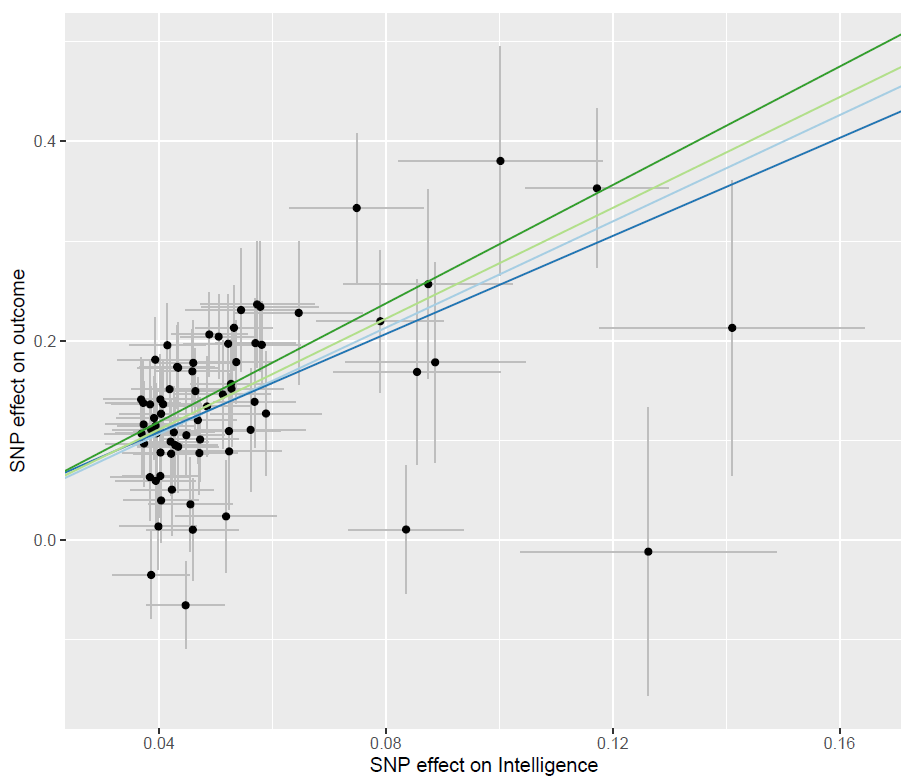


**SNP effect on exposure (Cognitive ability)**

**SNP effect on outcome (Occupation)**

**Supplementary Figure 26.** The effects of Cognitive ability on Occupation. The error bars indicate ± standard errors around the estimated phenotype-SNP associations.


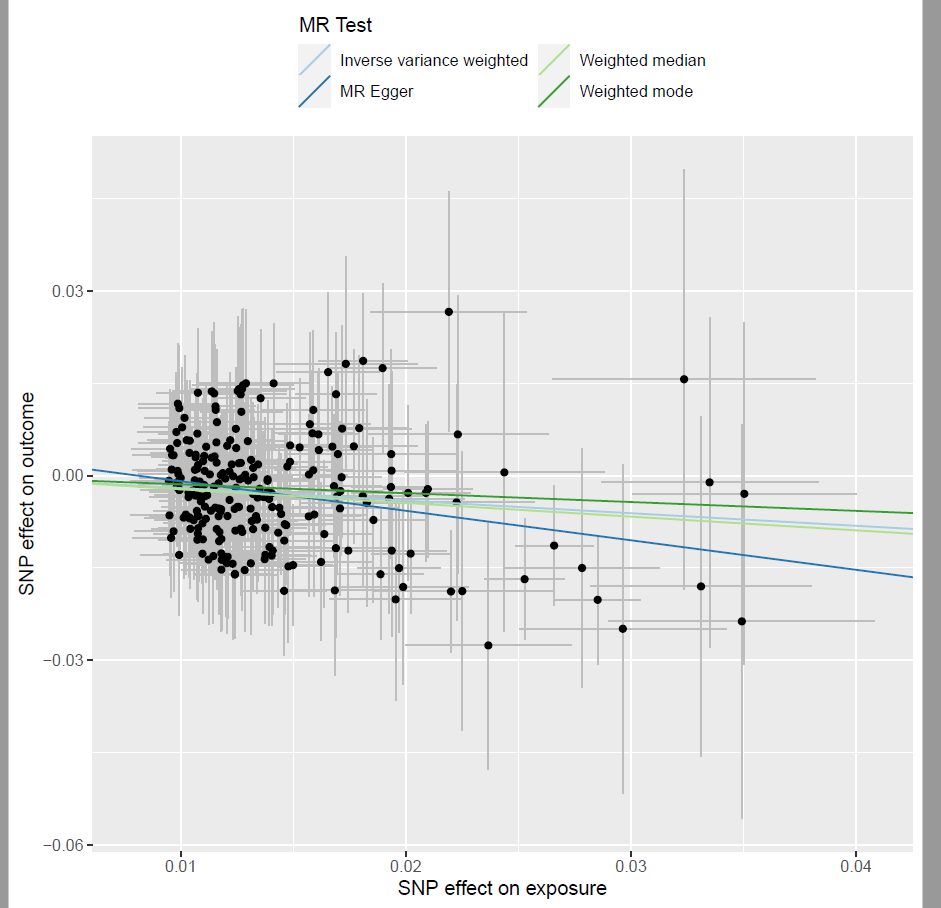

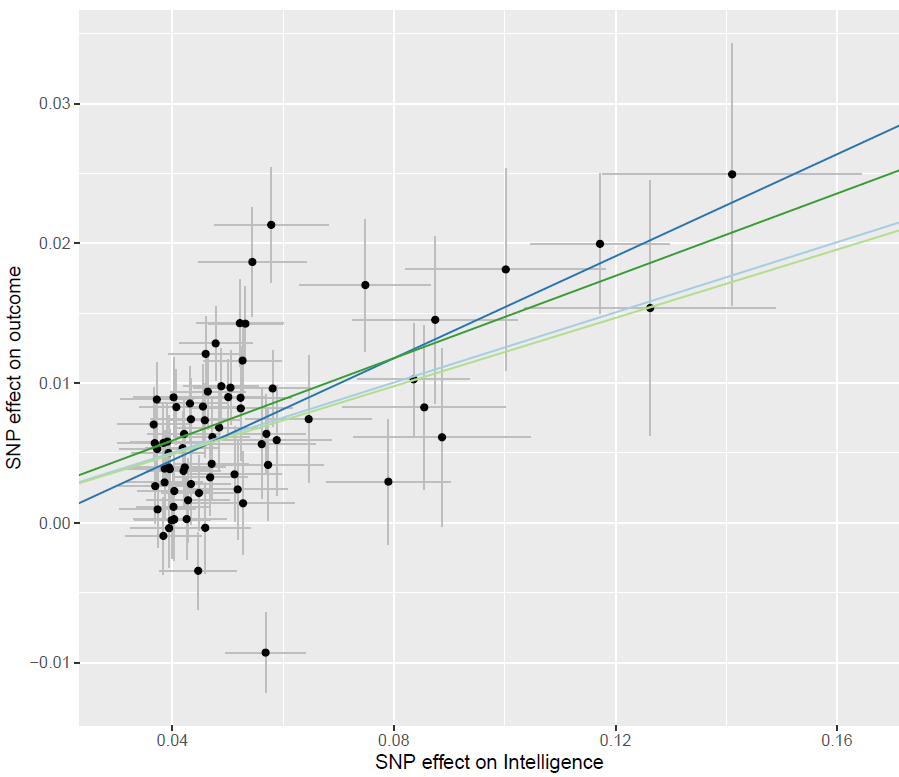


**SNP effect on outcome (Income)**

**SNP effect on exposure (Cognitive ability)**

**Supplementary Figure 27.** The effects of Cognitive ability on Income. The error bars indicate ± standard errors around the estimated phenotype-SNP associations.


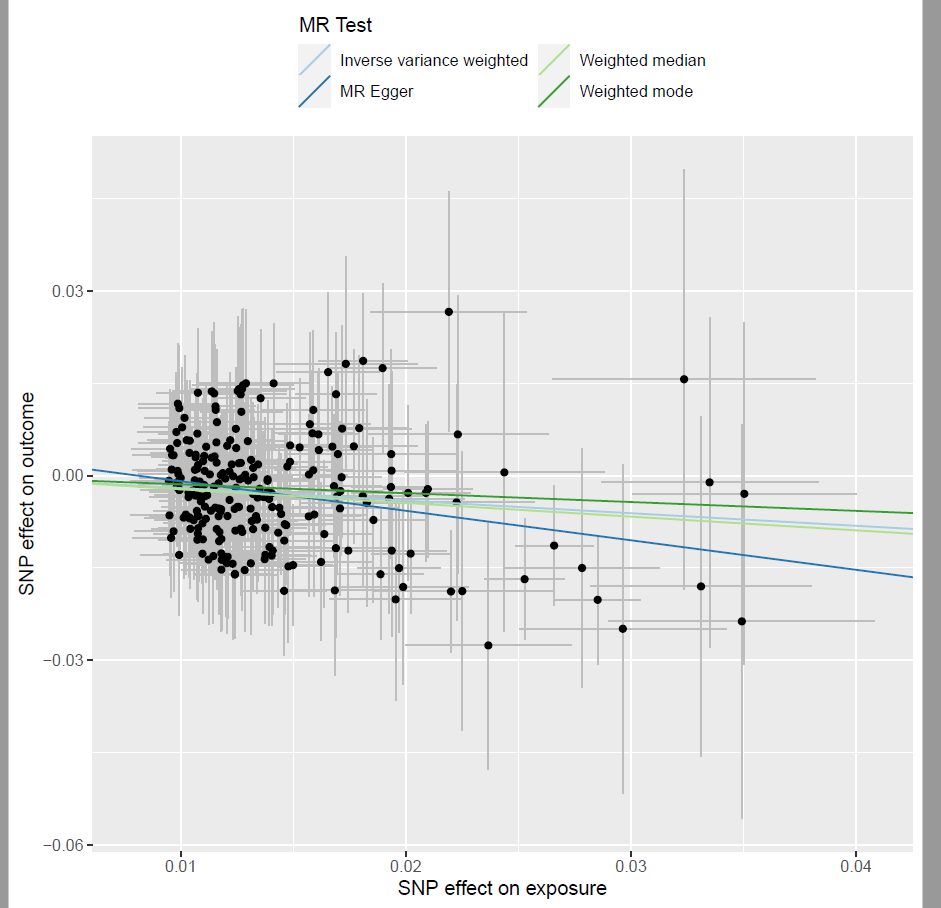

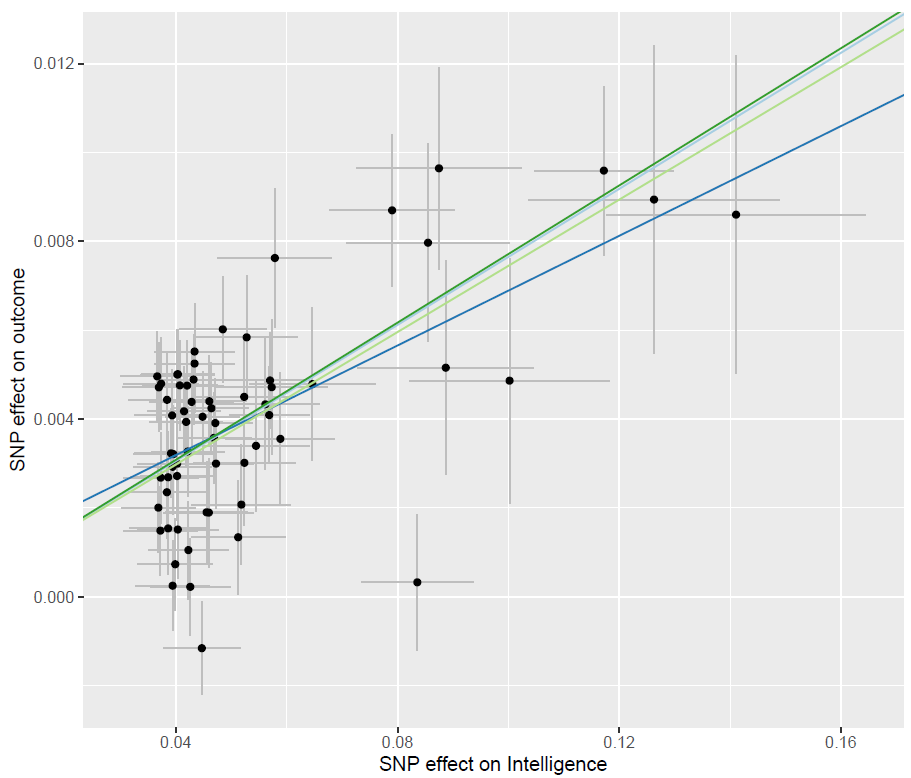


**SNP effect on outcome (Education)**

**SNP effect on exposure (Cognitive ability)**

**Supplementary Figure 28.** The effects of Cognitive ability on Education. The error bars indicate ± standard errors around the estimated phenotype-SNP associations.


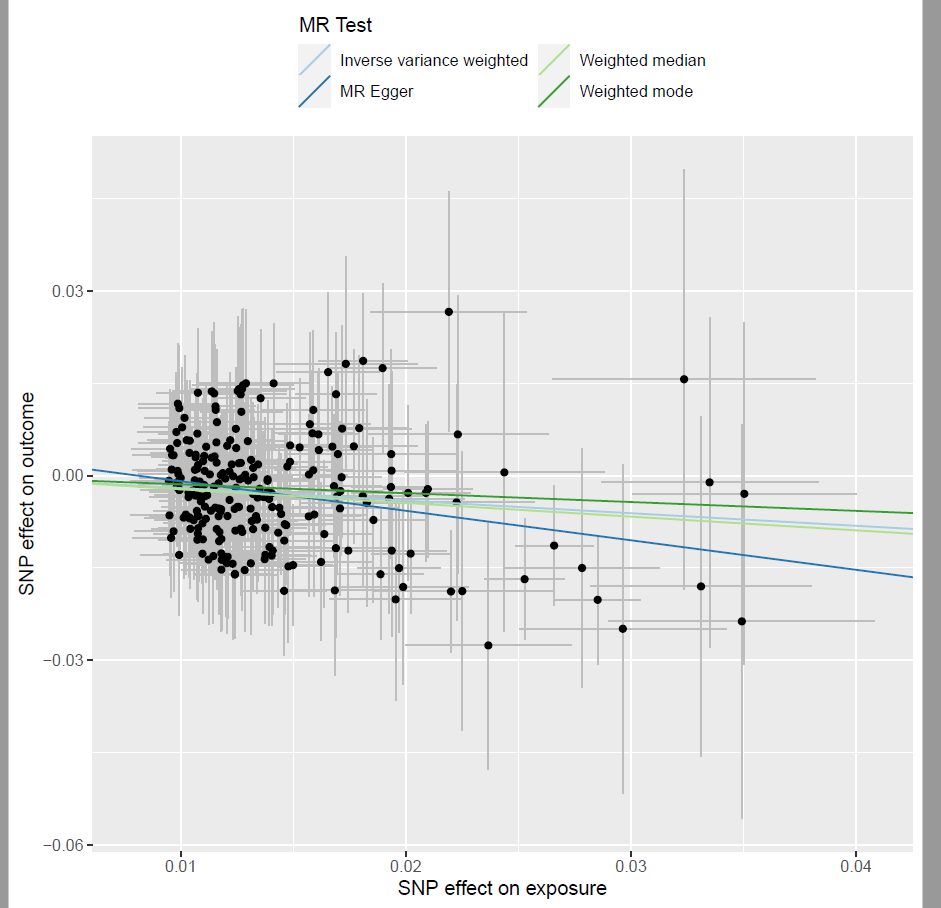

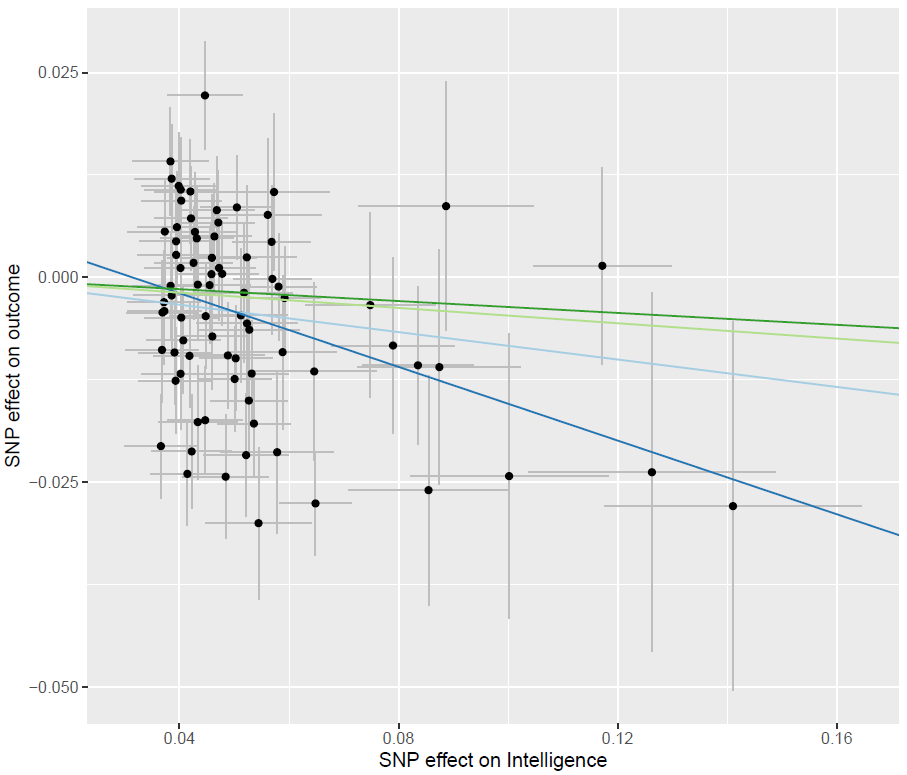


**SNP effect on exposure (Cognitive ability)**

**SNP effect on outcome (Social deprivation)**

**Supplementary Figure 29.** The effects of Cognitive ability on Social deprivation. The error bars indicate ± standard errors around the estimated phenotype-SNP associations.


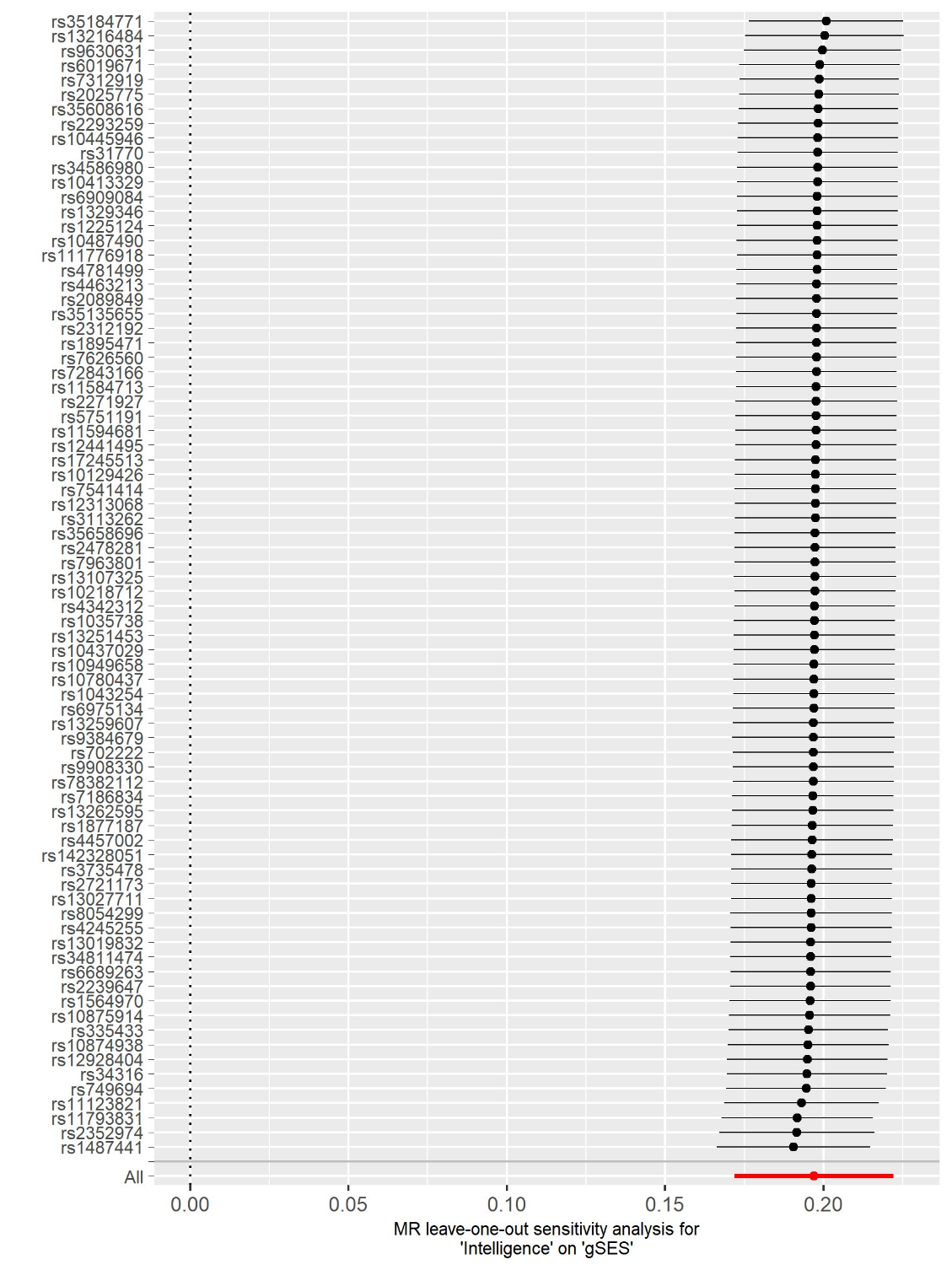


**Leave-one-out sensitivity analysis for Cognitive ability on SES**

**Supplementary Figure 30.** Funnel plots used to examine the extent to which pleiotropy is balanced across the instruments used in the univariate Mendelian randomisation of cognitive ability on the general factor of SES and on indicators of SES.


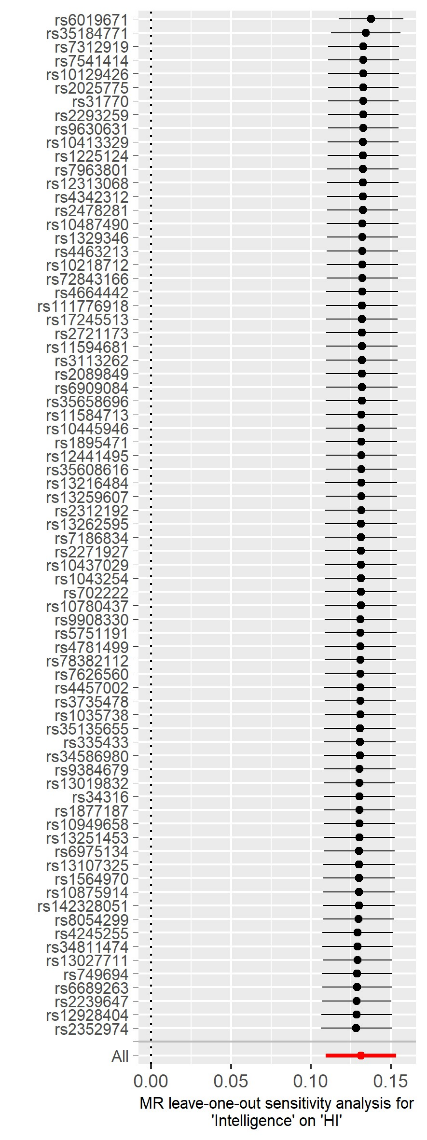

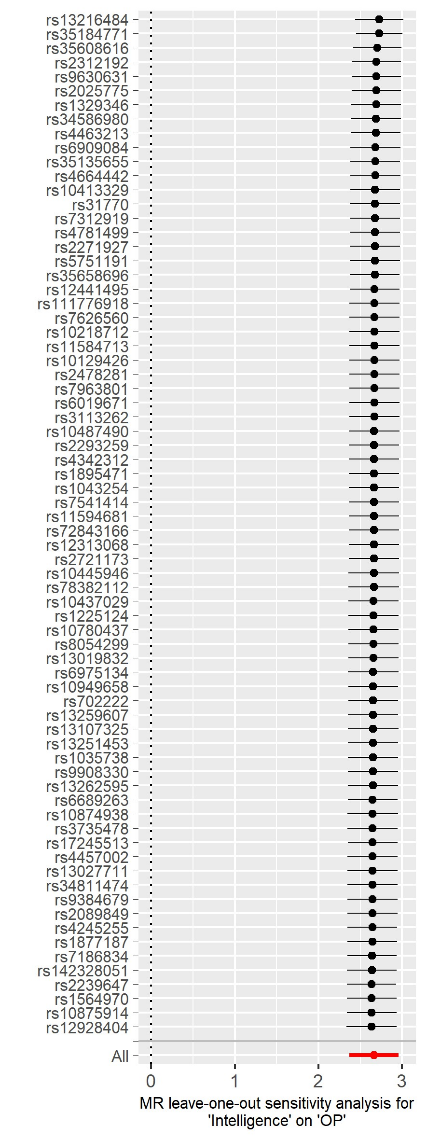

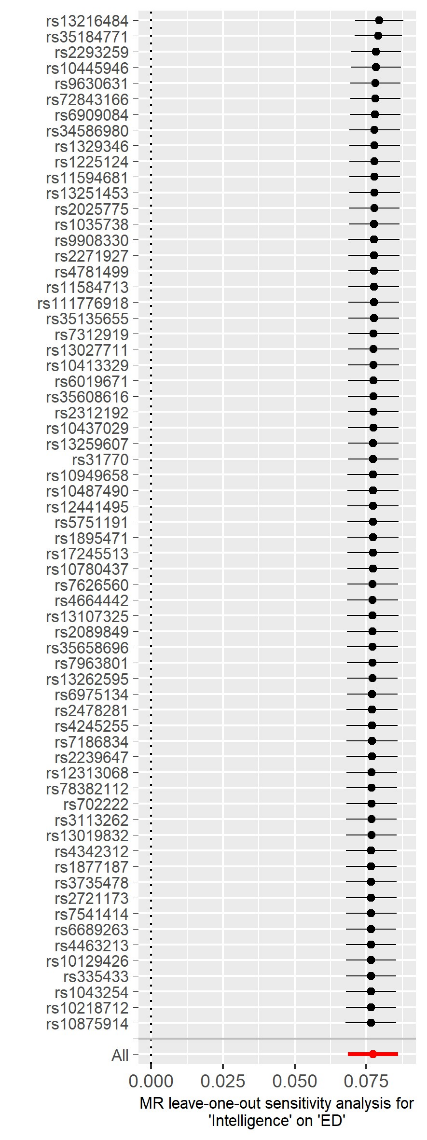

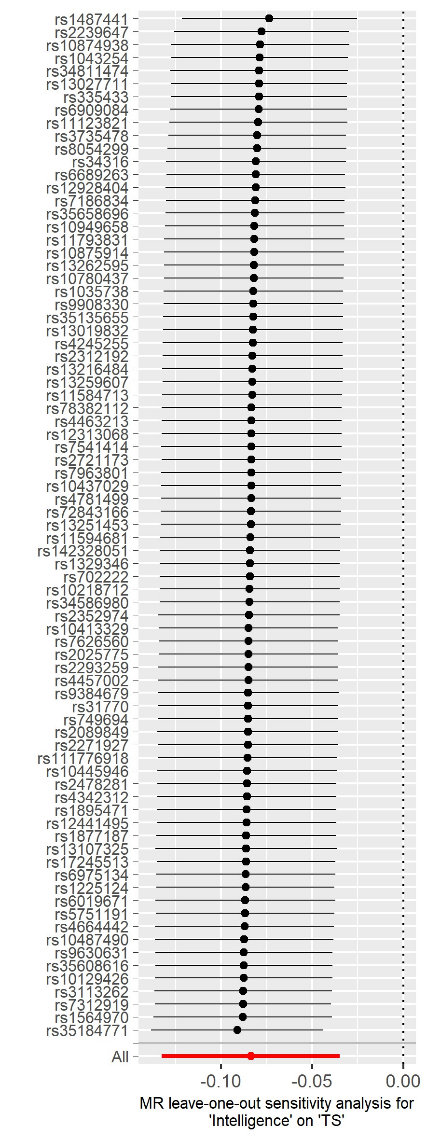


Occupational prestige

Household income

Educational attainment

Social deprivation

**Leave-one-out sensitivity analysis for Cognitive ability on Occupation.**

**Leave-one-out sensitivity analysis for Cognitive ability on Income.**

**Leave-one-out sensitivity analysis for Cognitive ability on Education.**

**Leave-one-out sensitivity analysis for Cognitive ability on Social deprivation.**

**Supplementary Figure 31**. Leave-one-out plot of the univariate Mendelian randomisation of cognitive ability on measures of SES.

## Socioeconomic status on Cognitive ability


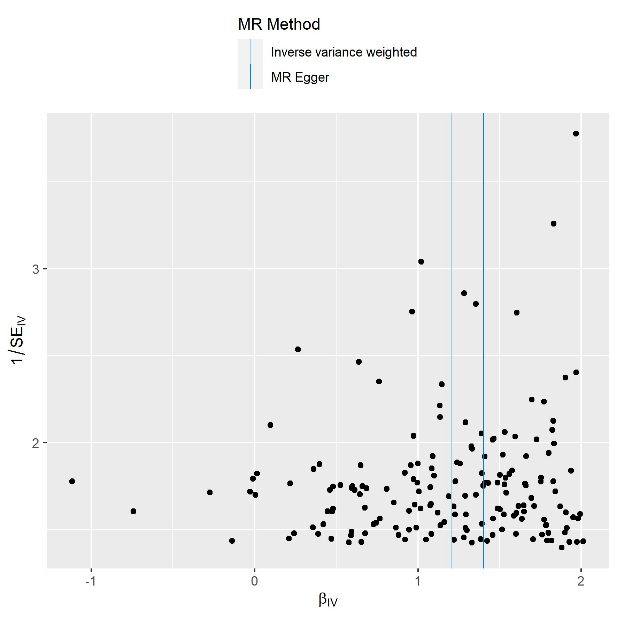


Socioeconomic status


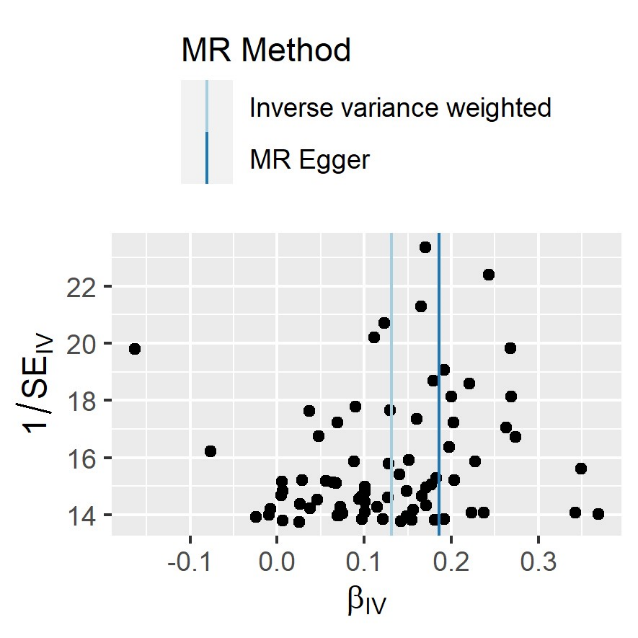

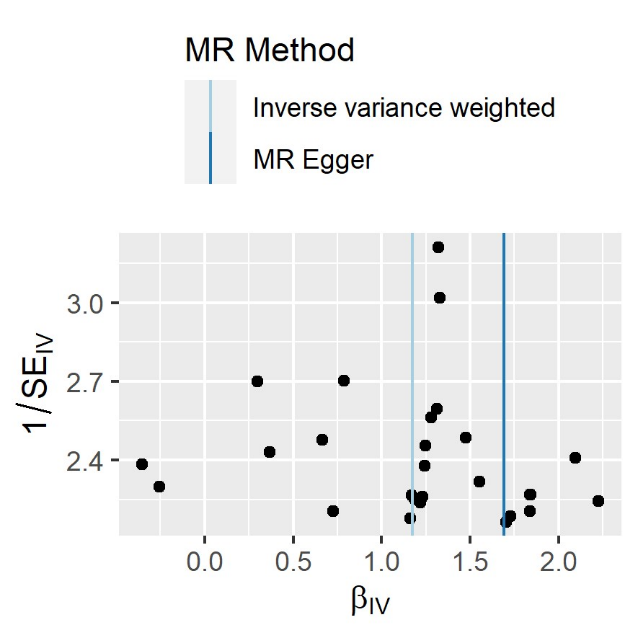


Household income


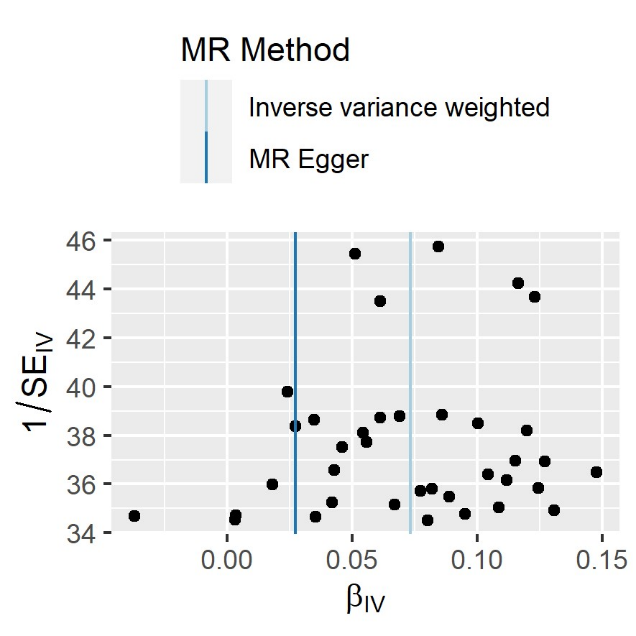


Occupational prestige

Social deprivation


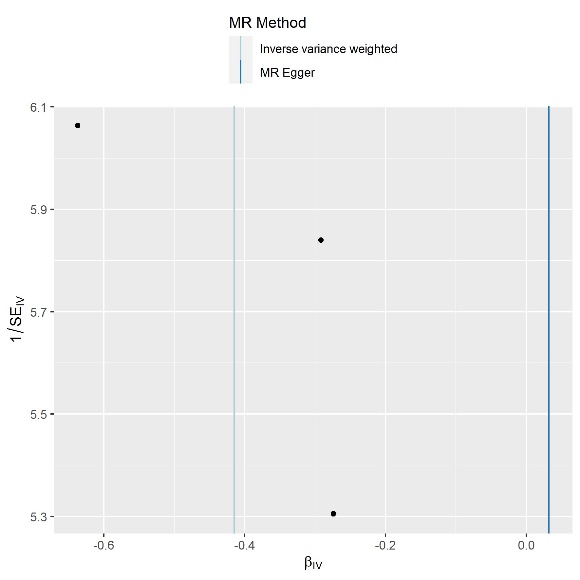


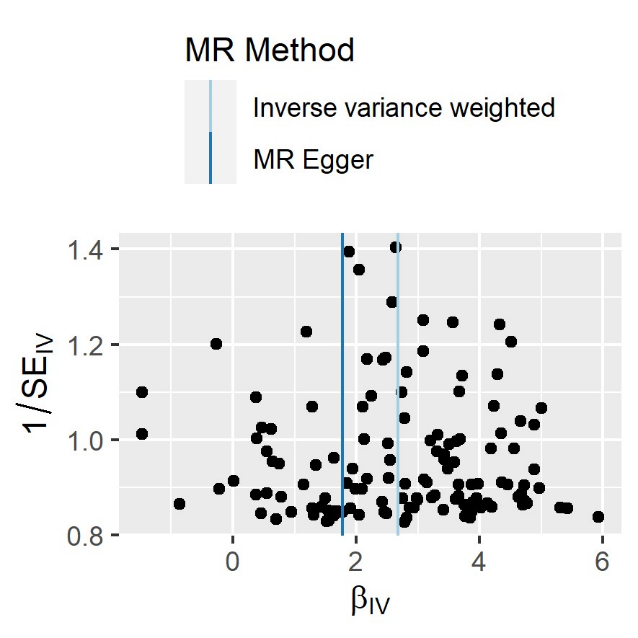


Educational attainment

**Supplementary Figure 32.** Funnel plots used to examine the extent to which pleiotropy is balanced across the instruments used in the univariate Mendelian randomisation of the general factor of SES and indicators of SES on cognitive ability.


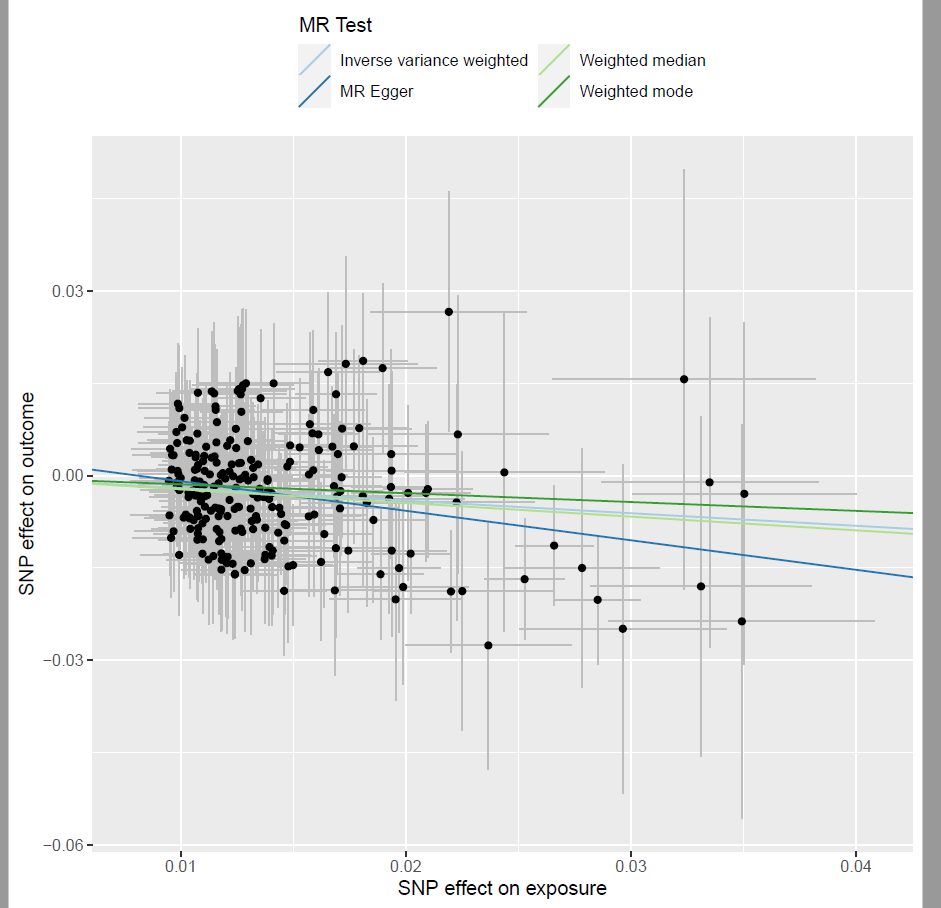

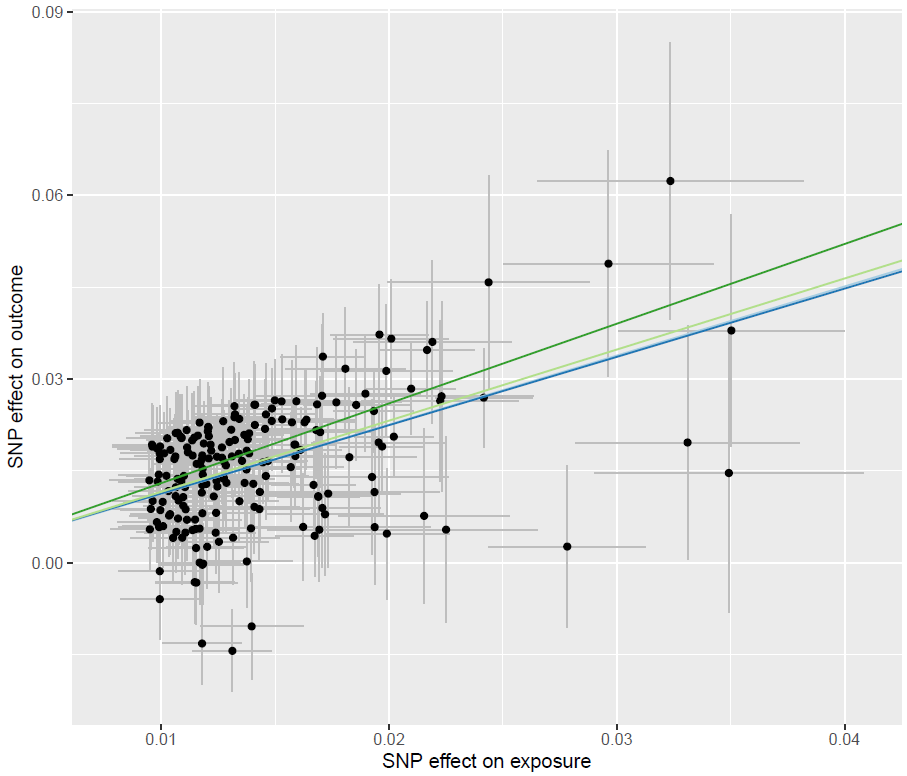


**SNP effect on outcome (Cognitive ability)**

**SNP effect on exposure (SES)**

**Supplementary Figure 33.** The effects of SES on Cognitive ability. The error bars indicate ± standard errors around the estimated phenotype-SNP associations.


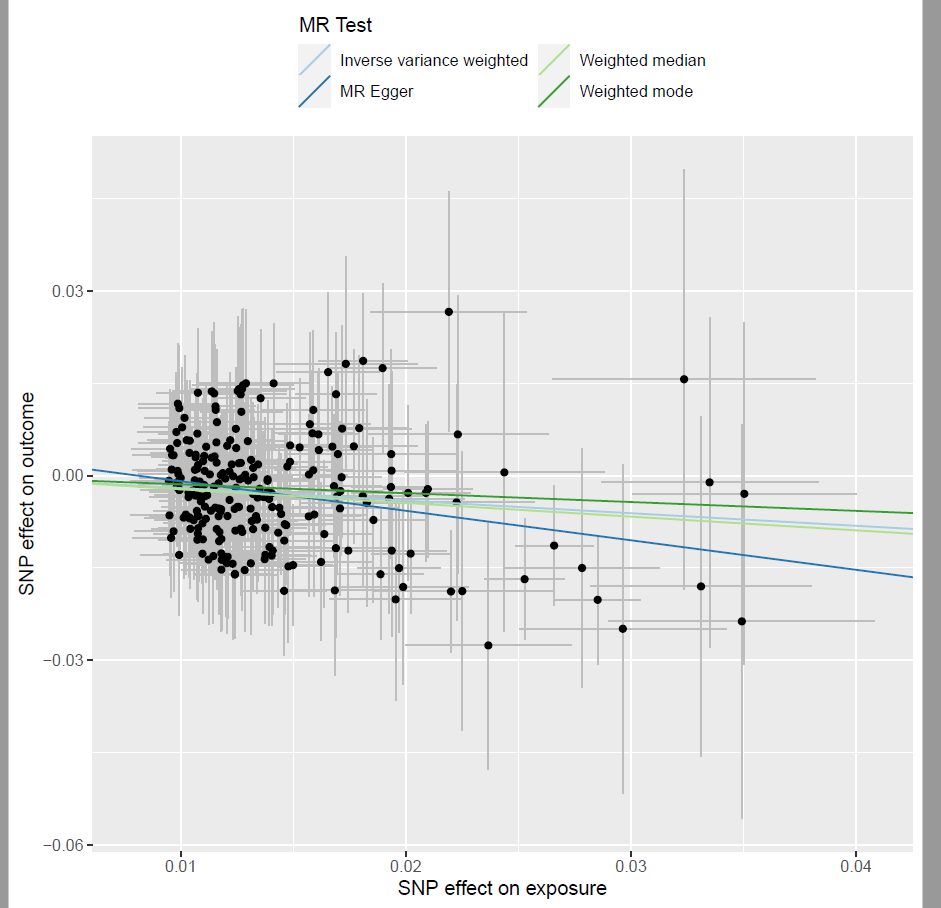

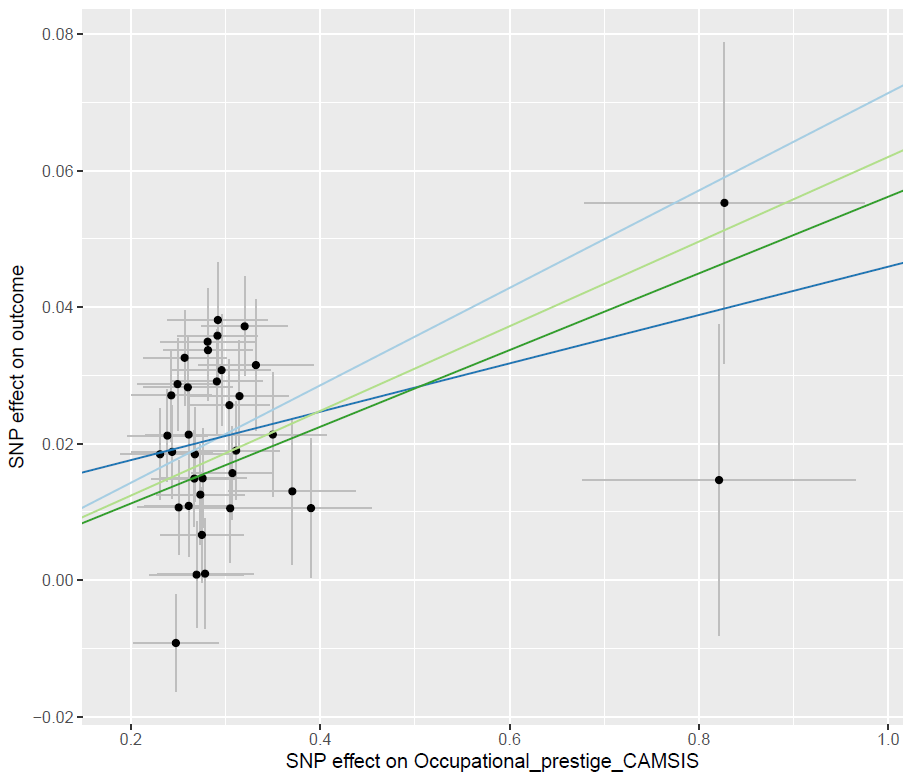


**SNP effect on outcome (Cognitive ability)**

**SNP effect on exposure (Occupation)**

**Supplementary Figure 34.** The effects of Occupation on Cognitive ability. The error bars indicate ± standard errors around the estimated phenotype-SNP associations.


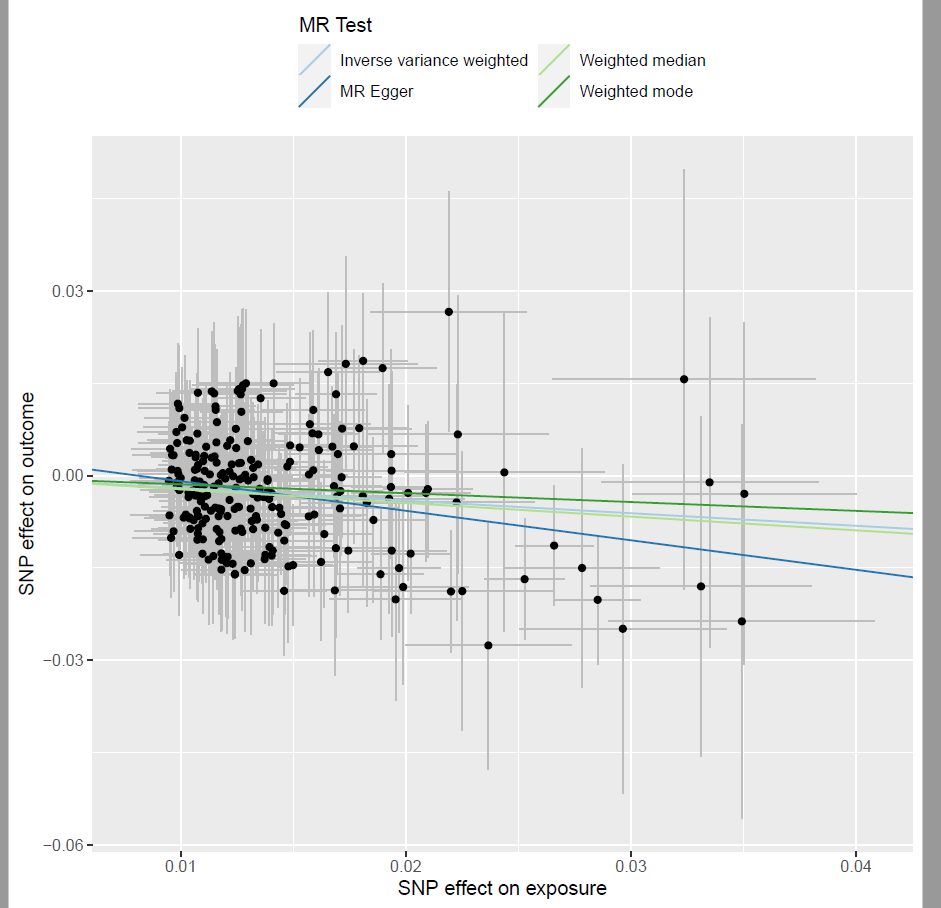

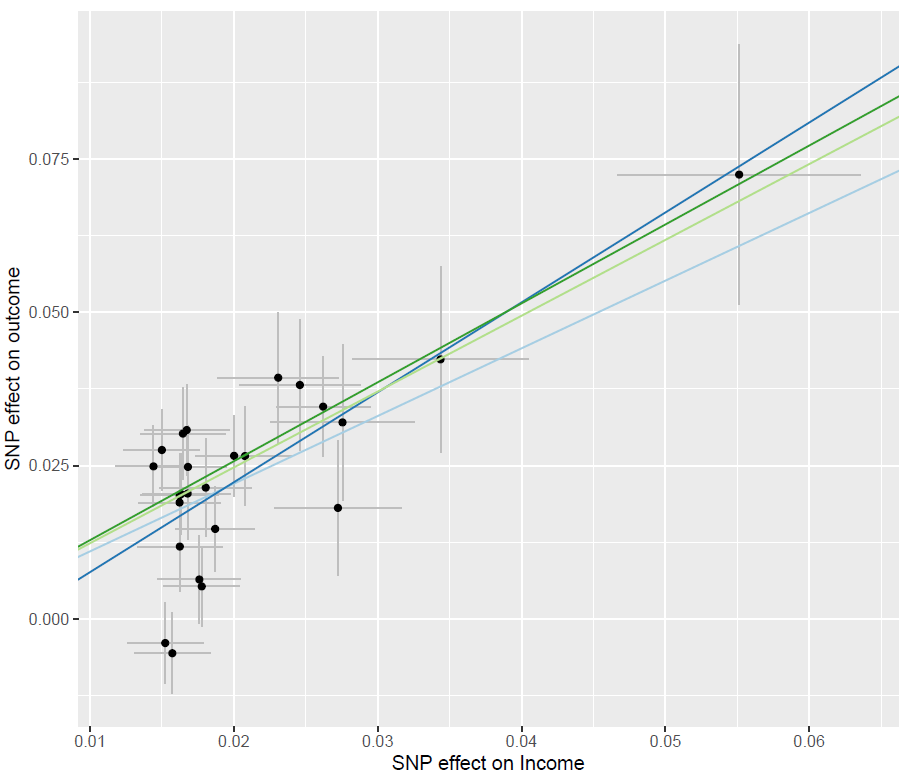


**SNP effect on exposure (Income)**

**SNP effect on outcome (Cognitive ability)**

**Supplementary Figure 35.** The effects of Income on Cognitive ability. The error bars indicate ± standard errors around the estimated phenotype-SNP associations.


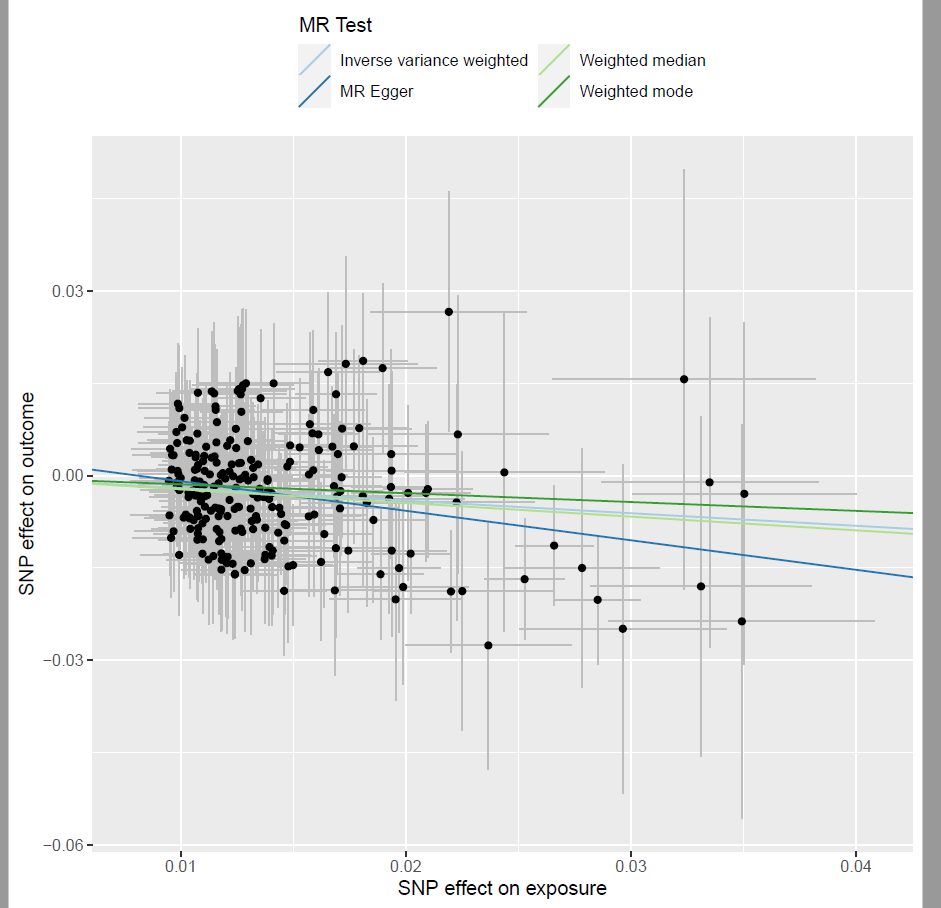

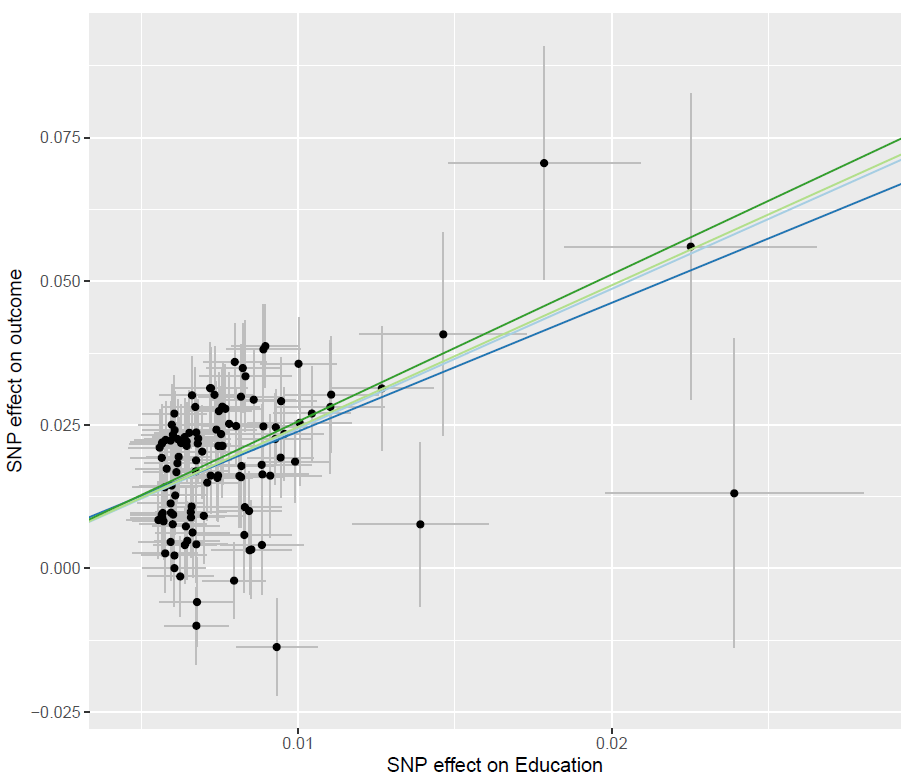


**SNP effect on exposure (Education)**

**SNP effect on outcome (Cognitive ability)**

**Supplementary Figure 36.** The effects of Education on Cognitive ability. The error bars indicate ± standard errors around the estimated phenotype-SNP associations.


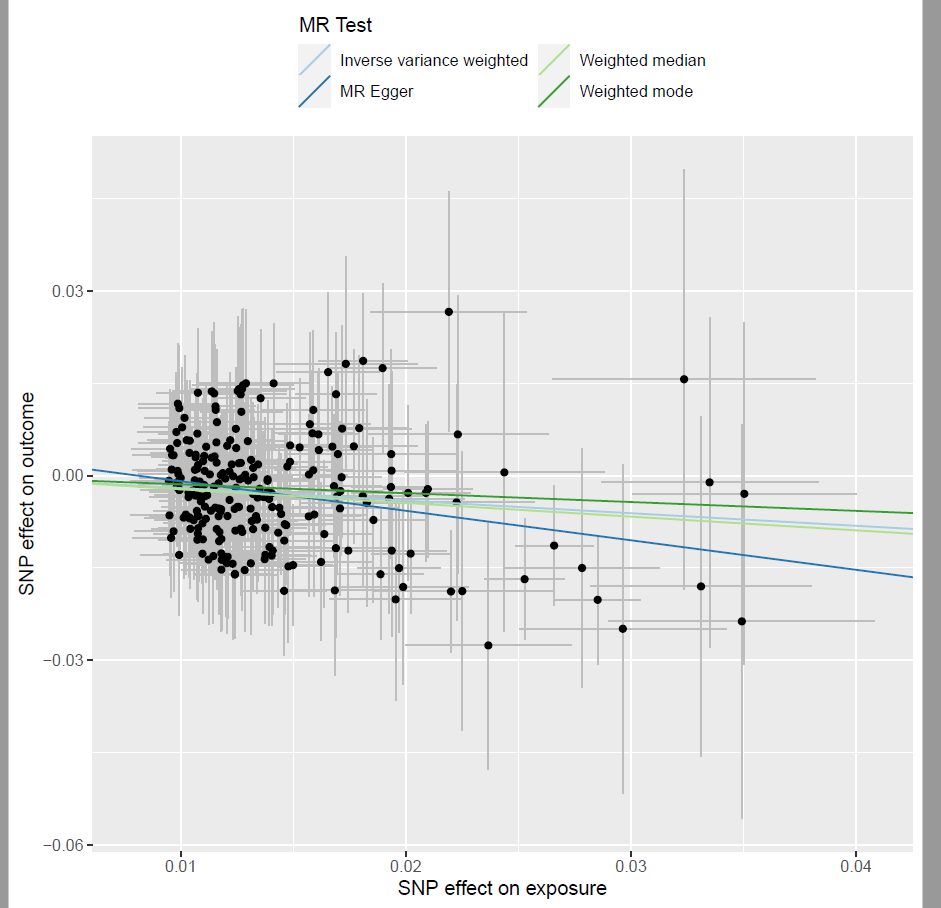

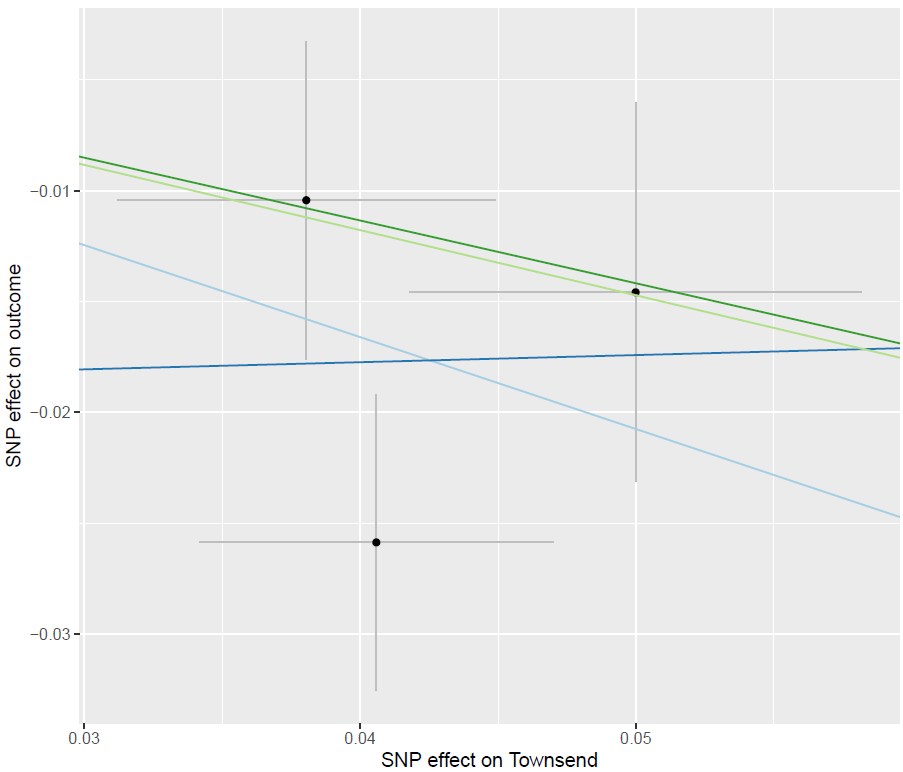


**SNP effect on exposure (Social deprivation)**

**SNP effect on outcome (Cognitive ability)**

**Supplementary Figure 37.** The effects of Social deprivation on Cognitive ability. The error bars indicate ± standard errors around the estimated phenotype-SNP associations.


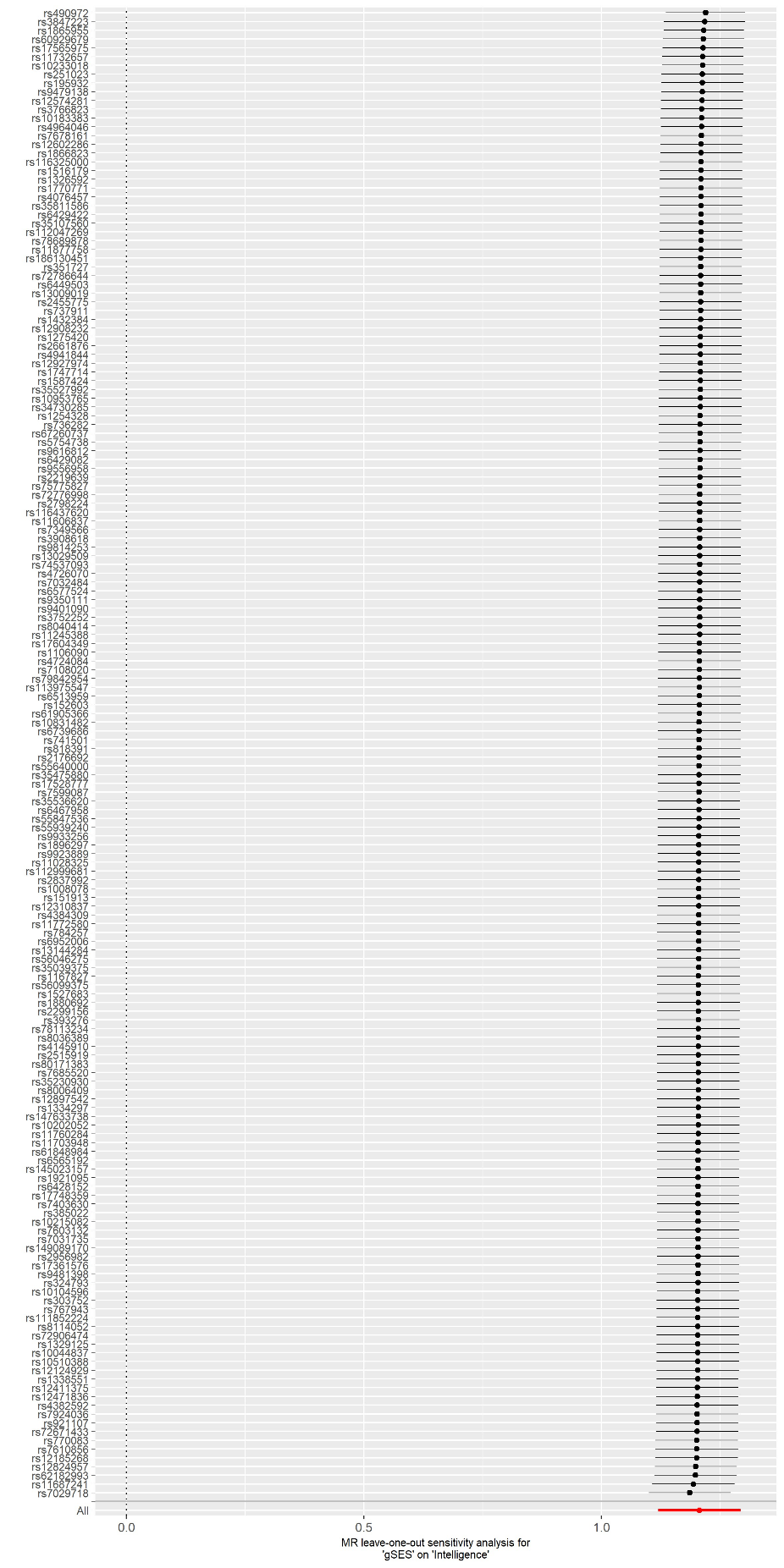


**Leave-one-out sensitivity analysis for SES on Cognitive ability.**

**Supplementary Figure 38.** Leave-one-out plot of the univariate Mendelian randomisation of the general factor of socioeconomic status on cognitive ability.


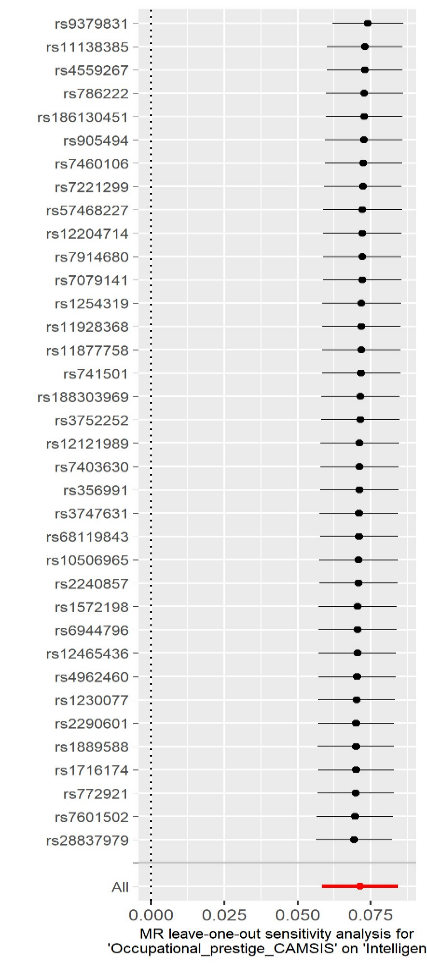


**Leave-one-out sensitivity analysis for Occupation on Cognitive ability.**


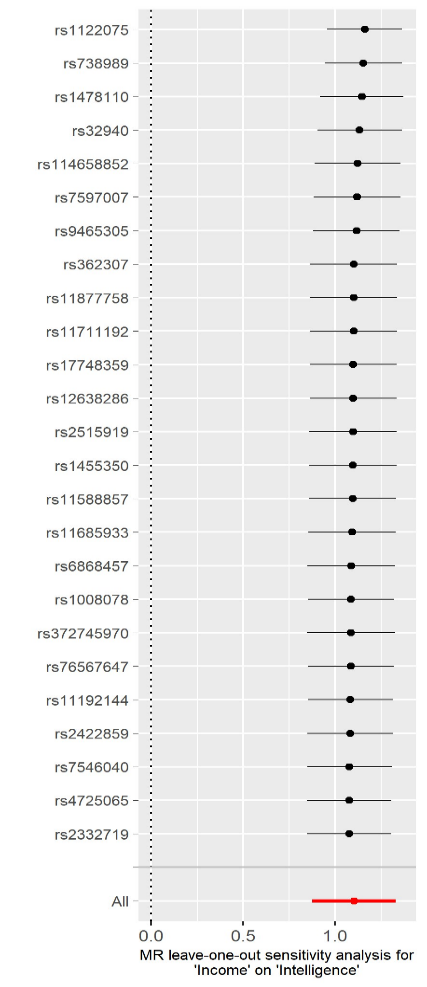


**Leave-one-out sensitivity analysis for Income on Cognitive ability.**

**Supplementary Figure 39.** Leave-one-out plot of the univariate Mendelian randomisation sensitivity analysis of the indicators of Occupation and Income on cognitive ability.


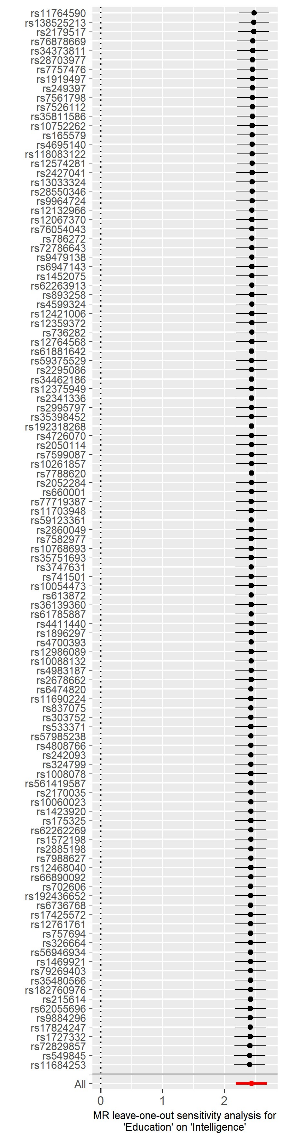


**Leave-one-out sensitivity analysis for Education on Cognitive ability.**

**Supplementary Figure 40.** Leave-one-out plot of the univariate Mendelian randomisation sensitivity analysis of Education on cognitive ability.


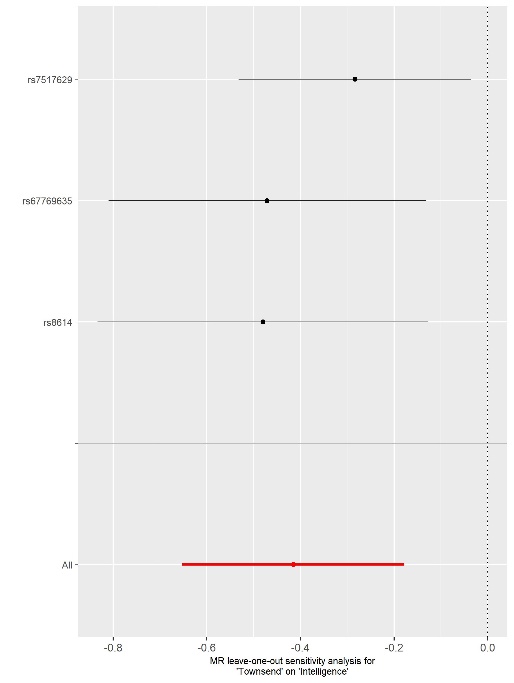


**Leave-one-out sensitivity analysis for Social deprivation on Cognitive ability.**

**Supplementary Figure 41.** Leave-one-out plot of the univariate Mendelian randomisation sensitivity analysis of the Social deprivation on cognitive ability.

## Cognitive ability on brain structure


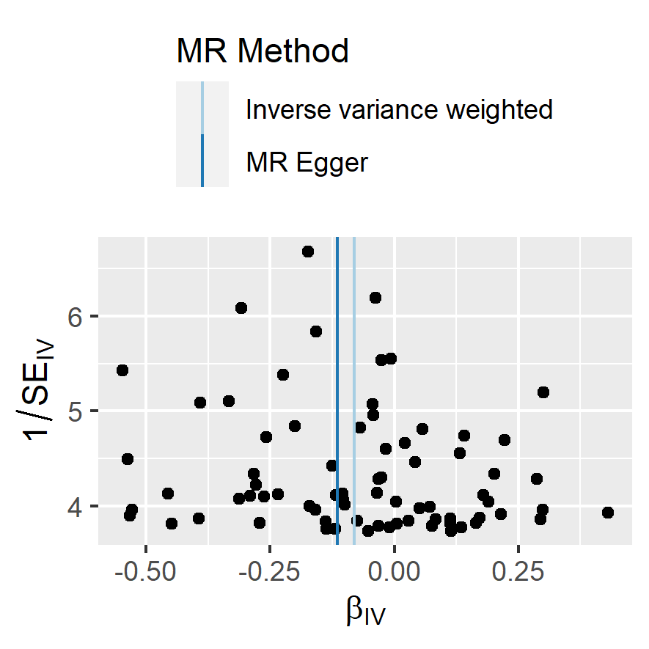


WMHicv


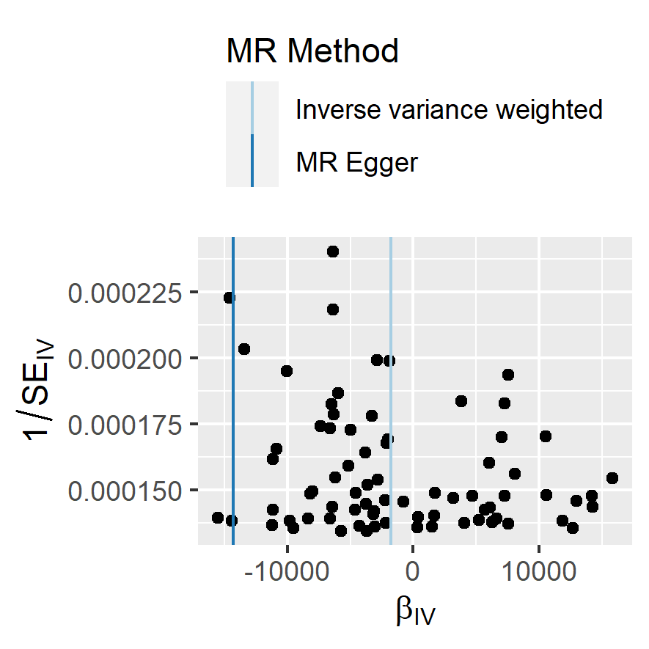


WMicv

**Supplementary Figure 42**. Funnel plots used to examine the extent to which pleiotropy is balanced across the instruments used in the univariate Mendelian randomisation of intelligence on brain structure.

## Total brain volume on cognitive ability.


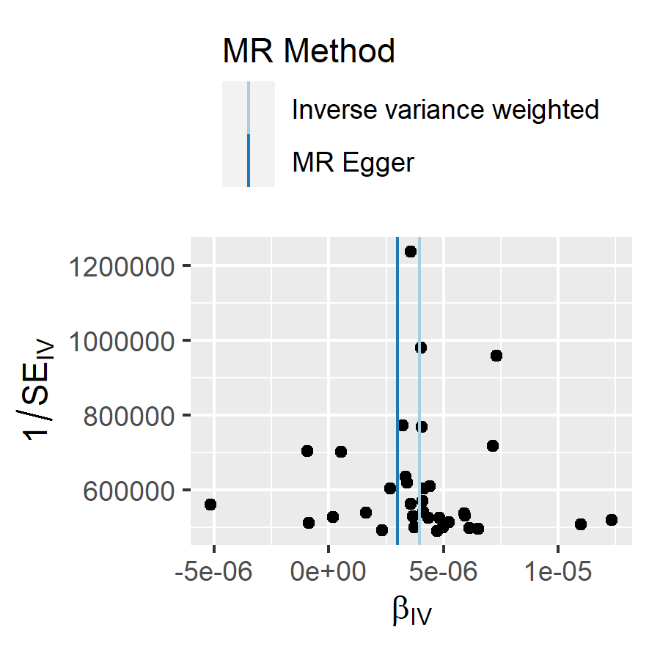


**Supplementary Figure 43.** Funnel plots used to examine the extent to which pleiotropy is balanced across the instruments used in the univariate Mendelian randomisation of total brain volume on cognitive ability.


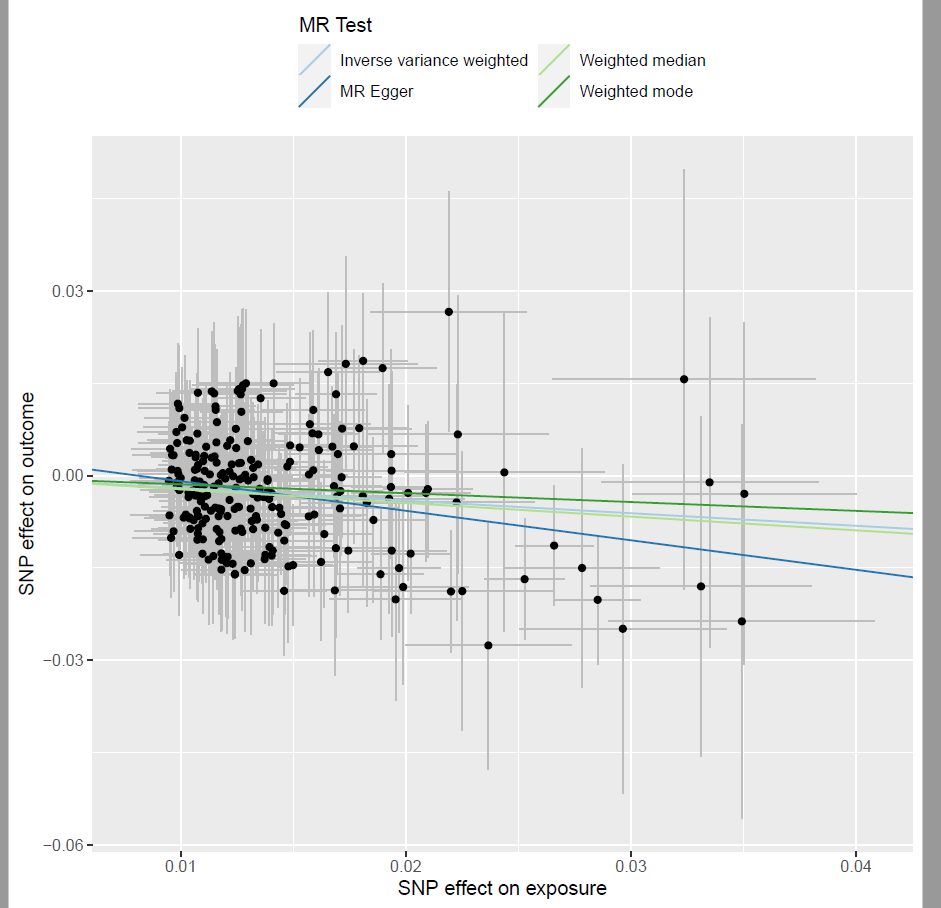

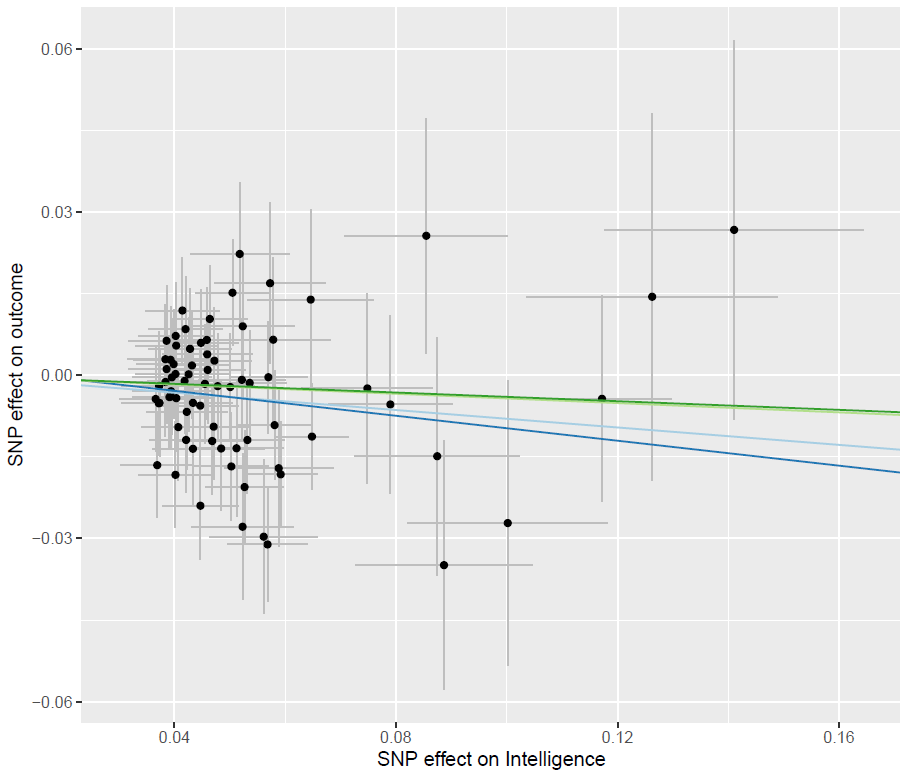


**SNP effect on outcome (WMHicv)**

**SNP effect on exposure (Cognitive ability)**

**Supplementary Figure 44.** The effects of Cognitive ability on WMHicv. The error bars indicate ± standard errors around the estimated phenotype-SNP associations.

**SNP effect on outcome (WMicv)**


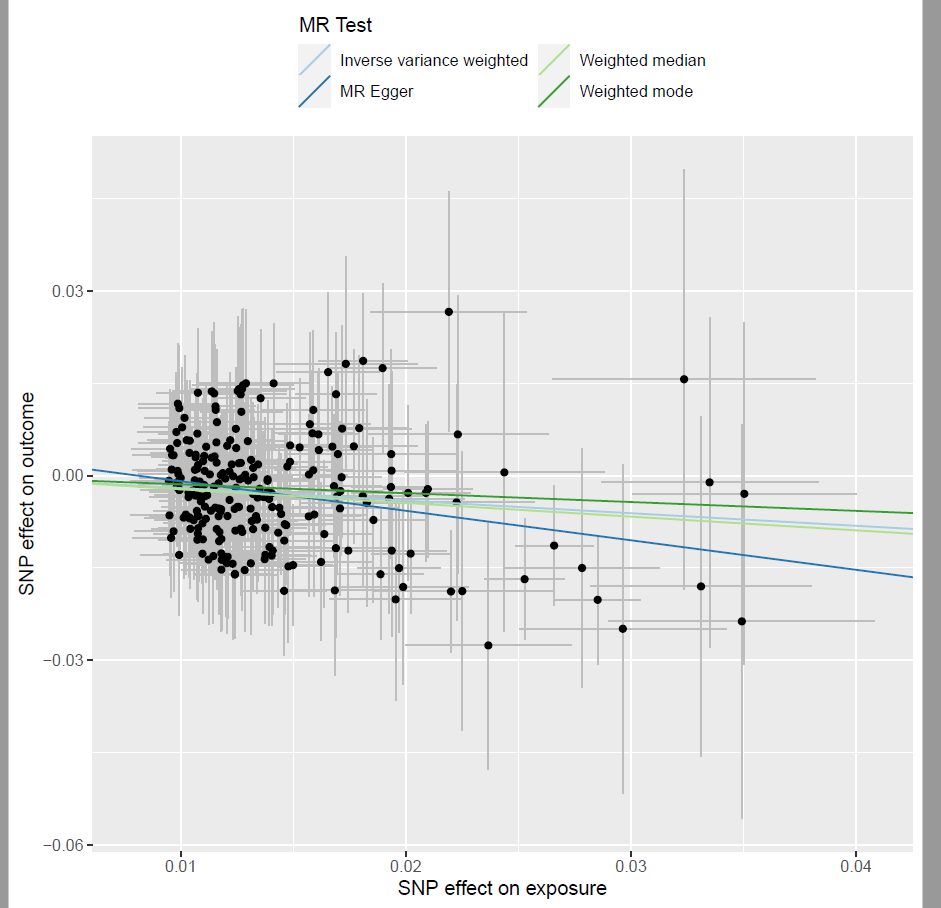

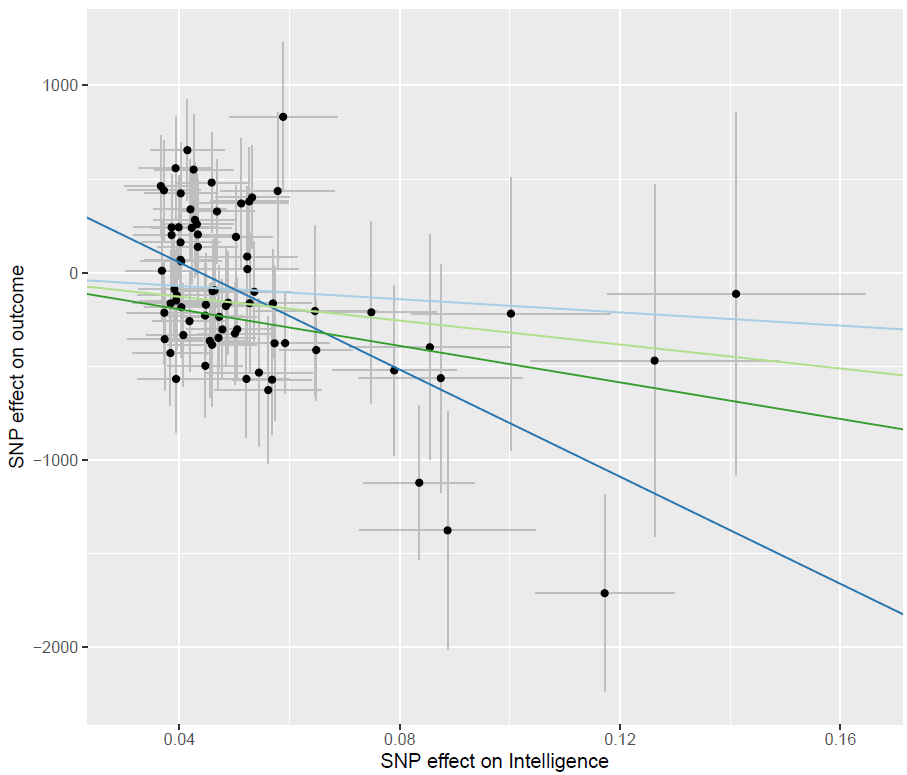


**SNP effect on exposure (Cognitive ability)**

**Supplementary Figure 45.** The effects of Cognitive ability on WMicv. The error bars indicate ± standard errors around the estimated phenotype-SNP associations.


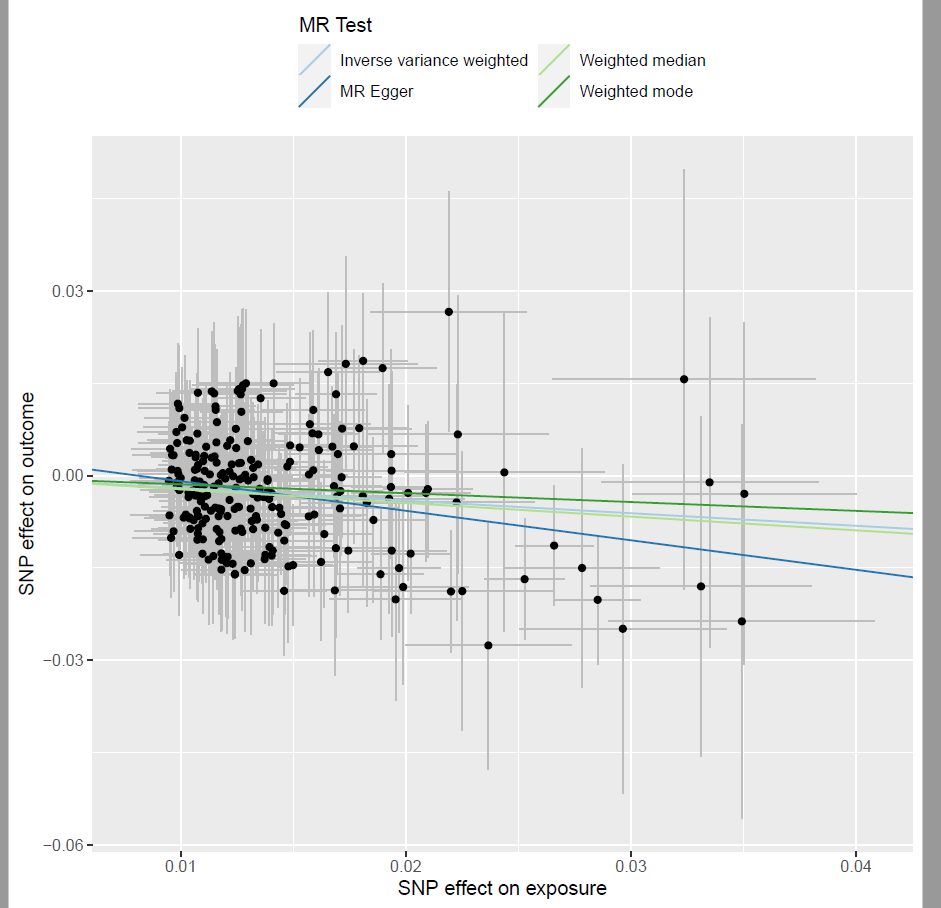

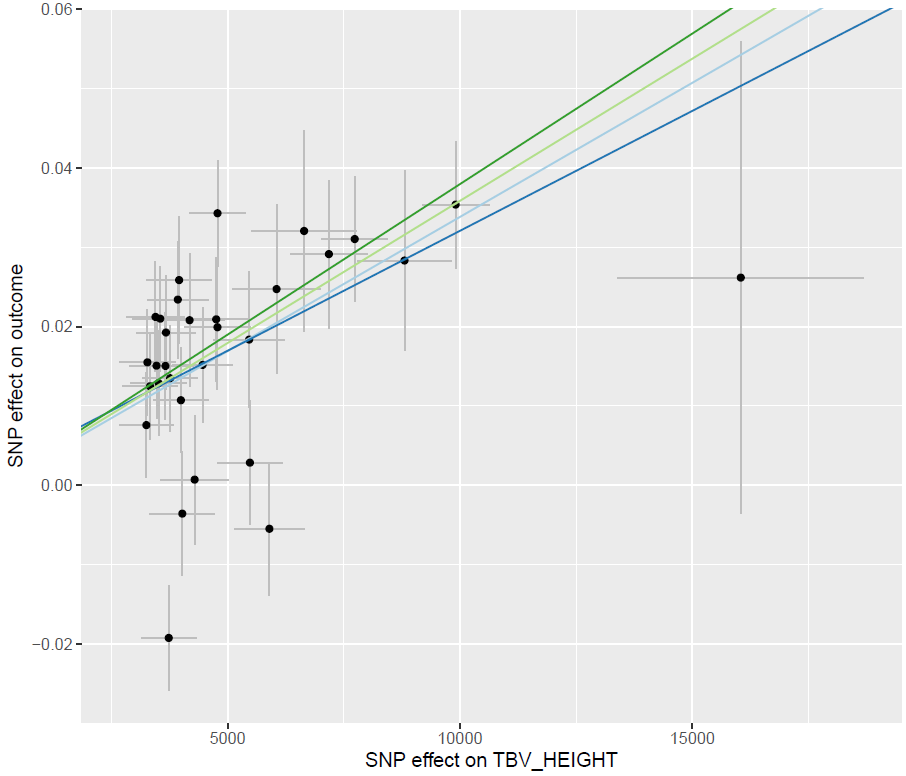


**SNP effect on exposure (TBV)**

**SNP effect on outcome (Cognitive ability)**

**Supplementary Figure 46.** The effects of total brain volume on Cognitive ability. The error bars indicate ± standard errors around the estimated phenotype-SNP associations.


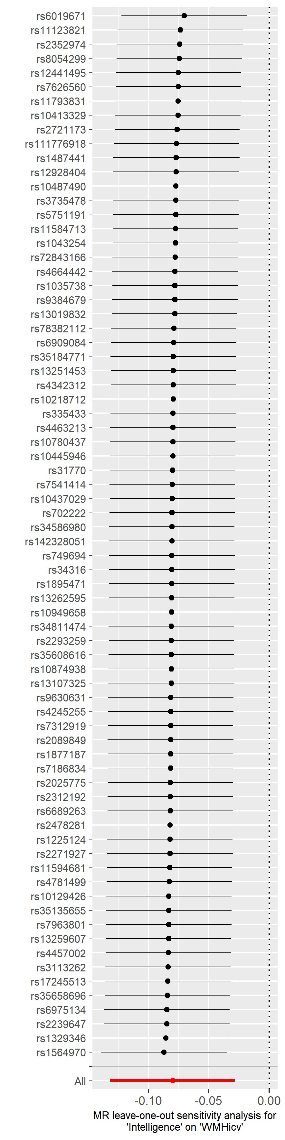

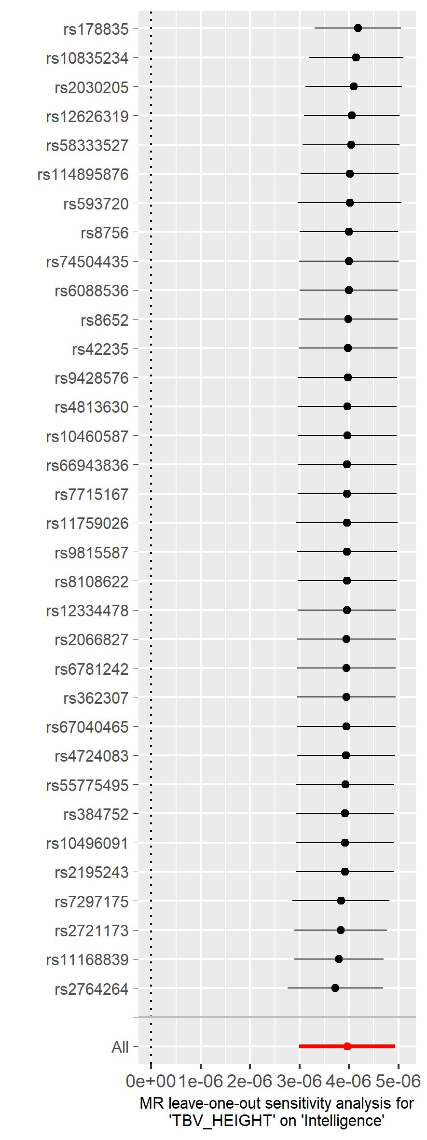


**Leave-one-out sensitivity analysis for Cognitive ability on WMHicv.**

**Leave-one-out sensitivity analysis for TBV on Cognitive ability.**

**Supplementary Figure 47**. Leave-one-out plots of the univariate Mendelian randomisation sensitivity analysis of cognitive ability on WMHicv, and of total brain volume on cognitive ability.
